# When individual life history matters: conditions for juvenile-adult stage structure effects on population dynamics

**DOI:** 10.1007/s12080-018-0374-3

**Published:** 2018-05-04

**Authors:** André M. de Roos

**Affiliations:** Institute for Biodiversity and Ecosystem Dynamics, University of Amsterdam, Amsterdam, The Netherlands

**Keywords:** Population structure, Maintenance requirements, Overcompensation, Population cycles, Emergent community effects

## Abstract

Ecological theory about the dynamics of interacting populations is mainly based on unstructured models that account for species abundances only. In turn, these models constitute the basis for our understanding of the functioning of ecological communities and ecosystems and their responses to environmental change, natural disturbances and human impacts. Structured models that take into account differences between individuals in age, stage or size have been shown to sometimes make predictions that run counter to the predictions of unstructured analogues. It is however unclear which biological mechanisms that are accounted for in the structured models give rise to these contrasting predictions. Focusing on two particular rules-of-thumb that generally hold in unstructured consumer-resource models, one relating to the relationship between mortality and equilibrium density of the consumer and the other relating to the stability of the equilibrium, I investigate the necessary conditions under which accounting for juvenile-adult stage structure can lead to qualitatively different model predictions. In particular, juvenile-adult stage structure is shown to overturn the two rules-of-thumb in case the model also accounts for the energetic requirements for basic metabolic maintenance. Given the fundamental nature of both juvenile-adult stage structure as well as metabolic maintenance requirements, these results call into question the generality of the predictions derived from unstructured models.

**Electronic supplementary material:**

The online version of this article (10.1007/s12080-018-0374-3) contains supplementary material, which is available to authorized users.

## Introduction

Basic models of ecological interactions, such as the Lotka-Volterra competition or predator-prey model, represent populations essentially as collections of elementary particles, subject to replication and mortality only. These so-called unstructured models have also inspired classic textbooks in ecology to define population dynamics as ‘...*the variations in time and space in the sizes and densities of populations*...’, where a population is defined as ‘..*.the number of individuals per unit area*’ (Begon et al. 2005; Turchin 2003). This perspective again emphasises changes in numbers of individuals and neglects differences between them. Accounting for differences in population dynamic models started around almost the same time as Lotka and Volterra introduced their classical models (Kermack and McKendrick 1927; Leslie 1945) but the main focus of these age-structured models has been the effect of individual life history on the exponential growth rate of single populations, rather than the ecological interactions between species.

Consumer-resource interactions constitute the backbone of a community’s food web and are the main type of interactions in ecological communities, which makes understanding them crucial for our understanding of how ecological communities respond to changes in productivity, exploitation or disturbance. For example, one of the best established pieces of ecological theory is the response of food chains, consisting of a linear series of consumer-resource interactions, to changes in system productivity or exploitation rate of the top trophic level (Oksanen et al. 1981). This theory underpins our understanding about and our capacity to predict the community consequences of changes in system productivity (Pace et al. 1999), collapses of dominant fish stocks (Frank et al. 2005) or the re-introduction of top predators (Ripple and Beschta 2012).

From unstructured models in continuous time modelling the interaction of consumer populations foraging exploitatively on shared resources, the following two rules-of-thumb can be distilled that seem to hold universally:

1. *Decreasing density-mortality rule*: an increase in consumer mortality will lead to a decrease in consumer density and an increase in resource density at equilibrium, if the increase in equilibrium resource density does not increase resource production at equilibrium, and
2. *Equilibrium stability rule*: the equilibrium with positive densities of both consumers and resource is stable, if an increase in equilibrium resource density does not increase resource production at equilibrium.

The condition that an increase in equilibrium resource density does not increase resource production is sufficient, but not necessary, for the two rules-of-thumb to hold (see below). The first rule-of-thumb confirms an intuitive expectation that, all else being equal, an increased loss rate is detrimental for the persistence of a species as it decreases its density. This negative relationship between the density and mortality of a species is furthermore an essential ingredient for the occurrence of trophic cascades in communities, where the increase in the density of a predator translates into an increase in predation mortality of its prey and thus to a decrease in the density of this prey. The second rule-of-thumb relates to the inherent tendency of predator-prey interactions to exhibit cycles in population density rather than stable equilibrium coexistence (Bonsall and Hassell 2007). Classical predator-prey cycles, such as predicted by the model of Rosenzweig and MacArthur (1963) only occur, however, in case of a positive relation between prey population growth rate and prey density, such as embodied in exponential or logistic growth, as this allows the prey to escape the top-down control imposed by the predator at equilibrium (de Roos et al. 1990).

Both rules-of-thumb can be straightforwardly validated in a generic, continuous time model for the dynamics of resource and consumer density, *R* and *C*:

1 $$ \left\{\begin{array}{ll} \frac{dR}{d t}\;=\;p(R)\,-\,f(R) C\\[2ex] \frac{dC}{d t}\;=\;\beta f(R) C\,-\,\mu C \end{array}\right.  $$

In these differential equations, the function *p*(*R*) describes the dynamics of the resource in the absence of consumers, while *f*(*R*), *βf*(*R*) and *μ* represent the consumer’s per capita feeding, reproduction and mortality rate. As is common in unstructured consumer-resource models (cf. the Lotka-Volterra predator-prey model and the Rosenzweig-MacArthur model (Rosenzweig and MacArthur 1963)), it is assumed that consumer mortality is independent of consumer and resource density, while feeding and reproduction rate are proportional to each other and increasing functions of resource density ($f^{\prime }(R)>0$). In model (1), the resource density $\tilde {R}$ at equilibrium is determined by the condition $f(\tilde {R})=\mu /\beta $, which implies that $\tilde {R}$ increases with an increase in *μ*, given that $f^{\prime }(R)>0$. The equilibrium consumer density $\tilde {C}$ is related to $\tilde {R}$ following $\tilde {C}=\beta p(\tilde {R})/\mu $. An increase in consumer mortality rate *μ* is therefore guaranteed to decrease the equilibrium consumer density $\tilde {C}$ if the concomitant increase in equilibrium resource density does not increase resource production ($p^{\prime }(\tilde {R})\leq 0$). Furthermore, the consumer-resource equilibrium can be shown to be stable as long as $p^{\prime }(\tilde {R})-f^{\prime }(\tilde {R})\tilde {C}<0$. The condition $p^{\prime }(\tilde {R})\leq 0$ is hence also sufficient to guarantee equilibrium stability, provided that $f^{\prime }(R)>0$. The more restrictive condition that resource production is a non-increasing function of *R* for all resource densities ($p^{\prime }({R})\leq 0$) and not only at the equilibrium resource density will hence also be sufficient for the two rules-of-thumb to hold. Increases in consumer density with increasing consumer mortality, also referred to as a ‘Hydra’ effect, have been reported to occur in unstructured models by Abrams (2009, see also Abrams and Matsuda 2005), but these increases are either due to an increase in resource productivity with an increase in equilibrium resource density ($p^{\prime }(\tilde {R})>0$) or do not pertain to equilibrium densities.

In models of size-structured interactions between consumers and their resource (Persson and de Roos 2013), the rule-of-thumb that increased losses decrease densities has been shown to hold under limited conditions only, despite that resource productivity was assumed to strictly decrease with resource density ($p^{\prime }(R)<0$). These size-structured population models describe the individual life history on the basis of a model of the individual energy budget, in which energy is conserved and energy assimilation from food hence equals the total energy expenditure on growth in body size, metabolic maintenance and reproduction. The models predict that increases in stage-independent mortality may increase equilibrium biomass densities in specific size ranges of the population (de Roos et al. 2007) or even total population biomass (de Roos and Persson 2013). Furthermore, increases in stage-specific mortality, for example of adults only, may increase the equilibrium biomass of the same stage (adults) (de Roos et al. 2007). Empirical evidence for such increases in biomass with increases in (stage-specific) mortality, a phenomenon referred to as ‘biomass overcompensation’, has recently been presented for a variety of different systems (Schröder et al. 2014).

Many population dynamic models that in one way or another account for population age, stage or size structure have furthermore been shown to exhibit so-called single-generation or delayed-feedback cycles in population density that are different from the classical predator-prey cycles (Gurney et al. 1980; Hastings 1984a; Gurney and Nisbet 1985; Murdoch et al. 2002). In contrast to predator-prey cycles, single-generation cycles have a period that is closely related to the generation time of the focal species and result from differential impacts of intraspecific density dependence in different phases of the life history (Gurney and Nisbet 1985). In the case of consumer-resource interactions, these cycles result from differences in competitive ability between juvenile and adult individuals (de Roos and Persson 2003; Persson and de Roos 2013), even when resource productivity only decreases with increasing resource density ($p^{\prime }(R)<0$).

The complexity of the (size-)structured models makes it hard to determine which model assumptions result in the violation of the rules-of-thumb derived from unstructured consumer-resource models. In other words, which biological mechanisms are minimally necessary to result in increases in (stage-specific) biomass with mortality and the occurrence of population cycles. It is therefore unclear whether these phenomena result from basic ecological principles and should occur generally or not. To address this issue, I show here using a simplified but general modelling framework that two basic model ingredients, (1) juvenile-adult stage structure and (2) the energetic costs of somatic maintenance of consumers, are necessary for increases in stage-specific biomass with mortality as well as population cycles to occur. Furthermore, the rules-of-thumb are only overturned if juveniles and adults differ in their efficiency to use acquired resource for their maturation and reproduction, respectively, as only the more efficient life stage will increase in density with increasing mortality. Since juvenile periods and somatic maintenance costs are immutable elements of life, I postulate that the two fundamental rules-of-thumb resulting from unstructured consumer-resource models will only hold under limited conditions, thereby raising doubts about our understanding of community dynamics, based on unstructured, Lotka-Volterra type population models.

## Introducing stage structure

To account for population stage structure the dynamics of the consumer population and its resource will be described with a general, stage-structured extension of model (1), in which the consumer population is subdivided in juveniles and adults with densities *C*_J_ and *C*_A_, respectively. Both stages forage on the shared resource *R* but with different resource-dependent rates *f*_J_(*R*) and *f*_A_(*R*), respectively. Adults and juveniles use assimilation to produce new juvenile offspring at rate *g*_A_(*R*) and to mature to the adult stage at rate *g*_J_(*R*), respectively. Finally, juveniles and adults experience mortality rates of *μ*_J_ and *μ*_A_. The consumer-resource dynamics is then described by the following system of ODEs:

2 $$ \left\{\begin{array}{l} \frac{dR}{d t}\;=\;p(R)\,-\,f_{\mathrm{J}}(R) C_{\mathrm{J}}\,-\,f_{\mathrm{A}}(R) C_{\mathrm{A}}\\[2ex] \frac{dC_{\mathrm{J}}}{d t}\;=\;g_{\mathrm{A}}(R) C_{\mathrm{A}}\,-\,g_{\mathrm{J}}(R) C_{\mathrm{J}}\,-\,\mu_{\mathrm{J}} C_{\mathrm{J}}\\[2ex] \frac{dC_{\mathrm{A}}}{d t}\;=\;g_{\mathrm{J}}(R) C_{\mathrm{J}}\,-\,\mu_{\mathrm{A}} C_{\mathrm{A}} \end{array}\right.  $$

where the function *p*(*R*) is the production rate of the resource in the absence of consumers. In the remainder of this paper, I assume that *f*_J_(*R*), *f*_A_(*R*), *g*_J_(*R*) and *g*_A_(*R*) are all non-decreasing functions of resource density *R* (${f}_{\mathrm {J}}^{\prime }(R)$, ${f}_{\mathrm {A}}^{\prime }(R)$, ${g}_{\mathrm {J}}^{\prime }(R)$, ${g}_{\mathrm {A}}^{\prime }(R) \geq 0$), while at least one of the functions *g*_J_(*R*) and *g*_A_(*R*) is dependent on the resource density and has a strictly positive derivative (either ${g}_{\mathrm {J}}^{\prime }(R)>0$ or ${g}_{\mathrm {A}}^{\prime }(R) > 0$). The latter assumption is to ensure that the model allows for an equilibrium solution. Regarding the resource productivity I will assume that it is constant or decreases with resource density ($p^{\prime }(R)\leq 0$), but that not all three derivatives ${f}_{\mathrm {J}}^{\prime }(R)$, ${f}_{\mathrm {A}}^{\prime }(R)$ and $p^{\prime }(R)$ are simultaneously equal to 0, as this would make the resource dynamics independent of resource density. These conditions guarantee that the classical predator-prey cycles such as found in the Lotka-Volterra or Rosenzweig-MacArthur model do not occur. Any population cycles that do occur will hence result as a consequence of the juvenile-adult stage structure.

An intuitively straightforward approach to model stage structure effects is to take juvenile and adult foraging proportional to the same stage-independent function *f*(*R*), but with different proportionality constants *α*_J_ and *α*_A_ (*f*_J_(*R*) = *α*_J_*f*(*R*) and *f*_A_(*R*) = *α*_A_*f*(*R*)). Analogously, to describe the resource-dependence of the reproduction and maturation rate, the same function *g*(*R*) is used, but with different proportionality constants *β* and *γ*, respectively (*g*_J_(*R*) = *γg*(*R*) and *g*_A_(*R*) = *βg*(*R*)). These assumptions imply that juvenile and adult foraging as well as juvenile maturation and adult reproduction only differ quantitatively from each other, while the functional form of their dependence on the current resource density is qualitatively the same. Irrespective of how plausible these assumptions are, they can be shown to prevent any influence of the population stage structure on the long-term population dynamics if it is furthermore assumed that consumers also experience the same stage-independent mortality rate *μ*.

To assess the consequences of these assumptions, define *C* = *C*_J_ + *C*_A_ as the total number of consumers and *z* = *C*_*J*_/(*C*_J_ + *C*_A_) as the fraction of juveniles in the consumer population. The dynamics of the total consumer density *C* then follows:

$$\begin{array}{@{}rcl@{}} \frac{dC}{d t} &=& g_{\mathrm{A}}(R) C_{\mathrm{A}} - \mu_{\mathrm{J}} C_{\mathrm{J}} - \mu_{\mathrm{A}} C_{\mathrm{A}} \\ &=& g_{\mathrm{A}}(R) (1-z) C - \mu_{\mathrm{J}} z C - \mu_{\mathrm{A}} (1-z) C \end{array} $$

while the dynamics of the fraction of juveniles in the population *z* is given by the following:

$$\begin{array}{@{}rcl@{}} \frac{dz}{d t} &=& \frac{1}{C_{\mathrm{J}}+C_{\mathrm{A}}} \frac{dC_{\mathrm{J}}}{dt} - \frac{C_{\mathrm{J}}}{C_{\mathrm{J}}+C_{\mathrm{A}}} \frac{1}{C_{\mathrm{J}} + C_{\mathrm{A}}} \frac{d(C_{\mathrm{J}}+C_{\mathrm{A}})}{dt}\\ &=& g_{\mathrm{A}}(R) (1-z)^{2} - g_{\mathrm{J}}(R) z - (\mu_{\mathrm{J}} -\mu_{\mathrm{A}}) z (1-z) \end{array} $$

Given the assumptions that *f*_J_(*R*) = *α*_J_*f*(*R*), *f*_A_(*R*) = *α*_A_*f*(*R*), *g*_J_(*R*) = *γg*(*R*) and *g*_A_(*R*) = *βg*(*R*) and *μ*_J_ = *μ*_A_ = *μ*, the consumer-resource model can then be rewritten as follows:

3 $$ \left\{\begin{array}{l} \frac{dR}{d t} = p(R) - (\alpha_{\mathrm{J}} z + \alpha_{\mathrm{A}} (1-z)) f(R) C\\[2ex] \frac{dC}{d t} = \beta g(R) (1-z) C - \mu C\\[2ex] \frac{dz}{d t} = (\beta (1-z)^{2} - \gamma z)g(R) \end{array}\right.  $$

For $t\rightarrow \infty $, *z* will approach its equilibrium value $\bar {z}$, given by the only solution of $\beta (1-\bar {z})^{2}\,-\,\gamma \bar {z}= 0$, for which $0<\bar {z}<1$:

4 $$ \bar{z} = \left( 1+\frac{\gamma}{2\beta} - \sqrt{\left( 1+\frac{\gamma}{2\beta}\right)^{2}-1} \right).  $$

The juvenile fraction *z* will asymptotically approach $\bar {z}$ independent of the dynamics of the resource *R*. Variation in time of *g*(*R*) will influence the rate at which the equilibrium value $\bar {z}$ is reached, but the changes in the population distribution are transient in nature. In the long run, the interaction between the consumer and the resource hence follows the system of ODES:

5 $$ \left\{\begin{array}{l} \frac{dR}{d t} = p(R)\,-\,\bar{\alpha} f(R) C\\[2ex] \frac{dC}{d t} = \bar{\beta} g(R) C\,-\,\mu C \end{array}\right.  $$

in which $\bar {\alpha }=\alpha _{\mathrm {J}}\bar {z}+\alpha _{\mathrm {A}} (1-\bar {z})$ and $\bar {\beta }=\beta (1-\bar {z})$ are weighted parameters characterising consumer intake and reproduction, which are adjusted to take into account the population’s steady-state stage distribution. Clearly, the long-term dynamics of the consumer-resource interaction converges to the long-term dynamics of an unstructured model in terms of resource and total consumer density only. Episodic perturbations in either the resource density or the total consumer density, such as for example a pulsed resource growth process, will not affect this result at all: the consumer-resource dynamics follows the unstructured model (5) after the consumer stage distribution has stabilised. On the other hand, perturbations to the stage distribution of the consumer, for example due to pulsed reproduction of consumers, will have an effect on the consumer-resource dynamics, but this effect will dissipate at a rate given by the last of the system of ODEs (3). Notice that this dissipative effect scales with the value of *g*(*R*). In Appendix A, it is shown that these results even generalise to a consumer population with an arbitrary number of stages.

## Necessary conditions for juvenile-adult stage structure effects

For the general model (2), the equilibrium state is determined by the following set of equations, which has to be solved for the equilibrium resource density $\bar {R}$, the equilibrium juvenile consumer density $\bar {C}_{\mathrm {J}}$ and the adult consumer density in equilibrium $\bar {C}_{\mathrm {A}}$:

6 $$ \left\{\!\begin{array}{ll} H_{1}(\bar{R},\bar{C}_{\mathrm{J}},\bar{C}_{\mathrm{A}})\,=\,p(\bar{R})-f_{\mathrm{J}}(\bar{R}) \bar{C}_{\mathrm{J}}-f_{\mathrm{A}}(\bar{R}) \bar{C}_{\mathrm{A}}&= 0\\[0.5ex] H_{2}(\bar{R},\bar{C}_{\mathrm{J}},\bar{C}_{\mathrm{A}})\,=\,g_{\mathrm{A}}(\bar{R}) \bar{C}_{\mathrm{A}}-g_{\mathrm{J}}(\bar{R}) \bar{C}_{\mathrm{J}}-\mu_{\mathrm{J}} \bar{C}_{\mathrm{J}}\!\!\!\!&= 0\\[0.5ex] H_{3}(\bar{R},\bar{C}_{\mathrm{J}},\bar{C}_{\mathrm{A}})\,=\,g_{\mathrm{J}}(\bar{R}) \bar{C}_{\mathrm{J}}-\mu_{\mathrm{A}} \bar{C}_{\mathrm{A}}&= 0 \end{array}\right.  $$

Without further specification of the functions *f*_J_(*R*), *f*_A_(*R*), *g*_J_(*R*) and *g*_A_(*R*), it is impossible to derive explicit expressions for these equilibrium densities. However, it is possible to asses whether or not an increase in either juvenile or adult consumer density at equilibrium can occur in response to an increase in mortality by applying the implicit function theorem to the system of equilibrium conditions (6). For example, to assess how a change in juvenile mortality changes the equilibrium densities, the conditions (6) can be written as follows:

7 $$ \left\{\begin{array}{l} H_{1}(\bar{R}(\mu_{\mathrm{J}}),\bar{C}_{\mathrm{J}}(\mu_{\mathrm{J}}),\bar{C}_{\mathrm{A}}(\mu_{\mathrm{J}}),\mu_{\mathrm{J}})= 0\\[0.5ex] H_{2}(\bar{R}(\mu_{\mathrm{J}}),\bar{C}_{\mathrm{J}}(\mu_{\mathrm{J}}),\bar{C}_{\mathrm{A}}(\mu_{\mathrm{J}}),\mu_{\mathrm{J}})= 0\\[0.5ex] H_{3}(\bar{R}(\mu_{\mathrm{J}}),\bar{C}_{\mathrm{J}}(\mu_{\mathrm{J}}),\bar{C}_{\mathrm{A}}(\mu_{\mathrm{J}}),\mu_{\mathrm{J}})= 0 \end{array}\right.  $$

which emphasises the fact that the equilibrium densities $\bar {R}$, $\bar {C}_{\mathrm {J}}$ and $\bar {C}_{\mathrm {A}}$ depend indirectly on the mortality rate *μ*_J_ through the dependence of the functions $H_{1}(\bar {R},\bar {C}_{\mathrm {J}},\bar {C}_{\mathrm {A}})$, $H_{2}(\bar {R},\bar {C}_{\mathrm {J}},\bar {C}_{\mathrm {A}})$ and $H_{3}(\bar {R},\bar {C}_{\mathrm {J}},\bar {C}_{\mathrm {A}})$ on *μ*_J_. Differentiation of the system of equations (7) with respect to the juvenile mortality rate, *μ*_J_, results in a linear system of equations that can be solved for the derivatives $d\bar {R}/d\mu _{\mathrm {J}}$, $d\bar {C}_{\mathrm {J}}/d\mu _{\mathrm {J}}$ and $d\bar {C}_{\mathrm {A}}/d\mu _{\mathrm {J}}$, representing the change in equilibrium density of resource, juvenile and adult consumers, $\bar {R}$, $\bar {C}_{\mathrm {J}}$ and $\bar {C}_{\mathrm {A}}$, respectively, with an increase in juvenile mortality, *μ*_J_ (see Appendix B and Online Resource 1 for details).

Similarly, to determine how a change in adult mortality changes the equilibrium densities, the conditions (6) are rewritten as follows:

8 $$ \left\{\begin{array}{l} H_{1}(\bar{R}(\mu_{\mathrm{A}}),\bar{C}_{\mathrm{J}}(\mu_{\mathrm{A}}),\bar{C}_{\mathrm{A}}(\mu_{\mathrm{A}}),\mu_{\mathrm{A}})= 0\\[0.5ex] H_{2}(\bar{R}(\mu_{\mathrm{A}}),\bar{C}_{\mathrm{J}}(\mu_{\mathrm{A}}),\bar{C}_{\mathrm{A}}(\mu_{\mathrm{A}}),\mu_{\mathrm{A}})= 0\\[0.5ex] H_{3}(\bar{R}(\mu_{\mathrm{A}}),\bar{C}_{\mathrm{J}}(\mu_{\mathrm{A}}),\bar{C}_{\mathrm{A}}(\mu_{\mathrm{A}}),\mu_{\mathrm{A}})= 0 \end{array}\right.  $$

in which the equilibrium densities $\bar {R}$, $\bar {C}_{\mathrm {J}}$ and $\bar {C}_{\mathrm {A}}$ are now considered to depend on the mortality rate *μ*_A_ because of the dependence of the functions $H_{1}(\bar {R},\bar {C}_{\mathrm {J}},\bar {C}_{\mathrm {A}})$, $H_{2}(\bar {R},\bar {C}_{\mathrm {J}},\bar {C}_{\mathrm {A}})$ and $H_{3}(\bar {R},\bar {C}_{\mathrm {J}},\bar {C}_{\mathrm {A}})$ on *μ*_A_. Differentiation of the system of equations (8) with respect to *μ*_A_ and solving the resulting linear system of equations results in turn in expressions for $d\bar {R}/d\mu _{\mathrm {A}}$, $d\bar {C}_{\mathrm {J}}/d\mu _{\mathrm {A}}$ and $d\bar {C}_{\mathrm {A}}/d\mu _{\mathrm {A}}$, representing the change in equilibrium density of resource, juvenile and adult consumers, $\bar {R}$, $\bar {C}_{\mathrm {J}}$ and $\bar {C}_{\mathrm {A}}$, respectively, with an increase in adult mortality, *μ*_A_ (see Appendix B and Online Resource 1 for details).

The expressions for the derivatives presented in Appendix B reveal that the derivative $d\bar {C}_{\mathrm {J}}/d\mu _{\mathrm {J}}$ is always negative, independent of parameters and irrespective of the choice of *f*_J_(*R*), *f*_A_(*R*), *g*_J_(*R*), *g*_A_(*R*) and *p*(*R*) (apart from the assumptions regarding their increase or decrease with resource density discussed below equation (2)). In other words, juvenile equilibrium consumer density will always decrease with an increase in juvenile mortality. On the other hand, the derivative of juvenile equilibrium consumer density with respect to adult mortality, $d\bar {C}_{\mathrm {J}}/d\mu _{\mathrm {A}}$, is only guaranteed to be negative, as long as the derivative of the quotient function *f*_A_(*R*)/*g*_A_(*R*) is non-negative:

9 $$ \left( \frac{f_{\mathrm{A}}(\bar{R})}{g_{\mathrm{A}}(\bar{R})}\right)^{\prime} \geq 0  $$

Similarly, the derivatives $d\bar {C}_{\mathrm {A}}/d\mu _{\mathrm {J}}$ and $d\bar {C}_{\mathrm {A}}/d\mu _{\mathrm {A}}$, representing the change in adult equilibrium consumer density with an increase in juvenile and adult mortality, respectively, are both guaranteed to be negative provided that the derivative of the quotient function *f*_J_(*R*)/*g*_J_(*R*) is non-negative:

10 $$ \left( \frac{f_{\mathrm{J}}(\bar{R})}{g_{\mathrm{J}}(\bar{R})}\right)^{\prime} \geq 0  $$

Appendix B furthermore shows how the stability of the equilibrium state can be assessed independent of parameters and irrespective of the choice of *f*_J_(*R*), *f*_A_(*R*), *g*_J_(*R*), *g*_A_(*R*) and *p*(*R*) by analysing the Jacobian matrix of the stage-structured model (2). The Jacobian matrix can be shown to always satisfy the Routh-Hurwitz criterion if the derivatives of the quotient functions *f*_J_(*R*)/*g*_J_(*R*) and *f*_A_(*R*)/*g*_A_(*R*) are non-negative (inequalities (10) and (9)). In this case, the equilibrium state with positive densities of consumers and the resource is always stable.

In summary, if the derivatives of the quotient functions *f*_J_(*R*)/*g*_J_(*R*) and *f*_A_(*R*)/*g*_A_(*R*) are non-negative (inequalities (10) and (9)) any increase in (stage-specific) mortality will decrease the densities of juvenile and adult consumers at and population cycles will not occur. For the juvenile-adult stage structure to overturn either of the two rules-of-thumb discussed in the introduction, it is hence necessary that

11 $$ \left( \frac{f_{\mathrm{J}}(\bar{R})}{g_{\mathrm{J}}(\bar{R})}\right)^{\prime} < 0  $$

and/or

12 $$ \left( \frac{f_{\mathrm{A}}(\bar{R})}{g_{\mathrm{A}}(\bar{R})}\right)^{\prime} < 0  $$

Negativity of the derivative $(f_{\mathrm {J}}(R)/g_{\mathrm {J}}(R))^{\prime }$ indicates that the juvenile maturation rate $g_{\mathrm {J}}(\bar {R})$ at equilibrium increases faster with an increase in resource density than the juvenile foraging rate $f_{\mathrm {J}}(\bar {R})$ at equilibrium. This implies that at a higher resource density juveniles use acquired resources more efficiently for their maturation. In this case, adult consumer density at equilibrium can potentially increase with an increase in either juvenile or adult mortality (or both). Negativity of the derivative $(f_{\mathrm {A}}(R)/g_{\mathrm {A}}(R))^{\prime }$ reflects that the adult reproduction rate $g_{\mathrm {A}}(\bar {R})$ at equilibrium increases faster with an increase in resource density than the adult foraging rate $f_{\mathrm {A}}(\bar {R})$ at equilibrium. This implies that at a higher resource density adult consumers convert acquired resources more efficiently into offspring than at lower resource densities. In this case, juvenile consumer density at equilibrium can potentially increase with an increase in adult mortality. Negativity of either the derivative $(f_{\mathrm {J}}(R)/g_{\mathrm {J}}(R))^{\prime }$ or the derivative $(f_{\mathrm {A}}(R)/g_{\mathrm {A}}(R))^{\prime }$ or both furthermore may result in instability of the equilibrium and the occurrence of population cycles. The increased efficiency with which juvenile or adult consumers at higher resource densities use acquired resources for maturation and reproduction, respectively, is therefore also a necessary condition for the occurrence of cyclic dynamics.

## The joint influence of life history structure and somatic maintenance costs

**Fig. 1:**
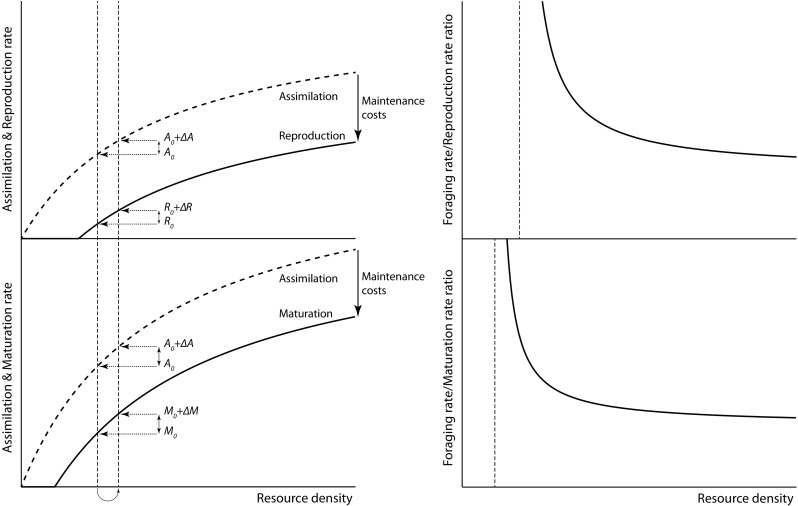
Somatic maintenance costs induce disproportionate responses in reproduction and maturation with increasing resource densities. Left panels: assimilation and net-production rate (top: reproduction by adults; bottom: juvenile maturation) as a function of resource density in case juveniles assimilate resources more efficiently than adults. Maintenance costs lead to a range of resource densities without production and preclude that production scales proportionally with assimilation, such that a small increase in resource density and ingestion can translate into a disproportionally large increase in net-production. For example, if population growth is substantially limited by reproduction (left vertical dotted line), a decrease of 10% in density and redistribution of available resource over the remaining 90% of the individuals, might increase per capita ingestion by only 10% (right vertical dotted line) but roughly double per capita fecundity, resulting in an overall 80% increase in total population reproduction. If juveniles are more efficient in acquiring resources (bottom) this increase in reproduction is disproportionally larger than the increase in juvenile maturation. Right panels: ratio of the adult foraging and reproduction rate *f*_A_(*R*)/*g*_A_(*R*) (top) and the ratio of the juvenile foraging and maturation rate *f*_J_(*R*)/*g*_J_(*R*) (bottom), as derived from the curves shown in the left panels assuming a constant conversion efficiency between foraging and assimilation rate. The vertical dotted lines indicate the threshold resource densities above which reproduction (top) and maturation rate (bottom) are positive

One particular mechanism that will naturally lead to negative derivatives of the quotient functions *f*_J_(*R*)/*g*_J_(*R*) and *f*_A_(*R*)/*g*_A_(*R*) is somatic maintenance. The need to cover energy requirements for somatic maintenance implies that all assimilated food will be used for that purpose when resource densities are low. As a consequence, at low resource densities, both maturation and reproduction rate equal 0 to only turn positive for resource densities above a certain threshold (Fig. 1). The sudden increase in maturation and reproduction rate when resource density exceeds these thresholds makes that the net efficiency of a consumer population changes rapidly and thus that the derivatives of the quotient functions *f*_J_(*R*)/*g*_J_(*R*) and *f*_A_(*R*)/*g*_A_(*R*) are negative (Fig. 1, right panels).

In the unstructured model (1), maintenance costs can be accounted for by reducing the per-capita reproduction rate in the ODE for consumer density with the costs for somatic maintenance, *T*. The per-capita rate *βf*(*R*) is hence replaced by (*βf*(*R*) − *T*)^+^, where the superscript ‘ + ’ is used to indicate that the reproduction rate should be restricted to biologically realistic, non-negative values (i.e. $(\beta f(R)-T)^{+} := \max (\beta f(R)-T, 0)$). In the absence of life history structure, accounting for such maintenance costs does not in any way affect the equilibrium predictions of model (1), since for resource densities close to the equilibrium the reproduction rate *βf*(*R*) − *T* is necessarily always positive and hence (*βf*(*R*) − *T*)^+^ = *βf*(*R*) − *T*. The modified model with maintenance and mortality rate *T* and *μ*, respectively, and the original model (1) with mortality rate *μ* + *T* therefore make the same predictions regarding the changes in equilibrium density with an increase in mortality and the stability of the equilibrium. In other words, in unstructured models maintenance costs can be straightforwardly accounted for by reinterpreting the mortality rate as a loss rate, including both background mortality and maintenance. Of course, the term (*βf*(*R*) − *T*)^+^ may affect dynamics at resource densities well below the equilibrium resource density, but these effects are transient in nature.

In combination with juvenile-adult stage structure, however, accounting for somatic maintenance costs does change the basic rules-of-thumb of population ecology for reasons, which are illustrated in Fig. 1 for the case that reproduction is more resource-limited than maturation. If an increase in mortality leads to a decrease in density with say 10%, this will increase resource density and thereby increase per-capita resource assimilation by juveniles and adults proportionally, thus with also roughly 10%. However, because adults are less efficient in resource assimilation and thereby more resource-limited than juveniles, this increase in assimilation will roughly double adult fecundity. The rate of offspring production by the entire population may consequently increase with up to 80% (90% of the original number of individuals, but each now reproducing at a rate that is 200% of the original reproduction rate). Maintenance costs may thus entail that with the same amount of food distributed over fewer individuals a larger population-level reproduction can be achieved. At the same time, the increase in maturation rate of juveniles will be closer to the increase in their resource assimilation, as for juveniles the difference between the actual resource level and the minimum required for their maturation is larger. The disproportionally large increase in total reproduction rate therefore exceeds the increase in the rates at which individuals leave the juvenile stage through maturation or through mortality. Juvenile density at equilibrium will therefore increase due to an increase in mortality, despite that juveniles experience the higher mortality as well. For analogous reasons, adult density in equilibrium may increase with mortality when population growth is more limited by juvenile maturation than by reproduction.

**Fig. 2:**
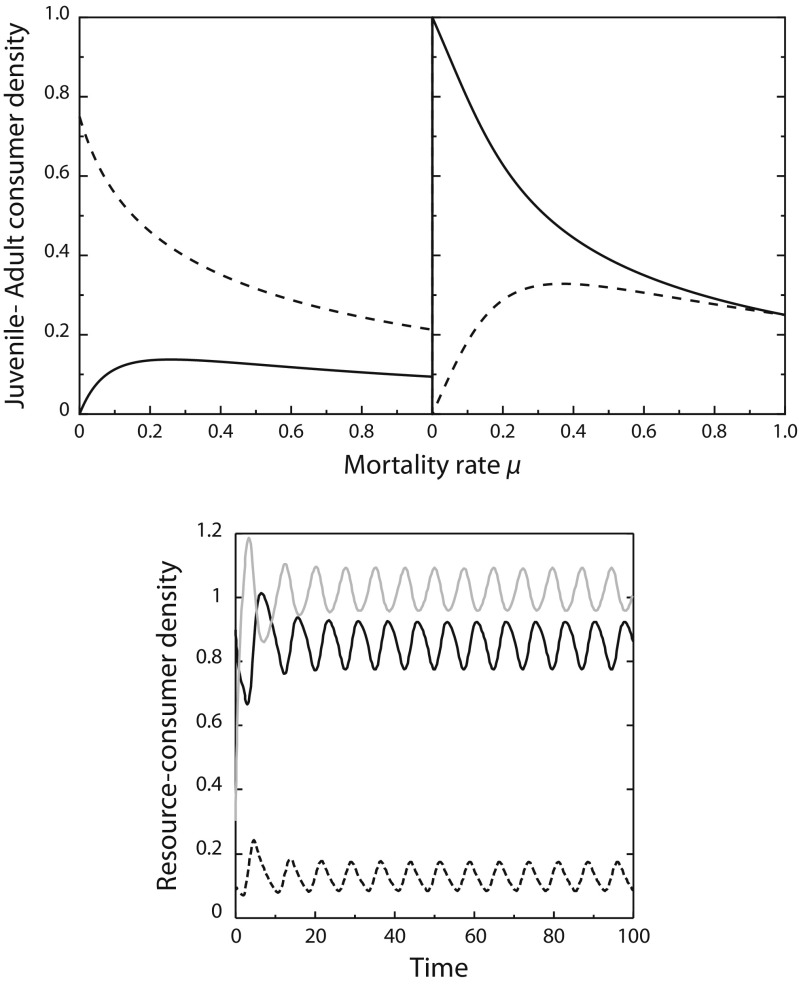
Increases in density with mortality and population cycles due to life history structure and maintenance costs. Top: juvenile (solid line) and adult consumer density (dashed line) in equilibrium of the consumer-resource model (13) as a function of stage-independent mortality *μ*, when juveniles (left, *β* = 0.75) and adults (right, *β* = 1.5) are more efficient in assimilating resource, respectively. Bottom: cycles in resource (grey solid line), juvenile (black solid line) and adult consumer density (black dashed line) occurring for *β* = 2.5 in the consumer-resource model (13). The results shown are based on the assumption of a linear functional response, *f*(*R*) = *R*, and that the parameters *P*, *γ*, and *T* all equal 1.0

Figure 2 (top panels) illustrates using a variant of the consumer-resource model (2) how life history structure and maintenance costs together lead to overcompensation in density when mortality increases. These results are derived assuming that resource productivity is constant *p*(*R*) = *P*, juveniles and adults forage with exactly the same rate, *f*_J_(*R*) = *f*_A_(*R*) := *f*(*R*), experience the same mortality, *μ*_J_ = *μ*_A_ := *μ*, and similar maintenance costs *T*, but potentially differ in their efficiency to assimilate the resource. Adult reproduction is thus described by *g*_A_(*R*) := (*βf*(*R*) − *T*)^+^, whereas juvenile maturation is modelled with *g*_J_(*R*) := (*γf*(*R*) − *T*)^+^. The particular model is therefore captured by the differential equations:

13 $$ \left\{\begin{array}{l} \frac{dR}{d t} = P - f(R)\,(C_{\mathrm{J}}+C_{\mathrm{A}})\\[2ex] \frac{dC_{J}}{d t} = (\beta f(R)-T)^{+} C_{\mathrm{A}} - (\gamma f(R)-T)^{+} C_{\mathrm{J}} - \mu C_{\mathrm{J}}\\[2ex] \frac{dC_{\mathrm{A}}}{d t} = (\gamma f(R)-T)^{+} C_{\mathrm{J}} - \mu\, C_{\mathrm{A}} \end{array}\right.  $$

**Fig. 3:**
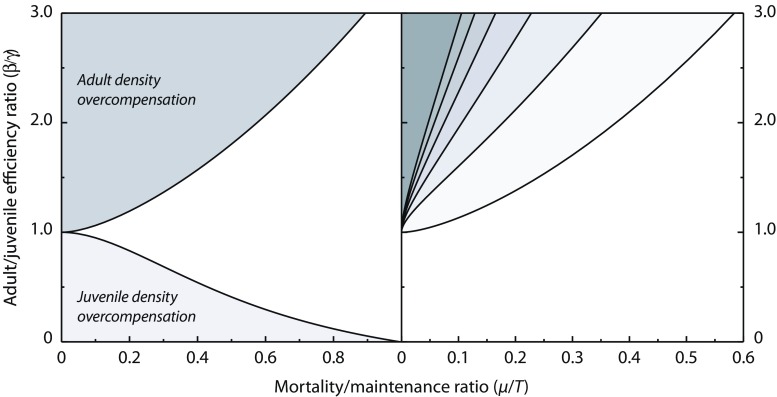
Parameter ranges with overcompensation and population cycles. Parameter ranges for which overcompensation in juvenile and adult density (left) and population cycles (right) occur in the consumer-resource model (13) as a function of the ratio between mortality and maintenance rate and the ratio between adult and juvenile assimilation rate. For parameter combinations *μ*/*T* and *β*/*γ* in the white region of the right panel the equilibrium is always stable, whereas the light to dark grey regions in this panel indicate parameter combinations for which increasingly larger values of resource productivity are required for equilibrium stability (black contour lines indicate from low to high the stability boundaries for $\gamma P f^{\prime }(\bar {R})/T^{2} = 0, 1, 2, \ldots ,5$, respectively). The results shown are independent of the form of the functional response *f*(*R*) and parameter values

By applying the implicit function theorem to the system of equations determining the equilibrium densities $\bar {R}$, $\bar {C}_{\mathrm {J}}$ and $\bar {C}_{\mathrm {A}}$ of the model, it is shown in Appendix C that the nature and extent of the density overcompensation (i.e. the increases in equilibrium juvenile or adult density with increasing mortality) are completely determined by the adult-juvenile ratio of resource assimilation, *β*/*γ*, and the relative loss rate through mortality compared to maintenance, *μ*/*T*. Increases in the equilibrium density of juvenile consumers with mortality can occur when reproduction is more limited by resources than maturation, *β*/*γ* < 1, and as long as the loss rate through maintenance exceeds the mortality losses, *μ*/*T* < 1 (Fig. 3, left panel). On the other hand, increases in the equilibrium density of adult consumers with mortality can occur when maturation is more limited by resources than reproduction, *β*/*γ* > 1. These increases also occur predominantly when the loss rate through maintenance exceeds mortality losses (Fig. 3, left panel), but for very high adult-juvenile efficiency ratios, they also occur when mortality losses are larger than maintenance. Figure 2 provides examples of these increases in juvenile and adult density in equilibrium for particular parameter combinations. The maximum equilibrium density occurs when mortality losses are in the order of 20–30% of the losses through somatic maintenance, independent of whether the population is more limited by reproduction or by maturation (Fig. 2). These increases in equilibrium density of juvenile and adult consumers can also occur when only juveniles or adults are exposed to a higher mortality rate (Online Resource 2, Sections II.4 and II.5). Increases in juvenile mortality can increase adult equilibrium density when maturation is more limited by resources than reproduction, but will never increase juvenile equilibrium density. Increases in adult mortality, on the other hand, can increase juvenile as well as adult equilibrium density, depending on whether reproduction or maturation is more resource-limited. The increases in equilibrium density of adults with increases in their mortality clearly violates the rule-of-thumb that higher loss rates decrease densities.

In Appendix C, it is furthermore shown that the Jacobian matrix of the specific model (13) can violate the Routh-Hurwitz criterion even though resource production is independent of resource density ($p^{\prime }(R)= 0$). This implies that the combination of life history structure and somatic maintenance costs can also lead to instability of the consumer-resource equilibrium and the occurrence of population cycles, dependent again on the adult-juvenile ratio of resource assimilation and the relative loss rate through mortality compared to maintenance. In case the equilibrium is unstable, the consumer-resource system exhibits antiphase population cycles with approximately a half-period phase lag between consumer and resource densities, as shown in Fig. 2 (lower panel). Cycles only occur when maturation is more limited by resources than reproduction, *β*/*γ* > 1, and losses through maintenance exceed the mortality losses, *μ*/*T* < 1 (Fig. 3, right panel). Furthermore, the occurrence of population cycles also depends on the resource productivity and the sensitivity of the maturation rate at equilibrium to changes in resource density, both expressed relative to the somatic maintenance rate. For larger values of the resource productivity, the equilibrium is always stable; hence, population cycles are primarily expected to occur when productivity is low.

## Discussion

The models discussed in this paper are phenomenological and hence only mimic to a very limited extent any real ecological system. In fact, they are as phenomenological as the basic Lotka-Volterra predator-prey model, but extend the latter with a juvenile-adult stage structure. The juvenile-adult stage structure is shown to overturn the decreasing density-mortality and equilibrium stability rules-of-thumb if and only if either the maturation rate increases faster than the juvenile foraging rate in response to an increase in resource density (inequality (11)) or if the reproduction rate increases faster than the adult foraging rate as a consequence of the resource density increase (inequality (12)). Accounting for the energetic requirements for somatic maintenance automatically results in such increases in resource utilisation efficiency for both juveniles and adults.

Disproportionally large increases in maturation or reproduction rate compared to the increase in juvenile and adult foraging rate, respectively, occur in particular at resource densities just above the break-even density, where the resource intake of a juvenile or adult is just sufficient to cover its basic maintenance requirements (Fig. 1). This threshold resource density is also referred to as critical resource density or maintenance resource density (Persson et al. 1998; Persson and de Roos 2013). When consumer mortality is low and competition for resources consequently intense, the consumer population can be expected to equilibrate at resource densities just above the juvenile or the adult critical resource density, whichever is the smaller of the two. Changes in population structure with increasing mortality can thus be expected to occur especially at low background mortality levels.

Juvenile-adult stage structure and energetic costs for basic maintenance requirements are hence the two necessary model ingredients that give rise to the increases in stage-specific or total population biomass reported for size-structured models (de Roos et al. 2007; Persson and de Roos 2013; de Roos and Persson 2013) and the single-generation cycles in population density found in many age-, stage- or size-structured models (Gurney et al. 1980; Gurney and Nisbet 1985; Murdoch et al. 2002; de Roos and Persson 2003). These two biological mechanisms are furthermore immutable elements of life: every organism experiences a delay between its birth and the onset of its reproduction and furthermore needs energy to sustain its basic life functions and to fuel the inevitable turn-over of its tissues. A certain amount of the energy an individual assimilates is hence not used for increasing its soma or for the production of offspring, but simply for ensuring its own persistence. The combination of these two immutable elements of life overturns the two basic rules-of-thumb focused on in this paper: the negative relationship between equilibrium density and mortality and the stability of the consumer-resource equilibrium as long as resource productivity is not increasing with an increase in resource density. Since the rules-of-thumb are central to a considerable body of basic ecological theory, in particular with respect to the occurrence of trophic cascades and top-down effects in food webs, the results presented here call into question the generality of this basic ecological theory.

The influence of population stage structure is shown to be completely irrelevant for the long-term population dynamics if juveniles and adults experience the same mortality rate, their foraging rates scale with resource density following the same function *f*(*R*) and, similarly, maturation and reproduction rate scale with resource density following the same function *g*(*R*). Juvenile and adult foraging rates in this case only differ from each other by a constant multiplication factor, while the same holds for the maturation and reproduction rates. If these conditions apply, the dynamics of the population stage structure decouples from the dynamics of the total consumer density and the stage structure converges to a steady-state distribution irrespective of the changes in resource and consumer density. These conditions resemble the conditions that lead to ontogenetic symmetry in energetics, as presented in the context of size-structured consumer-resource dynamics by de Roos et al. (2013). Instead of size structure and biomass densities, the focus here is on numerical abundances of consumers in the different stages, disregarding any energetic aspects. Nonetheless, the conditions that eliminate the influence of the population structure on the dynamics of the total population have the same interpretation as described by de Roos et al. (2013): all consumer stages are to the same extent limited by food in their performance and experience the same per-capita mortality rate. More generally, it can thus be concluded that any impact of consumer population structure on consumer-resource dynamics can only come about in case of *ontogenetic asymmetry*, which occurs when individuals in different life stages either respond differently to changes in resource density or differ in the mortality rate they experience. Indeed, the analysis in this paper also shows that energetic differences between juveniles and adults are a prerequisite for the occurrence of positive relationships between equilibrium density and mortality as well as the occurrence of population cycles (Fig. 3).

While models accounting for juvenile-adult stage structure have been studied frequently (e.g. Hastings 1983, 1984b; Briggs 1993; Olson et al. 1995; Revilla 2000; Abrams and Quince 2005), population dynamic models that account for basic maintenance requirements are more rare except for the model proposed by Yodzis and Innes (1992). More often, the maturation and reproduction rate of a consumer tends to be taken proportional to the juvenile and adult foraging rate, respectively, as exemplified by the model studied by Schreiber and Rudolf (2008), which captures juvenile maturation with a phenomenological description. The linear relationship between a consumer’s numerical and functional response is referred to as the linear conversion rule (Ginzburg 1998), which is considered a sensible assumption that is effectively adopted in most if not all unstructured models (Ginzburg 1998; Arditi and Ginzburg 1989). Accounting for maintenance costs, however, necessarily leads to deviations from this linear conversion rule.

Some predictions of the generic model (2) differ from those of the more complicated size-structured models for which positive relationships between equilibrium density and mortality were reported first. In particular, juvenile equilibrium density is shown to decrease with increasing juvenile mortality in the stage-structured model (2), whereas in stage-structured biomass models a positive relationship between juvenile equilibrium biomass density and mortality has been shown to occur (de Roos et al. 2007). Such increases have furthermore been firmly corroborated in multi-generational laboratory experiments with self-sustaining populations of least killifish (*Heterandria formosa*) kept under controlled food supply conditions (Schröder et al. 2009). This discrepancy in predictions between the two model frameworks implies that the growth in body size of juveniles, which is accounted for in stage-structured biomass models (de Roos et al. 2007) but not in the stage-structured model (2), plays a crucial role. Indeed, if the equilibrium resource density is just above the critical resource density, at which juvenile ingestion exactly covers juvenile maintenance costs, an increase in resource density in the stage-structured model (2) only translates into a disproportionally large increase in the rate at which individuals leave the juvenile stage and mature into the adult stage. However, if the model would account for growth in body size of juveniles, the disproportionally large increase would occur as well in the production rate of new biomass through somatic growth, thus explaining why juvenile biomass may indeed increase with increases in juvenile mortality in stage-structured biomass or size-structured models (de Roos et al. 2007; de Roos and Persson 2013).

As another discrepancy, the stage-structured model (2) predicts that only a single type of population cycles arises, whereas in previously analysed size- and stage-structured models, two different types of single-generation cycles have been found to occur (de Roos and Persson 2003; 2013), driven by either juvenile or adult superiority in the competition for resources. When juveniles are competitively superior, juvenile-driven cycles result that are characterised by a single cohort dominating the population dynamics throughout its lifetime and a strongly varying population size or stage distribution with either juveniles or adults making up the largest part of the population. Consequently, in juvenile-driven cycles, reproduction tends to be limited to a short time interval at the start of a population cycle. In contrast, adult-driven cycles, which occur when adults are competitively superior to juveniles in the competition for resources, are characterised by a more stable population size or stage distribution (de Roos and Persson 2003, 2013) and reproduction occurring more continuously throughout the entire population cycle. Since the cycles found in the stage-structured model (2) only occur when adults are more efficient in their use of resources for reproduction than juveniles use ingested resources for maturation (*β*/*γ* > 1; Fig. 3), they likely correspond to adult-driven cycles arising from adult superiority in resource competition. Juvenile-driven cycles are unlikely to occur in the stage-structured model (2) as the model structure does not allow for the formation of distinct cohorts that can dominate the population throughout their life.

An important characteristic of the consumer-resource cycles shown in Fig. 2 is the phase difference between the resource and especially the juvenile consumer density of approximately half a cycle period. Such antiphase cycles have been argued to be the hallmark of predator-prey cycles driven by rapid evolution in the prey (Yoshida et al. 2003) but occur here as a consequence of asymmetric juvenile-adult competition for resources. Juveniles dominate the consumer population and suppress the resource density when their density increases. This contributes to further increases in juvenile density as it slows down their own recruitment to the adult stage, while adults use acquired resources more efficiently and hence continue to reproduce despite the low resource density. The lack of adult recruitment eventually leads to a decrease in total reproduction and a decline in juvenile consumer density.

The most important consequence of the positive relationships between consumer density and mortality are their implications for higher trophic levels and community structure. For example, a generalist predator foraging on both juveniles and adults would impose stage-independent predation mortality. If this predation mortality increases either juvenile or adult consumer density in equilibrium, the generalist predator indirectly increases the food availability for a stage-specific predator that specialises on either juvenile or adult consumers. Density overcompensation may hence lead to facilitation between generalist and stage-specific predators, but also between two predators that specialise on different life history stages of consumers (de Roos et al. 2008). As shown in Online Resource 2 (Section II.4), an increase in juvenile mortality alone can lead to increases in adult equilibrium density if adults are more efficient in their resource use (*β*/*γ* > 1). If the increased mortality would be imposed by a predator specialising on juvenile consumers, it would facilitate the persistence of a predator specialising on adult consumers as an indirect consequence of the positive density-mortality relationship. Similarly, predators specialising on adult consumers can promote the persistence of predators specialising on juveniles in case juvenile consumers are more efficiently using resources for maturation than adults use their ingested resources for reproduction (Online Resource 2, Section II.5). Clearly, because of this facilitation the diversity of the predator guild, exploiting a particular consumer species in different phases of its life is important for the persistence of its guild members: if the specialist predator on adult consumers is driven to extinction, the predator on juvenile may follow suit when its mortality is high. In more complex food web configurations, in which juvenile and adult consumers forage on separate resources and each stage is vulnerable to predation by its own stage-specific predator, even mutual facilitation between two stage-specific predator species can occur (de Roos and Persson 2013, chapter 6). For specific combinations of productivity of the resources, on which juvenile and adult consumers forage, the two stage-specific predators can then only persist when together and will go extinct otherwise.

The positive relationship between consumer mortality and consumer density may in addition also lead to situations that stage-specific predators promote their own persistence. As shown in Online Resource 2 (Section II.5), if juveniles are more efficient in resource assimilation than adults and reproduction is hence more limited by resource than maturation, adult equilibrium density will increase with increasing adult mortality. High densities of a predator species that forages exclusively on adult consumers could in this case impose high adult predation mortality and potentially increase adult density above the density that these predators require for their own persistence. In contrast, if predator density is too low, the mortality they impose may not be sufficient to increase adult consumer density enough and predators die out. This may result in the occurrence of two alternative stable states for the community over a range of predator mortality rates: one resource-consumer equilibrium that is dominated by juvenile consumers and an alternative, resource-consumer-predator equilibrium, in which the consumer population for a substantial fraction consists of adults (de Roos and Persson 2002). The occurrence of these alternative stable states also entails that the predator population can exhibit a catastrophic collapse when its mortality increases and that the predator may not be able to recover from such a population collapse as the density of adult consumers in the consumer-resource equilibrium that follows the disappearance of the predator is too low for predators to achieve a positive growth rate. Since the predator in this case would only achieve a positive growth rate if it is itself present at sufficiently high density, this phenomenon is referred to as an emergent Allee effect (de Roos and Persson 2002; de Roos et al. 2003).

Summarising, the distinction between a juvenile and an adult phase and the energetic costs of covering basic maintenance requirements are two of the most fundamental elements of every life history. Models that take these two biological aspects into account make predictions about community structure and dynamics that are qualitatively different from the expectations generated by contemporary ecological theory based on unstructured population dynamic models. These contrasting predictions thereby challenge this unstructured ecological theory and its biological relevance.

### Electronic supplementary material

Below is the link to the electronic supplementary material.

(MW 596 KB)

(MW 4.56 MB)

(PDF 645 KB)

(PDF 1.82 MB)

## Appendix A: Stage-independent mortality and scaling with resource density in case of an arbitrary number of consumer stages

The result presented in the main text for a two-stage consumer- resource model that stage-independence of mortality and a qualitatively identical scaling of foraging, maturation and reproduction with resource density leads to a fading influence of population stage structure can be generalised to an arbitrary number of consumer stages. Consider a consumer population consisting of *n* stages with density *C*_*i*_, *i* = 1,…,*n*. Assume that all consumer stages forage on the resource at a rate *α*_*i*_*f*(*R*), where *f*(*R*) is an arbitrary, monotonously increasing function of the resource density *R*. Furthermore, assume that the stages with index *i* = *k*,…,*n* are adult and reproduce at a rate *β*_*i*_*g*(*R*). All stages *i* mature into the next stage at rate *γ*_*i*_*g*(*R*). To capture the lack of maturation out of the last stage (*i* = *n*), we simply set *γ*_*n*_ equal to 0. Finally, all consumers experience the same, stage-independent mortality rate *μ*.

The system of equations describing the consumer-resource dynamics is now given by the system of ODEs:

A.1 $$ \left\{\begin{array}{l} \displaystyle\frac{dR}{d t} = p(R) - \sum\limits_{i = 1}^{n} \alpha_{i} f(R) C_{i}\\[2ex] \displaystyle\frac{dC_{1}}{d t} = \sum\limits_{i=k}^{n} \beta_{i} g(R) C_{i} - \gamma_{1} g(R) C_{1} - \mu C_{1}\\[2ex] \displaystyle\frac{dC_{i}}{d t} = \gamma_{i-1} g(R) C_{i-1} - \gamma_{i} g(R) C_{i} - \mu C_{i}, \quad\qquad i = 2,\ldots,n \end{array}\right.  $$

where the function *p*(*R*) is the growth or production rate of the resource in the absence of consumers and *γ*_*n*_ = 0 by definition. Notice that model (A.1) can capture a broad range of individual life histories, including those in which the delay between birth and maturation is distributed following a gamma distribution (when all *γ*_*i*_ are identical, see Bjørnstad et al. 2016) with an expected juvenile delay depending on the resource density.

Now define the fraction of the total consumer population in stage *i* as *z*_*i*_:

$$z_{i} = \frac{C_{i}}{{\sum}_{i = 1}^{n} C_{i}} $$

Then,

$$\begin{array}{@{}rcl@{}} \frac{d z_{i}}{d t} &=& \frac{1}{{\sum}_{i = 1}^{n} C_{i}} \left( \frac{d C_{i}}{d t} - z_{i} \sum\limits_{i = 1}^{n} \frac{d C_{i}}{d t}\right)\\ &=& \frac{1}{{\sum}_{i = 1}^{n} C_{i}} \left( \frac{d C_{i}}{d t} - z_{i} \left( \sum\limits_{i=k}^{n} \beta_{i} g(R) C_{i} - \mu \sum\limits_{i = 1}^{n} C_{i} \right) \right)\\ &=& \frac{1}{{\sum}_{i = 1}^{n} C_{i}} \frac{d C_{i}}{d t} - z_{i} \sum\limits_{i=k}^{n} \beta_{i} g(R) z_{i} + \mu z_{i} \sum\limits_{i = 1}^{n} z_{i} \end{array} $$

Therefore,

A.2 $$ \frac{d z_{i}}{d t} = \frac{1}{{\sum}_{i = 1}^{n} C_{i}} \frac{d C_{i}}{d t} - \bar{\beta} g(R) z_{i} + \mu z_{i}  $$

in which the average fecundity parameter $\bar {\beta }$ is defined as follows:

A.3 $$ \bar{\beta} = \sum\limits_{i=k}^{n} \beta_{i} z_{i}  $$

For *i* = 1 we can derive from Eq. A.2:

$$\begin{array}{@{}rcl@{}} \frac{d z_{1}}{d t} &=& \frac{1}{{\sum}_{i = 1}^{n} C_{i}} \left( \sum\limits_{i=k}^{n} \beta_{i} g(R) C_{i} - \gamma_{1} g(R) C_{1} - \mu C_{1} \right) \\ && - \bar{\beta} g(R) z_{1} + \mu z_{1} \\ &=& \sum\limits_{i=k}^{n} \beta_{i} g(R) z_{i} - \gamma_{1} g(R) z_{1} - \mu z_{1} - \bar{\beta} g(R) z_{1} + \mu z_{1}\\ &=& \bar{\beta} g(R) - \gamma_{1} g(R) z_{1} - \bar{\beta} g(R) z_{1} \\ &=& (\bar{\beta} - (\gamma_{1} + \bar{\beta}) z_{1}) g(R) \end{array} $$

For *i* = 2,…,*n* we can derive from Eq. A.2:

$$\begin{array}{@{}rcl@{}} \frac{d z_{i}}{d t} &=& \frac{1}{{\sum}_{i = 1}^{n} C_{i}} (\gamma_{i-1} g(R) C_{i-1} - \gamma_{i} g(R) C_{i} - \mu C_{i})\\ && - \bar{\beta} g(R) z_{i} + \mu z_{i} \\ &=& \gamma_{i-1} g(R) z_{i-1} - \gamma_{i} g(R) z_{i} - \mu z_{i} - \bar{\beta} g(R) z_{i} + \mu z_{i} \\ &=& \gamma_{i-1} g(R) z_{i-1} - \gamma_{i} g(R) z_{i} - \bar{\beta} g(R) z_{i} \\ &=& (\gamma_{i-1} z_{i-1} - (\gamma_{i} + \bar{\beta}) z_{i}) g(R) \end{array} $$

The dynamics of the fractions *z*_*i*_ can hence be described by the following vector equation:

A.4 $$ \frac{d\mathbf{z}}{dt} = \frac{d}{dt} \left( \begin{array}{c} z_{1} \\ {\vdots} \\ z_{i} \\ {\vdots} \\ z_{n} \end{array}\right) = \left( \begin{array}{c} \bar{\beta} - \left( \gamma_{1} + \bar{\beta}\right) z_{1} \\ {\vdots} \\ \gamma_{i-1} z_{i-1} \,-\,\left( \gamma_{i} + \bar{\beta}\right) z_{i} \\ {\vdots} \\ \gamma_{n-1} z_{n-1} - \left( \gamma_{n} + \bar{\beta}\right) z_{n} \end{array}\right) g(R)  $$

(remember that *γ*_*n*_ = 0 by definition).

In the vector equation (A.4) the factor *g*(*R*) scales all the rates in the same way. This rate may hence vary over time, depending on the dynamics of *R*, but the factor *g*(*R*) only influences the rate of approach to an attractor, but not the final attractor of the vector **z**. In other words, the factor *g*(*R*) may scale the time axis in a particular way, but it will not influence the long-term fate of the fractions **z**.

The final attractor of **z** is determined by the equations:

A.5 $$ \left( \begin{array}{c} \bar{\beta} - (\gamma_{1} + \bar{\beta}) z_{1} \\ {\vdots} \\ \gamma_{i-1} z_{i-1} - (\gamma_{i} + \bar{\beta} ) z_{i} \\ {\vdots} \\ \gamma_{n-1} z_{n-1} - (\gamma_{n} + \bar{\beta}) z_{n} \end{array}\right) = 0 $$

which can be solved recursively to yield:

A.6 $$ \left\{\begin{array}{l} z_{1} = \frac{\bar{\beta}}{\gamma_{1} + \bar{\beta}}\\[4ex] z_{i} = \frac{\gamma_{i-1}}{\gamma_{i} + \bar{\beta}}\,z_{i-1}, \qquad\qquad i = 2,\ldots,n \end{array}\right. $$

or:

A.7 $$ \left\{\begin{array}{l} z_{1} = \displaystyle\frac{\bar{\beta}}{\gamma_{1} + \bar{\beta}}\\[2ex] z_{i} = \displaystyle\frac{\prod\limits_{j = 1}^{i-1}\gamma_{j}}{\prod\limits_{j = 2}^{i} (\gamma_{j} + \bar{\beta})}\,z_{1}, \qquad\qquad i = 2,\ldots,n \end{array}\right.  $$

For a given value of $\bar {\beta }$, these expressions for the fractions *z*_*i*_ of consumers in stage *i* obviously yield a unique, relative distribution over the stages. However, $\bar {\beta }$ is itself defined in terms of the fractions *z*_*i*_ (see Eq. A.3). Assuming that consumer individuals in the stage *i* = 1 do not reproduce (*k* > 1), the definition (A.3) for $\bar {\beta }$ can be combined with Eq. A.7 to yield:

$$\begin{array}{@{}rcl@{}} \bar{\beta} &=& \sum\limits_{i=k}^{n} \beta_{i} z_{i} = \sum\limits_{i=k}^{n} \beta_{i} \frac{\prod\limits_{j = 1}^{i-1}\gamma_{j}}{\prod\limits_{j = 2}^{i} (\gamma_{j} + \bar{\beta})}\,z_{1} \\ \Rightarrow\quad \bar{\beta} &=& \sum\limits_{i=k}^{n} \beta_{i} \frac{\prod\limits_{j = 1}^{i-1}\gamma_{j}}{\prod\limits_{j = 2}^{i} (\gamma_{j} + \bar{\beta})}\, \frac{\bar{\beta}}{\gamma_{1} + \bar{\beta}} \end{array} $$

$\bar {\beta }$ should therefore satisfy the following condition:

A.8 $$ \sum\limits_{i=k}^{n} \beta_{i} \frac{\prod\limits_{j = 1}^{i-1}\gamma_{j}}{\prod\limits_{j = 1}^{i} (\gamma_{j} + \bar{\beta})} = 1  $$

The left-hand side of Eq. A.8 is a strictly decreasing function of $\bar {\beta }$, which implies that there is a unique value of $\bar {\beta }$ satisfying this condition, as long as:

A.9 $$ \sum\limits_{i=k}^{n} \frac{\beta_{i}}{\gamma_{i}} > 1 $$

which should always be the case given that we assumed *γ*_*n*_ = 0.

The derivation above implies that the dynamical system (A.4) for **z** over time approaches a unique equilibrium that is determined by Eqs. A.7 and A.8, where the value of $\bar {\beta }$ is the unique solution of Eqs. A.8 and A.7 determines the unique, stable, relative stage distribution that is associated with this value of $\bar {\beta }$. Most importantly, this approach to the stable stage distribution decouples from and is hence unaffected by the dynamics of the resource *R* and the dynamics of the total number of consumers ${\sum }_{i = 1}^{n} C_{i}$, except for the fact that the dynamics of *R* determine the rate at which the stable stage distribution is approached.

Defining the total consumer population abundance *C* as follows:

A.10 $$ C = \sum\limits_{i = 1}^{n} C_{i} $$

and the average resource foraging parameter $\bar {\alpha }$ as follows:

A.11 $$ \bar{\alpha} = \sum\limits_{i = 1}^{n} \alpha_{i} z_{i}, $$

it can be concluded that the long-term dynamics of the consumer-resource model (A.1) is completely captured by the following *asymptotically autonomous system* (Thieme 1994):

A.12 $$ \left\{\begin{array}{l} \frac{dR}{d t} = g(R) - \bar{\alpha} f(R) C\\[2ex] \frac{dC}{d t} = \bar{\beta} g(R) C - \mu C \end{array}\right. $$

after the (decoupled) dynamics of the relative stage distribution of the consumer has settled down to its attractor determined by Eqs. A.7 and A.8.

## Appendix B: Different rates of juvenile and adult foraging, maturation and reproduction

All derivations discussed below have been carried out using Maple (version 18; Maplesoft, a division of Waterloo Maple Inc., Waterloo, Ontario). The Maple document with step-by-step calculations is provided as Online Resource 1.

The equilibrium state of the general model (2) is determined by the set of conditions (6) presented in the main text. These equations can be used to express the equilibrium adult consumer density in terms of the equilibrium density of juveniles:

$$\bar{C}_{\mathrm{A}} = \frac{g_{\mathrm{J}}(\bar{R})}{\mu_{\mathrm{A}}}\,\bar{C}_{\mathrm{J}} $$

Substitution of this equality in the right-hand side of $H_{2}(\bar {R},\bar {C}_{\mathrm {J}},\bar {C}_{\mathrm {A}})$ leads to the following condition determining the resource density in a non-trivial equilibrium, that is, an equilibrium with positive juvenile and adult consumer densities:

B.1 $$ g_{\mathrm{J}}(\bar{R}) (g_{\mathrm{A}}(\bar{R}) - \mu_{\mathrm{A}}) - \mu_{\mathrm{J}}\mu_{\mathrm{A}} = 0  $$

Since *g*_J_(*R*) and *g*_A_(*R*) are increasing functions of *R* the equilibrium condition (B.1) makes clear that for a given parameter set this non-trivial consumer-resource equilibrium is unique. Furthermore, condition (B.1) can only be satisfied if $g_{\mathrm {A}}(\bar {R})>\mu _{\mathrm {A}}$.

The equilibrium conditions $H_{1}(\bar {R},\bar {C}_{\mathrm {J}},\bar {C}_{\mathrm {A}})$ and $H_{3}(\bar {R},\bar {C}_{\mathrm {J}},\bar {C}_{\mathrm {A}})$ can also be used to derive expressions for the equilibrium densities of juvenile and adult consumers in terms of the equilibrium resource density $\bar {R}$:

B.2 $$\begin{array}{@{}rcl@{}} \bar{C}_{\mathrm{J}} &=& \frac{p(\bar{R}) \mu_{\mathrm{A}}}{f_{\mathrm{J}}(\bar{R})\mu_{\mathrm{A}} + f_{\mathrm{A}}(\bar{R})g_{\mathrm{J}}(\bar{R})} \end{array} $$

B.3 $$\begin{array}{@{}rcl@{}} \bar{C}_{\mathrm{A}} &=& \frac{p(\bar{R})g_{\mathrm{J}}(\bar{R})}{f_{\mathrm{J}}(\bar{R})\mu_{\mathrm{A}} + f_{\mathrm{A}}(\bar{R})g_{\mathrm{J}}(\bar{R})} \end{array} $$

Therefore, given a unique equilibrium resource density, the consumer stage distribution is also unique.

## B.1 Equilibrium changes with increasing mortality

To assess how an increase in juvenile mortality affects the equilibrium densities of resource, juvenile and adult consumers, the implicit function theorem can be applied to the system of equilibrium conditions (7). Taking the derivative of these conditions with respect to *μ*_J_ leads to the following:

$$\left( \begin{array}{ccc} \frac{\partial H_1}{\partial R} & \frac{\partial H_1}{\partial \bar{C}_{\mathrm{J}}} & \frac{\partial H_1}{\partial \bar{C}_{\mathrm{A}}}\\[2ex] \frac{\partial H_2}{\partial R} & \frac{\partial H_2}{\partial \bar{C}_{\mathrm{J}}} & \frac{\partial H_2}{\partial \bar{C}_{\mathrm{A}}}\\[2ex] \frac{\partial H_3}{\partial R} & \frac{\partial H_3}{\partial \bar{C}_{\mathrm{J}}} & \frac{\partial H_3}{\partial \bar{C}_{\mathrm{A}}} \end{array}\right) \left( \begin{array}{c} \frac{d\bar{R}}{d\mu_{\mathrm{J}}}\\[2ex] \frac{d\bar{C}_{\mathrm{J}}}{d\mu_{\mathrm{J}}}\\[2ex] \frac{d\bar{C}_{\mathrm{A}}}{d\mu_{\mathrm{J}}} \end{array}\right) + \left( \begin{array}{c} 0\\[2ex] - \bar{C}_{\mathrm{J}}\\[2ex] 0 \end{array}\right) = 0 $$

where the first term represents the indirect effects of *μ*_J_ on the equilibrium densities $\bar {R}$, $\bar {C}_{\mathrm {J}}$ and $\bar {C}_{\mathrm {A}}$ and the second term represents the direct effect of *μ*_J_ on the functions $H_{1}(\bar {R},\bar {C}_{\mathrm {J}},\bar {C}_{\mathrm {A}})$, $H_{2}(\bar {R},\bar {C}_{\mathrm {J}},\bar {C}_{\mathrm {A}})$ and $H_{3}(\bar {R},\bar {C}_{\mathrm {J}},\bar {C}_{\mathrm {A}})$. Notice that the matrix in the first term of the equation above is the Jacobian matrix of the model, evaluated at the non-trivial equilibrium densities:

B.4 $$ J \,=\, \left( \!\begin{array}{ccc} p^{\prime}(\bar{R}) \,-\, {f}_{\mathrm{J}}^{\prime}(\bar{R}) \bar{C}_{\mathrm{J}} \,-\, {f}_{\mathrm{A}}^{\prime}(\bar{R}) \bar{C}_{\mathrm{A}} & -f_{\mathrm{J}}(\bar{R}) & -\,f_{\mathrm{A}}(\bar{R})\\[2ex] {g}_{\mathrm{A}}^{\prime}(\bar{R}) \bar{C}_{\mathrm{A}} \,-\, {g}_{\mathrm{J}}^{\prime}(\bar{R}) \bar{C}_{\mathrm{J}} & -g_{\mathrm{J}}(\bar{R})-\mu_{\mathrm{J}} & g_{\mathrm{A}}(\bar{R})\\[2ex] {g}_{\mathrm{J}}^{\prime}(\bar{R}) \bar{C}_{\mathrm{J}} & g_{\mathrm{J}}(\bar{R}) & -\mu_{\mathrm{A}} \end{array}\!\right) $$

To determine the derivatives of the resource, juvenile and adult consumer density in equilibrium with respect to the parameters *μ*_J_, $d\bar {R}/d\mu _{\mathrm {J}}$, $d\bar {C}_{\mathrm {J}}/d\mu _{\mathrm {J}}$ and $d\bar {C}_{\mathrm {A}}/d\mu _{\mathrm {J}}$, respectively, the following (linear) system of equations has to be solved:

B.5 $$ J \left( \begin{array}{c} \frac{d\bar{R}}{d\mu_{\mathrm{J}}}\\[2ex] \frac{d\bar{C}_{\mathrm{J}}}{d\mu_{\mathrm{J}}}\\[2ex] \frac{d\bar{C}_{\mathrm{A}}}{d\mu_{\mathrm{J}}} \end{array}\right) = \left( \begin{array}{c} 0\\[2ex] \bar{C}_{\mathrm{J}}\\[2ex] 0\end{array}\right)  $$

where we are mostly interested in the sign of the derivatives $d\bar {C}_{\mathrm {J}}/d\mu _{\mathrm {J}}$ and $d\bar {C}_{\mathrm {A}}/d\mu _{\mathrm {J}}$.

The linear equation system (B.5) can be solved using Cramer’s rule. As a first step of applying Cramer’s rule, the determinant of the Jacobian matrix *J* is computed. Using the equilibrium resource condition (B.1) for simplification (Online Resource 1, Eqs. 2.3–2.6), the determinant can be expressed as follows:

B.6 $$\begin{array}{@{}rcl@{}} D &=& -(f_{\mathrm{J}}(\bar{R}) (g_{\mathrm{A}}(\bar{R}) - \mu_{\mathrm{A}}) + f_{\mathrm{A}}(\bar{R})\mu_{\mathrm{J}})\, {g}_{\mathrm{J}}^{\prime}(\bar{R})\,\bar{C}_{\mathrm{J}}\\ && -\, (f_{\mathrm{J}}(\bar{R})\mu_{\mathrm{A}} + f_{\mathrm{A}}(\bar{R})g_{\mathrm{J}}(\bar{R}))\, {g}_{\mathrm{A}}^{\prime}(\bar{R})\,\bar{C}_{\mathrm{A}} \end{array} $$

Since it is assumed that either ${g}_{\mathrm {J}}^{\prime }(\bar {R})>0$ or ${g}_{\mathrm {A}}^{\prime }(\bar {R})>0$, it can be inferred that *D* is strictly negative.

Using the expression for the determinant of the Jacobian matrix *D*, the derivative $d\bar {C}_{\mathrm {J}}/d\mu _{\mathrm {J}}$ is given by (Online Resource 1, Eq. 2.10):

B.7 $$\begin{array}{@{}rcl@{}} \frac{d\bar{C}_{\mathrm{J}}}{d\mu_{\mathrm{J}}} \!&=&\! D^{-1} \left|\begin{array}{ccc} p^{\prime}(\bar{R}) - {f}_{\mathrm{J}}^{\prime} (\bar{R}) \bar{C}_{\mathrm{J}} - {f}_{\mathrm{A}}^{\prime} (\bar{R}) \bar{C}_{\mathrm{A}} & 0 & -\,f_{\mathrm{A}}(\bar{R})\\ {g}_{\mathrm{A}}^{\prime}(\bar{R}) \bar{C}_{\mathrm{A}} - {g}_{\mathrm{J}}^{\prime}(\bar{R}) \bar{C}_{\mathrm{J}} & \bar{C}_{\mathrm{J}} & g_{\mathrm{A}}(\bar{R})\\ {g}_{\mathrm{J}}^{\prime}(\bar{R}) \bar{C}_{\mathrm{J}} & 0 & -\mu_{A} \end{array}\right|\!\\ \!&=&\! D^{-1} ((-p^{\prime}(\bar{R}) + {f}_{\mathrm{J}}^{\prime}(\bar{R}) \bar{C}_{\mathrm{J}} + {f}_{\mathrm{A}}^{\prime}(\bar{R}) \bar{C}_{\mathrm{A}})\mu_{\mathrm{A}}\\ && + f_{\mathrm{A}}(\bar{R}) {g}_{\mathrm{J}}^{\prime}(\bar{R})\bar{C}_{\mathrm{J}})\bar{C}_{\mathrm{J} }\end{array} $$

Given the assumptions on *p*(*R*), *f*_J_(*R*), *f*_A_(*R*) and *g*_J_(R), the above expression makes clear that $d\bar {C}_{\mathrm {J}}/d\mu _{\mathrm {J}}$ always has the same sign as *D* and hence is *negative*. Thus, juvenile consumer density always decreases with increasing juvenile mortality.

The derivative $d\bar {C}_{\mathrm {A}}/d\mu _{\mathrm {J}}$ is given by (Online Resource 1, Eqs. 2.12–2.15):

B.8 $$\begin{array}{@{}rcl@{}} \frac{d\bar{C}_{\mathrm{A}}}{d\mu_{\mathrm{J}}} \!&=&\! D^{-1} \!\left| \!\begin{array}{ccc} p^{\prime}(\bar{R})-{f}_{\mathrm{J}}^{\prime}(\bar{R}) \bar{C}_{\mathrm{J}}-{f}_{\mathrm{A}}^{\prime}(\bar{R}) \bar{C}_{\mathrm{A}} & -f_{\mathrm{J}}(\bar{R}) & 0\\ {g}_{\mathrm{A}}^{\prime}(\bar{R}) \bar{C}_{\mathrm{A}}-{g}_{\mathrm{J}}^{\prime}(\bar{R}) \bar{C}_{\mathrm{J}} & -g_{\mathrm{J}}(\bar{R})-\mu_{\mathrm{J}} & \bar{C}_{\mathrm{J}}\\ {g}_{\mathrm{J}}^{\prime}(\bar{R}) \bar{C}_{\mathrm{J}} & g_{\mathrm{J}}(\bar{R}) & 0 \end{array}\!\right|\\ \!&=&\! D^{-1} \!\left( \! -p^{\prime}(\bar{R}) \,+\, {f}_{\mathrm{A}}^{\prime}(\bar{R}) \bar{C}_{\mathrm{A}} \,+\, \left( \frac{f_{\mathrm{J}}(\bar{R})}{g_{\mathrm{J}}(\bar{R})}\right)^{\prime}\! g_{\mathrm{J}}(\bar{R})\bar{C}_{\mathrm{J}} \!\right)\! g_{\mathrm{J}}(\bar{R})\bar{C}_{\mathrm{J}}\\ \end{array} $$

which also always has the same sign as *D* and hence is *negative*, provided that the derivative of the quotient function *f*_J_(*R*)/*g*_J_(*R*) is non-negative:

B.9 $$ \left( \frac{f_{\mathrm{J}}(\bar{R})}{g_{\mathrm{J}}(\bar{R})}\right)^{\prime} \geq 0  $$

Thus, as long as this inequality holds, adult consumer density also always decreases with increasing juvenile mortality, while adult consumer density can potentially increase with increasing juvenile mortality if the derivative in the left-hand side of inequality (B.9) is negative.

In a similar manner, to compute the derivatives of the resource, juvenile and adult consumer densities in equilibrium with respect to the adult consumer mortality *μ*_*A*_, consider the system of equilibrium conditions (8). Taking the derivative of these conditions with respect to *μ*_A_ leads to the following (linear) system of equations that can be solved for $d\bar {R}/d\mu _{\mathrm {A}}$, $d\bar {C}_{\mathrm {J}}/d\mu _{\mathrm {A}}$ and $d\bar {C}_{\mathrm {A}}/d\mu _{\mathrm {A}}$:

B.10 $$ J \left( \begin{array}{c} \frac{d\bar{R}}{d\mu_{\mathrm{A}}}\\[2ex] \frac{d\bar{C}_{\mathrm{J}}}{d\mu_{\mathrm{A}}}\\[2ex] \frac{d\bar{C}_{\mathrm{A}}}{d\mu_{\mathrm{A}}} \end{array}\right) = \left( \begin{array}{c} 0\\[2ex] 0\\[2ex] \bar{C}_{\mathrm{A}} \end{array}\right)  $$

Again using the expression for the determinant of the Jacobian matrix *D*, the derivative $d\bar {C}_{\mathrm {J}}/d\mu _{\mathrm {A}}$ is given by (Online Resource 1, Eqs. 2.18–2.19):

B.11 $$\begin{array}{@{}rcl@{}} \frac{d\bar{C}_{\mathrm{J}}}{d\mu_{\mathrm{A}}} \!&=&\! D^{-1} \left|\begin{array}{ccc} p^{\prime}(\bar{R}) \,-\, {f}_{\mathrm{J}}^{\prime}(\bar{R}) \bar{C}_{\mathrm{J}} \,-\, {f}_{\mathrm{A}}^{\prime}(\bar{R}) \bar{C}_{\mathrm{A}} & 0 & -\,f_{\mathrm{A}}(\bar{R})\\ {g}_{\mathrm{A}}^{\prime}(\bar{R}) \bar{C}_{\mathrm{A}} \,-\, {g}_{\mathrm{J}}^{\prime}(\bar{R}) \bar{C}_{\mathrm{J}} & 0 & g_{\mathrm{A}}(\bar{R})\\ {g}_{\mathrm{J}}^{\prime}(\bar{R}) \bar{C}_{\mathrm{J}} & \bar{C}_{\mathrm{A}} & -\mu_{\mathrm{A}} \end{array}\!\right|\\ &=&\! D^{-1} \!\left( \vphantom{\left( \frac{f_{A}(\bar{R})}{g_{A}(\bar{R})}\right)^{\prime}}\! g_{\mathrm{A}}(\bar{R}) (-p^{\prime}(\bar{R}) \,+\, {f}_{\mathrm{J}}^{\prime}(\bar{R})\bar{C}_{\mathrm{J}}) \,+\, f_{\mathrm{A}}(\bar{R}){g}_{\mathrm{J}}^{\prime}(\bar{R})\bar{C}_{\mathrm{J}}\right.\\ &&\left. + \left( \frac{f_{\mathrm{A}}(\bar{R})}{g_{\mathrm{A}}(\bar{R})}\right)^{\prime}\! (g_{\mathrm{A}}(\bar{R}))^{2}\bar{C}_{\mathrm{A}}\!\right)\! \bar{C}_{\mathrm{A}} \end{array} $$

which also always has the same sign as *D* and hence is *negative*, provided that the derivative of the quotient function *f*_A_(*R*)/*g*_A_(*R*) is non-negative:

B.12 $$ \left( \frac{f_{\mathrm{A}}(\bar{R})}{g_{\mathrm{A}}(\bar{R})}\right)^{\prime} \geq 0  $$

Thus, as long as this inequality holds, juvenile consumer density always decreases with increasing adult mortality, while juvenile consumer density can potentially increase with increasing adult mortality if the derivative in the left-hand side of inequality (B.12) is negative.

Finally, the derivative $d\bar {C}_{\mathrm {A}}/d\mu _{\mathrm {A}}$ is given by (Online Resource 1, Eqs. 2.21–2.22):

B.13 $$\begin{array}{@{}rcl@{}} \frac{d\bar{C}_{\mathrm{A}}}{d\mu_{\mathrm{A}}} \!&=&\! D^{-1}\! \left|\!\begin{array}{ccc} p^{\prime}(\bar{R}) \,-\, {f}_{\mathrm{J}}^{\prime}(\bar{R}) \bar{C}_{\mathrm{J}} \,-\, {f}_{\mathrm{A}}^{\prime}(\bar{R}) \bar{C}_{\mathrm{A}} & -f_{\mathrm{J}}(\bar{R}) & 0\\ {g}_{\mathrm{A}}^{\prime}(\bar{R}) \bar{C}_{\mathrm{A}} \,-\, {g}_{\mathrm{J}}^{\prime}(\bar{R}) \bar{C}_{\mathrm{J}} & -g_{\mathrm{J}}(\bar{R}) \,-\, \mu_{J} & 0\\ {g}_{\mathrm{J}}^{\prime}(\bar{R}) \bar{C}_{\mathrm{J}} & g_{\mathrm{J}}(\bar{R}) & \bar{C}_{\mathrm{A}} \end{array}\right|\\ &=&\! D^{-1} \left( \vphantom{\left( \frac{f_{\mathrm{J}}(\bar{R})}{g_{\mathrm{J}}(\bar{R})}\right)^{\!\prime}}(g_{\mathrm{J}}(\bar{R}) \,+\, \mu_{\mathrm{J}}) (-p^{\prime}(\bar{R}) \,+\, {f}_{\mathrm{A}}^{\prime}(\bar{R})\bar{C}_{\mathrm{A}}) \,+\, \mu_{\mathrm{J}} {f}_{\mathrm{J}}^{\prime}(\bar{R}) \bar{C}_{\mathrm{J}}\right.\\ && \left. +\, f_{\mathrm{J}}(\bar{R})g_{\mathrm{A}}^{\prime}(\bar{R})\bar{C}_{\mathrm{A}} \,+\, \left( \frac{f_{\mathrm{J}}(\bar{R})}{g_{\mathrm{J}}(\bar{R})}\right)^{\!\prime} \! (g_{\mathrm{J}}(\bar{R}))^{2}\bar{C}_{\mathrm{J}}\!\right)\!\bar{C}_{\mathrm{A}} \end{array} $$

which also has the same sign as *D* and hence is *negative*, provided that the derivative of the quotient function *f*_J_(*R*)/*g*_J_ (*R*) is non-negative (inequality (B.9)). Thus, as long as inequality (B.9) holds, adult consumer density always decreases with increasing adult mortality, while adult consumer density can potentially increase with increasing adult mortality if the derivative in the left-hand side of inequality (B.9) is negative.

In summary, the analysis in this section shows that any increase in (stage-specific) mortality will decrease the equilibrium densities of juvenile and adult consumers, provided the derivatives of the quotient functions *f*_J_(*R*)/*g*_J_(*R*) and *f*_A_(*R*)/*g*_A_(*R*) are non-negative (inequalities (B.9) and (B.12)). If the inequality

B.14 $$ \left( \frac{f_{\mathrm{J}}(\bar{R})}{g_{\mathrm{J}}(\bar{R})}\right)^{\prime} < 0  $$

would hold, the juvenile maturation rate $g_{\mathrm {J}}(\bar {R})$ at equilibrium increases faster with an increase in resource density than the juvenile foraging rate $f_{\mathrm {J}}(\bar {R})$ at equilibrium. This implies that the increase in resource density translates into an increased efficiency with which juveniles use acquired resources for maturation. In this case, adult consumer density at equilibrium can potentially increase with an increase in either juvenile or adult mortality (or both).

If the inequality

B.15 $$ \left( \frac{f_{\mathrm{A}}(\bar{R})}{g_{\mathrm{A}}(\bar{R})}\right)^{\prime} < 0  $$

would hold, the adult reproduction rate $g_{\mathrm {A}}(\bar {R})$ at equilibrium increases faster with an increase in resource density than the adult foraging rate $f_{\mathrm {A}}(\bar {R})$ at equilibrium. This implies that the increase in resource density translates into an increased efficiency with which adults use acquired resources for reproduction. In this case, juvenile consumer density at equilibrium can potentially increase with an increase in adult mortality.

## B.2 Stability of the equilibrium

The characteristic equation determining the stability of the non-trivial equilibrium is determined by the following characteristic polynomial in terms of the eigenvalue *λ*:

B.16 $$ \lambda^{3} + a_{1}\lambda^{2} + a_{2}\lambda + a_{3} = 0 $$

in which the coefficients *a*_1_, *a*_2_, and *a*_3_ are given by the following:

B.17 $$\begin{array}{@{}rcl@{}} a_{1} &=& -p^{\prime}(\bar{R}) + {f}_{\mathrm{J}}^{\prime}(\bar{R}) \bar{C}_{\mathrm{J}} + {f}_{\mathrm{A}}^{\prime} (\bar{R}) \bar{C}_{\mathrm{A}} + g_{\mathrm{J}}(\bar{R}) + \mu_{\mathrm{J}} + \mu_{\mathrm{A}}\\ a_{2} &=& (g_{\mathrm{J}}(\bar{R})+\mu_{\mathrm{J}}+\mu_{\mathrm{A}}) (-p^{\prime}(\bar{R}) + {f}_{\mathrm{A}}^{\prime}(\bar{R}) \bar{C}_{\mathrm{A}})\\ && + (\mu_{\mathrm{J}}+\mu_{\mathrm{A}}) {f}_{\mathrm{J}}^{\prime}(\bar{R}) \bar{C}_{\mathrm{J}}\\ && + f_{\mathrm{A}}(\bar{R}) {g}_{\mathrm{J}}^{\prime}(\bar{R}) \bar{C}_{\mathrm{J}} + f_{\mathrm{J}}(\bar{R}) {g}_{\mathrm{A}}^{\prime}(\bar{R}) \bar{C}_{\mathrm{A}}\\ && + \left( \frac{f_{\mathrm{J}}(\bar{R})}{g_{\mathrm{J}}(\bar{R})}\right)^{\prime} (g_{\mathrm{J}}(\bar{R}))^{2} \bar{C}_{\mathrm{J}} \end{array} $$

$$\begin{array}{@{}rcl@{}} a_{3} &=& ((g_{\mathrm{A}}(\bar{R}) - \mu_{\mathrm{A}})f_{\mathrm{J}}(\bar{R}) + \mu_{\mathrm{J}} f_{\mathrm{A}}(\bar{R})) {g}_{\mathrm{J}}^{\prime}(\bar{R}) \bar{C}_{\mathrm{J}}\\ && + (f_{\mathrm{A}}(\bar{R})g_{\mathrm{J}}(\bar{R}) + f_{\mathrm{J}}(\bar{R})\mu_{\mathrm{A}}) {g}_{\mathrm{A}}^{\prime}(\bar{R}) \bar{C}_{\mathrm{A}} \end{array} $$

(see Online Resource 1, Eqs. 3.2–3.7).

For systems of 3 ODEs the Routh-Hurwitz conditions stipulate that the equilibrium is stable if *a*_1_ > 0, *a*_3_ > 0 and *a*_1_*a*_2_ − *a*_3_ > 0. Given that $p^{\prime }(R) \leq 0$, ${f}_{J}^{\prime }(R) \geq 0$, ${f}_{\mathrm {A}}^{\prime }(R) \geq 0$, ${g}_{\mathrm {J}}^{\prime }(R) \geq 0$, ${g}_{\mathrm {A}}^{\prime }(R) \geq 0$, and necessarily $g_{\mathrm {A}}(\bar {R}) > \mu _{\mathrm {A}}$ (see Eq. B.1), it is clear that the conditions *a*_1_ > 0 and *a*_3_ > 0 are satisfied. The detailed calculations in Online Resource 1 (see Eqs. 3.9–3.19) show that the expression for *a*_1_*a*_2_ − *a*_3_ is also positive indicating that the equilibrium is stable, provided that the derivatives of the quotient functions *f*_J_(*R*)/*g*_J_(*R*) and *f*_A_(*R*)/*g*_A_(*R*) are non-negative (inequalities (B.9) and (B.12)).

Population cycles can hence only occur if the efficiency with which juveniles can use acquired resources for maturation increases with an increase in resource density, i.e. the juvenile maturation rate *g*_J_(*R*) increases faster with an increase in resource density than the juvenile foraging rate *f*_J_(*R*) and inequality (B.14) holds, or in case the efficiency with which adults can use acquired resources for reproduction increases with resource density, i.e. the adult reproduction rate *g*_A_(*R*) increases faster with an increase in resource density than the adult foraging rate *f*_A_(*R*) and inequality (B.15) holds.

## Appendix C: Consumer maturation and reproduction reduced by somatic maintenance costs

All derivations discussed below have been carried out using Maple (version 18; Maplesoft, a division of Waterloo Maple Inc., Waterloo, Ontario). The Maple document with step-by-step calculations is provided as Online Resource 2.

If resource productivity is constant *p*(*R*) = *P*, juveniles and adults forage with exactly the same rate, *f*_J_(*R*) = *f*_A_(*R*) := *f*(*R*), experience the same mortality, *μ*_J_ = *μ*_A_ := *μ*, and similar maintenance costs *T*, but potentially differ in their efficiency to assimilate the resource, reproduction and maturation rate are given by *g*_A_(*R*) := (*β**f*(*R*) − *T*)^+^ and *g*_J_(*R*) := (*γ**f*(*R*) − *T*)^+^, respectively. Under these assumptions the equilibrium of model (13) is determined by the following set of conditions:

C.1 $$ \left\{\!\begin{array}{ll} H_{1}(\bar{R},\bar{\bar{C}}_{\mathrm{J}},\bar{C}_{\mathrm{A}})\,=\,P-f(\bar{R}) (\bar{C}_{\mathrm{J}}+\bar{C}_{\mathrm{A}})&= 0\\[1ex] H_{2}(\bar{R},\bar{C}_{\mathrm{J}},\bar{C}_{\mathrm{A}})\,=\,(\beta f(\bar{R})\,-\,T) \bar{C}_{\mathrm{A}}-(\gamma f(\bar{R})-T) \bar{C}_{\mathrm{J}}-\mu \bar{C}_{\mathrm{J}}\!\!&= 0\\[1ex] H_{3}(\bar{R},\bar{C}_{\mathrm{J}},\bar{C}_{\mathrm{A}})\,=\,(\gamma f(\bar{R})\,-\,T)\bar{C}_{\mathrm{J}}-\mu \bar{C}_{\mathrm{A}}&= 0 \end{array}\right.  $$

in which it is assumed that in equilibrium necessarily $\beta f(\bar {R})>T$ and $\gamma f(\bar {R})>T$ and hence the superscripts ‘ + ’ in the reproduction and maturation rate *g*_A_(*R*) and *g*_J_(*R*) have been dropped from the equations. These equations can be used to express the equilibrium adult consumer density in terms of the equilibrium density of juveniles:

$$\bar{C}_{\mathrm{A}} = \frac{(\gamma f(\bar{R})-T)}{\mu}\,\bar{C}_{\mathrm{J}} $$

Substitution of this equality in the right-hand side of $H_{2}(\bar {R},\bar {C}_{\mathrm {J}},\bar {C}_{\mathrm {A}})$ leads to the following condition determining the resource density in a non-trivial equilibrium, that is, an equilibrium with positive juvenile and adult consumer densities:

C.2 $$ (\gamma f(\bar{R})-T) (\beta f(\bar{R}) - T - \mu) - \mu^{2} = 0  $$

Since *f*(*R*) is an increasing function of *R* and $\gamma f(\bar {R})>T$ the equilibrium condition can only be satisfied if $\beta f(\bar {R})>T+\mu $, which also implies that the equilibrium condition (C.2) determines a unique, positive value of the resource density in equilibrium. The equilibrium conditions $H_{1}(\bar {R},\bar {C}_{\mathrm {J}},\bar {C}_{\mathrm {A}})$ and $H_{3}(\bar {R},\bar {C}_{\mathrm {J}},\bar {C}_{\mathrm {A}})$ can furthermore be used to derive expressions for the equilibrium densities of juvenile and adult consumers in terms of the equilibrium resource density $\bar {R}$:

C.3 $$\begin{array}{@{}rcl@{}} \bar{C}_{\mathrm{J}} &=& \frac{\mu}{f(\bar{R}) (\gamma f(\bar{R})-T+\mu)}\,P \end{array} $$

C.4 $$\begin{array}{@{}rcl@{}} \bar{C}_{\mathrm{A}} &=& \frac{(\gamma f(\bar{R})-T)}{f(\bar{R}) (\gamma f(\bar{R})-T+\mu)}\,P \end{array} $$

Therefore, given a unique equilibrium resource density, the consumer stage distribution is also unique.

The equilibrium condition can be rewritten in terms of three scaled quantities $h(\bar {R}):=\gamma f(\bar {R})/T$, *q* := *β*/*γ* and *m* := *μ*/*T* as follows:

C.5 $$ (h(\bar{R})-1) (q\, h(\bar{R})-1-m) - m^{2} = 0  $$

which leads to the unique, positive solution for $h(\bar {R})=\gamma f(\bar {R})/T >1$ (Online Resource 2, Eq. 1.12):

C.6 $$ h(\bar{R})=\frac{m+q + 1+\sqrt{(m+q + 1)^{2}-4\,q\,(1+m-m^{2})}}{2q}  $$

## C.1 Equilibrium changes with increasing mortality

To assess how an increase in mortality affects the equilibrium densities of resource, juvenile and adult consumers, the implicit function theorem can be applied to the system of equilibrium conditions (C.1). This system of equations can be rewritten as

$$\left\{\begin{array}{l} H_1(\bar{R}(\mu),\bar{C}_{\mathrm{J}}(\mu),\bar{C}_{\mathrm{A}}(\mu),\mu) = 0\\[0.5ex] H_2(\bar{R}(\mu),\bar{C}_{\mathrm{J}}(\mu),\bar{C}_{\mathrm{A}}(\mu),\mu) = 0\\[0.5ex] H_3(\bar{R}(\mu),\bar{C}_{\mathrm{J}}(\mu),\bar{C}_{\mathrm{A}}(\mu),\mu) = 0 \end{array}\right. $$

which emphasises the fact that the equilibrium densities $\bar {R}$, $\bar {C}_{\mathrm {J}}$ and $\bar {C}_{\mathrm {A}}$ depend indirectly on the mortality rate *μ* through the dependence of the functions $H_{1}(\bar {R},\bar {C}_{\mathrm {J}},\bar {C}_{\mathrm {A}})$, $H_{2}(\bar {R},\bar {C}_{\mathrm {J}},\bar {C}_{\mathrm {A}})$ and $H_{3}(\bar {R},\bar {C}_{\mathrm {J}},\bar {C}_{\mathrm {A}})$ on *μ*. Taking the derivative of the conditions above with respect to *μ* leads to the following:

$$\left( \begin{array}{ccc} \frac{\partial H_1}{\partial R} & \frac{\partial H_1}{\partial \bar{C}_{\mathrm{J}}} & \frac{\partial H_1}{\partial \bar{C}_{\mathrm{A}}}\\[2ex] \frac{\partial H_2}{\partial R} & \frac{\partial H_2}{\partial \bar{C}_{\mathrm{J}}} & \frac{\partial H_2}{\partial \bar{C}_{\mathrm{A}}}\\[2ex] \frac{\partial H_3}{\partial R} & \frac{\partial H_3}{\partial \bar{C}_{\mathrm{J}}} & \frac{\partial H_3}{\partial \bar{C}_{\mathrm{A}}} \end{array}\right) \left( \begin{array}{c} \frac{d\bar{R}}{d\mu}\\[2ex] \frac{d\bar{C}_{\mathrm{J}}}{d\mu}\\[2ex] \frac{d\bar{C}_{\mathrm{A}}}{d\mu} \end{array}\right) + \left( \begin{array}{c} 0\\[2ex] -\bar{C}_{\mathrm{J}}\\[2ex] -\bar{C}_{\mathrm{A}} \end{array}\right) = 0 $$

where the first term represents the indirect effect of *μ* on the equilibrium densities $\bar {R}$, $\bar {C}_{\mathrm {J}}$ and $\bar {C}_{\mathrm {A}}$ and the second term represents the direct effect of *μ* on the functions $H_{1}(\bar {R},\bar {C}_{\mathrm {J}},\bar {C}_{\mathrm {A}})$, $H_{2}(\bar {R},\bar {C}_{J},\bar {C}_{\mathrm {A}})$ and $H_{3}(\bar {R},\bar {C}_{\mathrm {J}},\bar {C}_{A})$. Notice that the matrix in the first term of the equation above is the Jacobian matrix of the model, evaluated at the non-trivial equilibrium densities (Online Resource 2, Eq. 2.2):

C.7 $$ J \,=\, \left( \!\begin{array}{ccc} -f^{\prime}(\bar{R}) (\bar{C}_{\mathrm{J}}+ \bar{C}_{\mathrm{A}} ) & -f(\bar{R}) & -\,f(\bar{R})\\[2ex] \beta f^{\prime}(\bar{R}) \bar{C}_{\mathrm{A}}-\gamma f^{\prime}(\bar{R}) \bar{C}_{\mathrm{J}} & - (\gamma f(\bar{R})-T)-\mu & (\beta f(\bar{R})-T)\\[2ex] \gamma f^{\prime}(\bar{R}) \bar{C}_{\mathrm{J}} & (\gamma f(\bar{R})-T) & -\mu \end{array}\!\!\right) $$

To determine the derivatives of the resource, juvenile and adult consumer densities in equilibrium with respect to the parameter *μ*, $d\bar {R}/d\mu $, $d\bar {C}_{\mathrm {J}}/d\mu $ and $d\bar {C}_{\mathrm {A}}/d\mu $, respectively, the following (linear) system of equations has to be solved:

C.8 $$ J \left( \begin{array}{c} \frac{d\bar{R}}{d\mu}\\[2ex] \frac{d\bar{C}_{\mathrm{J}}}{d\mu}\\[2ex] \frac{d\bar{C}_{\mathrm{A}}}{d\mu} \end{array}\right) = \left( \begin{array}{c} 0\\[2ex] \bar{C}_{J}\\[2ex] \bar{C}_{A} \end{array}\right)  $$

where we are mostly interested in the sign of the derivatives $d\bar {C}_{\mathrm {J}}/d\mu $ and $d\bar {C}_{\mathrm {A}}/d\mu $.

The linear equation system (C.8) can be solved using Cramer’s rule. As a first step of applying Cramer’s rule, the determinant of the Jacobian matrix *J* is computed. Using the equilibrium resource condition (C.2) for simplification (Online Resource 2, Eqs. 2.3–2.4), the determinant can be expressed as follows:

C.9 $$\begin{array}{@{}rcl@{}} D &=& - ((\mu(\gamma f(\bar{R})-T+\mu)+T(\beta f(\bar{R})-T))\bar{C}_{\mathrm{J}}\\ &&+ (T(\gamma f(\bar{R})-T) + \mu(\gamma f(\bar{R})\\ && +\beta f(\bar{R})-T+\mu))\bar{C}_{\mathrm{A}})\,f^{\prime}(\bar{R}) \end{array} $$

Since $f^{\prime }(\bar {R})>0$, $\beta f(\bar {R})>T$ and $\gamma f(\bar {R})>T$, *D* is strictly negative.

Using the expression for the determinant, the derivative $d\bar {C}_{\mathrm {J}}/d\mu $ can be expressed as follows (Online Resource 2, Eqs. 2.7–2.10):

C.10 $$\begin{array}{@{}rcl@{}} \frac{d\bar{C}_{\mathrm{J}}}{d\mu} &=& D^{-1} ((\gamma f(\bar{R})+\mu) {\bar{C}}_{\mathrm{J}}^{2} + (\gamma f(\bar{R})+\beta f(\bar{R})\\ && - T+\mu)\bar{C}_{\mathrm{J}}\bar{C}_{\mathrm{A}} - T {\bar{C}}_{\mathrm{A}}^{2})f^{\prime}(\bar{R}) \end{array} $$

which indicates that juvenile equilibrium density increases with mortality if:

C.11 $$ (\gamma f(\bar{R})+\mu){\bar{C}}_{\mathrm{J}}^{2} + (\gamma f(\bar{R})+\beta f(\bar{R})-T+\mu)\bar{C}_{\mathrm{J}}\bar{C}_{\mathrm{A}} -T {\bar{C}}_{\mathrm{A}}^{2} < 0 $$

This inequality can be simplified (Online Resource 2, Eqs. 2.12–2.18) using the expressions (C.3) and (C.4) for $\bar {C}_{\mathrm {J}}$ and $\bar {C}_{\mathrm {A}}$ to the inequality:

C.12 $$ \frac{\beta f(\bar{R})-T}{\gamma f(\bar{R})-T} < \frac{T}{\gamma f(\bar{R})+ 2\mu} $$

Because in equilibrium $\gamma f(\bar {R})>T$ the right-hand side of this inequality is always smaller than 1, which implies that the condition can only be satisfied if *β* < *γ*. Furthermore, like the equilibrium condition (C.5) the inequality can be expressed in terms of three scaled quantities $h(\bar {R}):=\gamma f(\bar {R})/T$, *q* := *β*/*γ* and *m* := *μ*/*T*:

C.13 $$ \frac{q\,h(\bar{R})-1}{h(\bar{R})-1} < \frac{1}{h(\bar{R})+ 2m}  $$

Substitution of the equilibrium value $h(\bar {R})$ (C.6) into the inequality above, allows Maple to rewrite it in the form *q* < *L*_J_(*m*), where *L*_J_(*m*) is the following explicit function of *m* (Online Resource 2, Eqs. 2.20–2.25):

C.14 $$\begin{array}{@{}rcl@{}} L_{\mathrm{J}}(m) &=& \frac{-m^{4}-2m^{3}+m^{2}-4m-2}{8m^{4}-4m^{3}-4m^{2}-4m-2}\\ && + \frac{\sqrt{m^{8}+ 4m^{7}-46m^{6}+ 60m^{5}+ 45m^{4}}}{8m^{4}-4m^{3}-4m^{2}-4m-2} \end{array} $$

The function *L*_J_(*m*) constitutes the boundary of the parameter domain with juvenile density overcompensation shown in Fig. 3 (left panel).

Using the expressions for the determinant, the derivative $d\bar {C}_{\mathrm {A}}/d\mu $ can be expressed as (Online Resource 2, Eq. 2.27):

C.15 $$\begin{array}{@{}rcl@{}} \frac{d\bar{C}_{\mathrm{A}}}{d\mu} &=& D^{-1} ((\gamma f(\bar{R})+\beta f(\bar{R})-T+\mu){\bar{C}}_{\mathrm{A}}^{2}\\ && + (\gamma f(\bar{R})-2T+\mu) \bar{C}_{\mathrm{J}}\bar{C}_{\mathrm{A}} - T{\bar{C}}_{\mathrm{J}}^{2})f^{\prime}(\bar{R}) \end{array} $$

which indicates that adult equilibrium density increases with mortality if:

C.16 $$ (\gamma f(\bar{R})+\beta f(\bar{R})-T+\mu){\bar{C}}_{\mathrm{A}}^{2} + (\gamma f(\bar{R})-2T+\mu) \bar{C}_{\mathrm{J}}\bar{C}_{\mathrm{A}} - T{\bar{C}}_{\mathrm{J}}^{2} \!<\! 0 $$

This inequality can be simplified (Online Resource 2, Eqs. 2.29–2.37) using the expressions (C.3) and (C.4) for $\bar {C}_{\mathrm {J}}$ and $\bar {C}_{\mathrm {A}}$ to the inequality:

C.17 $$ \frac{\beta f(\bar{R})-T}{\gamma f(\bar{R})-T} > \frac{\gamma f(\bar{R})+\mu}{3T-2\gamma f(\bar{R})} $$

where it is required (see Online Resource 2, Eq. 2.36) that $T<\gamma f(\bar {R})<\tfrac {3}{2}T$. Given that the right-hand side of the inequality above is always larger than 1 for $T<\gamma f(\bar {R})<\frac {3}{2}T$, it can be concluded that an increase in adult density with mortality can only occur for *β* > *γ*.

The inequality can be expressed in terms of three scaled quantities $h(\bar {R}):=\gamma f(\bar {R})/T$, *q* := *β*/*γ* and *m* := *μ*/*T* as follows (Online Resource 2, Eq. 2.39):

C.18 $$ \frac{q\,h(\bar{R})-1}{h(\bar{R})-1} > \frac{h(\bar{R})+m}{3-2h(\bar{R})}  $$

with the restriction that $1<h(\bar {R})<\frac {3}{2}$.

Solving of the equilibrium condition for $h(\bar {R})$ (C.5) and the condition for the occurrence of adult overcompensation (C.18) for $h(\bar {R})$ and *q*, results in an inequality *q* > *L*_A_(*m*) (Online Resource 2, Eqs. 2.42–2.45), where *L*_A_(*m*) is an explicit function of *m* (see Online Resource 2, Eq. 2.46). The expression for *L*_A_(*m*) is however too complicated to be repeated here. The function *L*_A_(*m*) constitutes the boundary of the parameter domain with adult density overcompensation shown in the left panel of Fig. 3.

Furthermore, in Section II.4 of Online Resource 2, it is analysed whether an increase in mortality for juvenile consumers alone can give rise to an increase in juvenile or adult density at equilibrium. The analysis shows that an increase in mortality for juvenile consumers can lead to an increase in adult consumer density at equilibrium, depending on the value of the scaled quantities *q* := *β*/*γ*, *m*_J_ := *μ*_J_/*T* and *m*_A_ := *μ*_A_/*T*, where *μ*_J_ and *μ*_A_ indicate the stage-specific mortality rates for juvenile and adult consumers, respectively. The equilibrium juvenile consumer density will, however, always decrease with an increase in juvenile mortality *μ*_J_, independent of parameters.

Similarly, in Section II.5 of Online Resource 2, it is shown that an increase in mortality for adult consumers only can result in an increase in either juvenile or adult consumer density at equilibrium, depending on the parameters *q* := *β*/*γ*, *m*_J_ := *μ*_J_/*T* and *m*_A_ := *μ*_A_/*T*. An increase in juvenile consumer density with adult mortality is likely to occur when *q* := *β*/*γ* < 1, whereas an increase in adult consumer density at equilibrium with adult mortality can only occur for *q* := *β*/*γ* > 1.

## C.2 Stability of the equilibrium

The characteristic equation determining the stability of the non-trivial equilibrium is determined by the following characteristic polynomial in terms of the eigenvalue *λ*:

C.19 $$ \lambda^{3} + a_{1}\lambda^{2} + a_{2}\lambda + a_{3} = 0 $$

in which the coefficients *a*_1_, *a*_2_ and *a*_3_ are given by (see Online Resource 2, Eqs. 3.2–3.6):

C.20 $$\begin{array}{@{}rcl@{}} a_{1} &=& (\bar{C}_{\mathrm{J}}+\bar{C}_{\mathrm{A}}) f^{\prime}(\bar{R})+\gamma f(\bar{R})-T + 2\mu\\ a_{2} &=& ((\gamma f(\bar{R})-T + 2\mu) (\bar{C}_{\mathrm{J}}+\bar{C}_{\mathrm{A}})+\beta f(\bar{R}) \bar{C}_{\mathrm{A}})f^{\prime}(\bar{R})\\ \end{array} $$

$$\begin{array}{@{}rcl@{}} a_{3} &=& ((T(\beta f(\bar{R}) - T - \mu) + \mu (\gamma f(\bar{R}) + \mu)) \bar{C}_{\mathrm{J}}\\ && + (T(\gamma f(\bar{R})-T) + \mu (\gamma f(\bar{R})\\ && + \beta f(\bar{R}) - T + \mu))\bar{C}_{\mathrm{A}})f^{\prime}(\bar{R}) \end{array} $$

For systems of three ODEs, the Routh-Hurwitz conditions stipulate that the equilibrium is stable if *a*_1_ > 0, *a*_3_ > 0 and *a*_1_*a*_2_ − *a*_3_ > 0. Given that necessarily $\gamma f(\bar {R}) > T$ and $\beta f(\bar {R}) > T+\mu $ (see Eq. C.2), it is clear that the conditions *a*_1_ > 0 and *a*_3_ > 0 are satisfied.

The detailed calculations in Online Resource 2 (see Eqs. 3.8–3.19) show that *a*_1_*a*_2_ − *a*_3_ > 0 and hence the equilibrium is stable if:

C.21 $$ b_{1} P f^{\prime}(\bar{R}) + b_{2} > 0  $$

in which the coefficients *b*_1_ and *b*_2_ are given by:

C.22 $$\begin{array}{@{}rcl@{}} b_{1} \!&=&\! \frac{\gamma f(\bar{R})(\gamma f(\bar{R})\,-\,T+\mu) \,+\, 4(\beta f(\bar{R})\,-\,T) (\gamma f(\bar{R})\,-\,T)}{\gamma f(\bar{R})(\beta f(\bar{R})\,-\,T)}\\ && + \frac{3\mu(\beta f(\bar{R})-T)}{\gamma f(\bar{R})(\beta f(\bar{R})-T)} \end{array} $$

C.23 $$\begin{array}{@{}rcl@{}} b_{2} \!&=&\! -T (\beta f(\bar{R})\,-\,T)\! +\! (\gamma f(\bar{R})\,-\,T\,+\,\mu)^{2} \,+\,\mu (\gamma f(\bar{R})\,-\,T\,+\,2\mu)\\ && + \beta f(\bar{R}) (\gamma f(\bar{R})\,-\,T) \,+\, \frac{(\gamma f(\bar{R})\,-\,T\,+\,2\mu)^{2} (\gamma f(\bar{R})\,-\,T)}{\mu}\\ \end{array} $$

(Online Resource 2, Eqs. 3.18–3.19). Since in equilibrium $\gamma f(\bar {R}) > T$ and $\beta f(\bar {R}) > T+\mu $ (see Eq. C.2), the coefficient *b*_1_ is always positive. The coefficient *b*_2_, however, can be negative if the term $-T(\beta f(\bar {R})-T)$ is sufficiently negative, implying that adult fecundity $(\beta f(\bar {R})-T)$ has to be sufficiently large for population cycles to occur.

Condition (C.21) for stability of the non-trivial equilibrium can be expressed in terms of the three scaled quantities $h(\bar {R})=\gamma f(\bar {R})/T$, *q* = *β*/*γ* and *m* = *μ*/*T* as follows:

C.24 $$ \frac{\gamma P}{T^{2}} f^{\prime}(\bar{R}) > -\frac{c_{2}}{c_{1}}  $$

in which the coefficients *c*_1_ and *c*_2_ are given by the following:

C.25 $$\begin{array}{@{}rcl@{}} c_{1} &=& \frac{h(\bar{R})(h(\bar{R})-1+m)+ 4(q\,h(\bar{R})-1)(h(\bar{R})-1)+ 3m(q\,h(\bar{R})-1)}{h(\bar{R})(q\,h(\bar{R})-1)} \end{array} $$

C.26 $$\begin{array}{@{}rcl@{}} c_{2} &=&- (q\,h(\bar{R})-1)+(h(\bar{R})-1+m)^{2}+m(h(\bar{R})-1 + 2m)\\ && + q\,h(\bar{R})(h(\bar{R})-1)+\frac{(h(\bar{R})-1 + 2m)^{2}(h(\bar{R})-1)}{m} \end{array} $$

(Online Resource 2, Eqs. 3.21–3.26). The left-hand side of inequality (C.24) represents a scaled value of the resource productivity (scaled with $\gamma f^{\prime }(\bar {R})/T^{2}$) and can thus be set equal to any arbitrary value by an appropriate choice for *P*. On the other hand, the right-hand side of inequality (C.24) is only dependent on the dimensionless quantities *q* and *m*, given that $h(\bar {R})$ is also only dependent on *q* and *m* (see Eq. C.6). Therefore, if − *c*_2_/*c*_1_ is positive, values of the resource productivity *P* close to 0 would result in an unstable equilibrium and the occurrence of limit cycles in the consumer-resource system, whereas increasing the value of *P* will always lead to a stable equilibrium state. The right panel of Fig. 3 illustrates the results of this stability analysis, by showing for different values of $\gamma P f^{\prime }(\bar {R})/T^{2}$ the combinations of the parameters *q* and *m* for which the equilibrium is stable.
