# Supplementary material for: When individual life history matters: conditions for juvenile-adult stage structure effects on population dynamics
Source: Theor Ecol. 2018 May 4;11(4):397–416. doi: 10.1007/s12080-018-0374-3 (PMC6405019; doi:10.1007/s12080-018-0374-3)
Supplement: Supplementary file 3 — (PDF 645 KB) [file 12080_2018_374_MOESM3_ESM.pdf]

Online Resource 1: Different juvenile and adult foraging, maturation and reproduction rates

The following set of equations describes the general stage-structured model with separate functions $f_J(R)$ and $f_A(R)$ for juvenile and adult foraging, respectively, and separate functions $g_J(R)$ and $g_A(R)$ for juvenile maturation rate and adult reproduction rate, respectively. These functions are not further specified, it is only assumed that $\left(\frac{d}{dR} f_J(R)\right)$, $\left(\frac{d}{dR} f_A(R)\right)$, $\left(\frac{d}{dR} g_J(R)\right)$ and $\left(\frac{d}{dR} g_A(R)\right)$ are all non-negative, indicating that foraging, maturation and reproduction never decrease with resource density.

restart :

$$dxdt := \left[p(R) - f_J(R)\cdot C_J - f_A(R)\cdot C_A,\ g_A(R)\cdot C_A - g_J(R)\cdot C_J - \mu_J\cdot C_J,\ g_J(R)\cdot C_J - \mu_A\cdot C_A\right] : \left\langle\left\langle \frac{dR}{dt}, \frac{dC_J}{dt}, \frac{dC_A}{dt}\right\rangle\right\rangle = \langle\langle dxdt\rangle\rangle;$$

$$\begin{bmatrix} \frac{dR}{dt} \\ \frac{dC_J}{dt} \\ \frac{dC_A}{dt} \end{bmatrix} = \begin{bmatrix} p(R) - f_J(R)\, C_J - f_A(R)\, C_A \\ g_A(R)\, C_A - g_J(R)\, C_J - \mu_J\, C_J \\ g_J(R)\, C_J - \mu_A\, C_A \end{bmatrix} \quad (1)$$

For the function $p(R)$ it will be assumed that its derivative is 0 or negative, $\left(\frac{d}{dR} p(R)\right) \leq 0$, to ensure that classical predator-prey cycles such as occurring in the Rosenzweig-MacArthur model and the Hydra effect, which are both resulting from an increase in resource productivity with resource density, do not occur.

I exclude, however, the trivial case, in which the derivatives $\left(\frac{d}{dR} f_J(R)\right)$, $\left(\frac{d}{dR} f_A(R)\right)$ and $\left(\frac{d}{dR} p(R)\right)$ are all equal to 0 as this would make the dynamics of the resource completely independent of resource density itself.

I furthermore exclude the case, in which both functions $g_J(R)$ and $g_A(R)$ are independent of resource density R as this would lead to exponential growth of the consumer population.

I.1: Equilibrium

The equilibrium condition for the consumer, corresponding to the condition R0 = 1 looks like:

$$\frac{g_A(R)\cdot g_J(R)}{\mu_A} - \left(g_J(R) + \mu_J\right) = 0$$

$$\frac{g_A(R)\, g_J(R)}{\mu_A} - g_J(R) - \mu_J = 0 \quad (1.1)$$

$$EquiCond := \left(g_A(R) - \mu_A\right)\cdot g_J(R) - \mu_J\cdot\mu_A$$

$$\left(g_A(R) - \mu_A\right) g_J(R) - \mu_J\, \mu_A \quad (1.2)$$

Notice that the equilibrium condition above implies that $\left(g_A(R) - \mu_A\right)$ is positive as the product $\left(g_A(R) - \mu_A\right)\cdot g_J(R)$ should equal the positive quantity $\mu_J\cdot\mu_A$.

The equilibrium condition can also be expressed as:

$$EquiCond2 := g_J(R)\cdot g_A(R) = \left(g_J(R) + \mu_J\right)\cdot\mu_A$$

$$g_A(R)\, g_J(R) = \left(g_J(R) + \mu_J\right) \mu_A \tag{1.3}$$

Solve for the juvenile and adult density in equilibrium:

$Jeq := solve\left(subs\left(C_A = solve\left(dxdt[3], C_A\right), dxdt[1]\right), C_J\right);$

$$\frac{p(R)\, \mu_A}{f_J(R)\, \mu_A + f_A(R)\, g_J(R)} \tag{1.4}$$

$Aeq := subs\left(C_J = Jeq, solve\left(dxdt[3], C_A\right)\right)$

$$\frac{g_J(R)\, p(R)}{f_J(R)\, \mu_A + f_A(R)\, g_J(R)} \tag{1.5}$$

I.2: Overcompensation

To compute overcompensation the implicit function theorem is applied to the equilibrium conditions, as specified by the right-hand side of the 3 ODEs. Considering R, C_J, and C_A as a function of juvenile mortality μ_J, the change in equilibrium values of R, C_J, and C_A with increasing μ_J can be determined from the equation:

$$J \cdot \left[\frac{dR}{d\mu_J}, \frac{dC_J}{d\mu_J}, \frac{dC_A}{d\mu_J}\right]^T - \left[0, C_J, 0\right]^T = 0,$$

in which J is the Jacobian matrix of the system of ODEs.

Similarly, the change in equilibrium values of R, C_J, and C_A with increasing μ_A can be determined from the equation:

$$J \cdot \left[\frac{dR}{d\mu_A}, \frac{dC_J}{d\mu_A}, \frac{dC_A}{d\mu_A}\right]^T - \left[0, 0, C_A\right]^T = 0$$

The following vectors are hence needed to use the implicit function theorem to determine an increase in mortality of either juvenile or adult density

$vecJ := \langle 0, C_J, 0\rangle;\ vecA := \langle 0, 0, C_A\rangle;$

$$\begin{bmatrix} 0 \\ C_J \\ 0 \end{bmatrix}$$

$$\begin{bmatrix} 0 \\ 0 \\ C_A \end{bmatrix} \tag{2.1}$$

Compute the Jacobian of the system.

$J := VectorCalculus[Jacobian]\left(dxdt, \left[R, C_J, C_A\right]\right);$

$$\begin{bmatrix} \frac{d}{dR} p(R) - \left(\frac{d}{dR} f_J(R)\right) C_J - \left(\frac{d}{dR} f_A(R)\right) C_A & -f_J(R) & -f_A(R) \\ \left(\frac{d}{dR} g_A(R)\right) C_A - \left(\frac{d}{dR} g_J(R)\right) C_J & -g_J(R) - \mu_J & g_A(R) \\ \left(\frac{d}{dR} g_J(R)\right) C_J & g_J(R) & -\mu_A \end{bmatrix} \tag{2.2}$$

Compute its determinant

DetJ0 := *LinearAlgebra*[*Determinant*](*J*);

$$-\mu_A \mu_J C_A \left(\frac{d}{dR} f_A(R)\right) - \mu_A \mu_J C_J \left(\frac{d}{dR} f_J(R)\right) - \mu_A C_A \left(\frac{d}{dR} f_A(R)\right) g_J(R) - \mu_A C_A \left(\frac{d}{dR} g_A(R)\right) f_J(R) - \mu_A C_J \left(\frac{d}{dR} f_J(R)\right) g_J(R) + \mu_A C_J \left(\frac{d}{dR} g_J(R)\right) f_J(R) - \mu_J C_J \left(\frac{d}{dR} g_J(R)\right) f_A(R) + C_A \left(\frac{d}{dR} f_A(R)\right) g_A(R) g_J(R) - C_A \left(\frac{d}{dR} g_A(R)\right) f_A(R) g_J(R) + C_J \left(\frac{d}{dR} f_J(R)\right) g_A(R) g_J(R) - C_J g_A(R) \left(\frac{d}{dR} g_J(R)\right) f_J(R) + \mu_A \mu_J \left(\frac{d}{dR} p(R)\right) + \mu_A \left(\frac{d}{dR} p(R)\right) g_J(R) - \left(\frac{d}{dR} p(R)\right) g_A(R) g_J(R) \quad (2.3)$$

simplify(*DetJ0*, {*EquiCond*});

$$\left(-C_A \left(\frac{d}{dR} g_A(R)\right) f_J(R) + C_J f_J(R) \left(\frac{d}{dR} g_J(R)\right)\right) \mu_A - \mu_J C_J \left(\frac{d}{dR} g_J(R)\right) f_A(R) - C_J g_A(R) \left(\frac{d}{dR} g_J(R)\right) f_J(R) - C_A \left(\frac{d}{dR} g_A(R)\right) f_A(R) g_J(R) \quad (2.4)$$

DetJ1 := *collect*$\left(\%, \left[\left(\frac{d}{dR} f_J(R)\right), \left(\frac{d}{dR} f_A(R)\right), \left(\frac{d}{dR} g_J(R)\right), \left(\frac{d}{dR} g_A(R)\right), C_J, C_A\right]\right)$;

$$\left(f_J(R)\, \mu_A - f_A(R)\, \mu_J - f_J(R)\, g_A(R)\right) C_J \left(\frac{d}{dR} g_J(R)\right) + \left(-f_J(R)\, \mu_A - f_A(R)\, g_J(R)\right) C_A \left(\frac{d}{dR} g_A(R)\right) \quad (2.5)$$

DetJ2 := $-\left(f_J(R) \cdot \left(g_A(R) - \mu_A\right) + f_A(R) \cdot \mu_J\right) \left(\frac{d}{dR} g_J(R)\right) \cdot C_J - \left(f_A(R) \cdot g_J(R) + \mu_A \cdot f_J(R)\right) \cdot \left(\frac{d}{dR} g_A(R)\right) \cdot C_A$

$$-\left(f_J(R) \left(g_A(R) - \mu_A\right) + f_A(R)\, \mu_J\right) \left(\frac{d}{dR} g_J(R)\right) C_J - \left(f_J(R)\, \mu_A + f_A(R)\, g_J(R)\right) \left(\frac{d}{dR} g_A(R)\right) C_A \quad (2.6)$$

Check the correspondence of the last expression with the originally computed expression

simplify(*DetJ0* − *DetJ2*, {*EquiCond*});

$$0 \quad (2.7)$$

From expression (2.6) it is obvious that the determinant is strictly ***non-positive***, since in equilibrium the quantity $g_A(R) - \mu_A$ is necessarily positive, which implies that the coefficients in front of the derivatives $\left(\frac{d}{dR} g_J(R)\right)$ and $\left(\frac{d}{dR} g_A(R)\right)$ are both non-negative. Expression (2.6) also makes clear that the two derivatives $\left(\frac{d}{dR} g_J(R)\right)$ and $\left(\frac{d}{dR} g_A(R)\right)$ can not be simultaneously equal to 0. Otherwise, the maturation and reproduction rate would not change with resource density and the determinant of the Jacobian would equal 0, which would imply that the consumer population is unregulated. In this case a consumer-resource equilibrium either does not exists or the equilibrium corresponds to a bifurcation point. It will therefore be assumed that at least of of the derivatives $\left(\frac{d}{dR} g_J(R)\right)$ and $\left(\frac{d}{dR} g_A(R)\right)$ is strictly positive, which also implies that the determinant is strictly ***negative***.

Now determine the derivatives of the equilibrium densities with respect to the juvenile mortality μ_J by solving the equation that results from applying the implicit function theorem to the equilibirum condition:

$$J \cdot \left[\frac{dR}{d\mu_J}, \frac{dC_J}{d\mu_J}, \frac{dC_A}{d\mu_J}\right]^T - \left[0, C_J, 0\right]^T = 0$$

Since the solution of the linear system is derived by Maple using Cramer's rule, the determinant of the Jacobian matrix will occur in the denominator of each of the components of the solution. To simplify the analysis the solution is therefore multiplied with D, the determinant of the Jacobian. Remember that this determinant is ***negative*** (see equation (2.6)).

dxdmuJ := *simplify*(*DetJ0* · *LinearAlgebra*[*LinearSolve*](*J*, *vecJ*));

$$\left[\begin{array}{c} -\left(f_J(R)\,\mu_A + f_A(R)\,g_J(R)\right) C_J \\ C_J\left(\mu_A C_A\left(\frac{d}{dR} f_A(R)\right) + \mu_A C_J\left(\frac{d}{dR} f_J(R)\right) + C_J\left(\frac{d}{dR} g_J(R)\right) f_A(R) - \mu_A\left(\frac{d}{dR} p(R)\right)\right) \\ \left(\left(\frac{d}{dR} f_A(R)\right) C_A\, g_J(R) + \left(\frac{d}{dR} f_J(R)\right) C_J\, g_J(R) - C_J f_J(R)\left(\frac{d}{dR} g_J(R)\right) - \left(\frac{d}{dR} p(R)\right) g_J(R)\right) C_J \end{array}\right] \quad (2.8)$$

Consider the derivative of juvenile consumer density in equilibrium with respect to juvenile mortality rate, $\frac{dC_J}{d\mu_J}$:

$dCJdmuJ := collect\left(dxdmuJ[2], \left[\left(\frac{d}{dR} p(R)\right), \left(\frac{d}{dR} f_J(R)\right), \left(\frac{d}{dR} f_A(R)\right), \left(\frac{d}{dR} g_J(R)\right), \left(\frac{d}{dR} g_A(R)\right), C_J\right]\right);$

$$-C_J \mu_A\left(\frac{d}{dR} p(R)\right) + C_J^2 \mu_A\left(\frac{d}{dR} f_J(R)\right) + C_J \mu_A C_A\left(\frac{d}{dR} f_A(R)\right) + C_J^2\left(\frac{d}{dR} g_J(R)\right) f_A(R) \quad (2.9)$$

$dCJdmuJ := \left(\mu_A\cdot\left(-\left(\frac{d}{dR} p(R)\right) + \left(\frac{d}{dR} f_J(R)\right)\cdot C_J + \left(\frac{d}{dR} f_A(R)\right) C_A\right) + \left(\frac{d}{dR} g_J(R)\right)\cdot f_A(R)\cdot C_J\right)\cdot C_J$

$$\left(\mu_A\left(-\left(\frac{d}{dR} p(R)\right) + \left(\frac{d}{dR} f_J(R)\right) C_J + \left(\frac{d}{dR} f_A(R)\right) C_A\right) + C_J\left(\frac{d}{dR} g_J(R)\right) f_A(R)\right) C_J \quad (2.10)$$

$simplify(dCJdmuJ - \%\%);$

$$0 \quad (2.11)$$

Given that it is assumed that $\left(\frac{d}{dR} f_J(R)\right) \geq 0$, $\left(\frac{d}{dR} f_A(R)\right) \geq 0$, $\left(\frac{d}{dR} g_J(R)\right) \geq 0$ and $\left(\frac{d}{dR} p(R)\right) \leq 0$ and $\left(\frac{d}{dR} f_J(R)\right)$, $\left(\frac{d}{dR} f_A(R)\right)$ and $\left(\frac{d}{dR} p(R)\right)$ are not all equal to 0, it can be inferred from equation (2.10) that the derivative $\frac{dC_J}{d\mu_J}$ is strictly ***negative***, since the expression (2.10) for $D^{-1}\cdot\frac{dC_J}{d\mu_J}$ is positive and the determinant D of the Jacobian is negative.

Consider the derivative of adult consumer density in equilibrium with respect to juvenile mortality rate, $\frac{dC_A}{d\mu_J}$:

$dCAdmuJ := collect\left(dxdmuJ[3], \left[\left(\frac{d}{dR} p(R)\right), \left(\frac{d}{dR} f_J(R)\right), \left(\frac{d}{dR} f_A(R)\right), \left(\frac{d}{dR} g_J(R)\right), \left(\frac{d}{dR} g_A(R)\right), C_J\right]\right);$

$$-g_J(R)\, C_J\left(\frac{d}{dR} p(R)\right) + g_J(R)\, C_J^2\left(\frac{d}{dR} f_J(R)\right) + C_A\, g_J(R)\, C_J\left(\frac{d}{dR} f_A(R)\right) - C_J^2 f_J(R)\left(\frac{d}{dR} g_J(R)\right) \quad (2.12)$$

$dCAdmuJ := \left(g_J(R)\cdot\left(-\left(\frac{d}{dR} p(R)\right) + \left(\frac{d}{dR} f_J(R)\right) C_J + \left(\frac{d}{dR} f_A(R)\right) C_A\right) - f_J(R)\cdot\left(\frac{d}{dR} g_J(R)\right) C_J\right)\cdot C_J$

$$\left(g_J(R)\left(-\left(\frac{d}{dR} p(R)\right) + \left(\frac{d}{dR} f_J(R)\right) C_J + \left(\frac{d}{dR} f_A(R)\right) C_A\right) - C_J f_J(R)\left(\frac{d}{dR} g_J(R)\right)\right) C_J \quad (2.13)$$

$simplify(dCAdmuJ - \%\%);$

$$0 \quad (2.14)$$

$dCAdmuJ := g_J(R)\cdot\left(\left(-\left(\frac{d}{dR} p(R)\right) + \left(\frac{d}{dR} f_A(R)\right) C_A\right) + g_J(R)\cdot\left(\frac{d}{dR}\frac{(f_J(R))}{(g_J(R))}\right) C_J\right)\cdot C_J$

$$g_J(R)\left(-\left(\frac{d}{dR} p(R)\right) + \left(\frac{d}{dR} f_A(R)\right) C_A + g_J(R)\left(\frac{\frac{d}{dR} f_J(R)}{g_J(R)} - \frac{f_J(R)\left(\frac{d}{dR} g_J(R)\right)}{g_J(R)^2}\right) C_J\right) C_J \quad (2.15)$$

$simplify(dCAdmuJ - \%\%\%);$

$$0 \quad (2.16)$$

Given that it is assumed that $\left(\frac{d}{dR} f_A(R)\right) \geq 0$ and $\left(\frac{d}{dR} p(R)\right) \leq 0$ and $\left(\frac{d}{dR} f_J(R)\right)$, $\left(\frac{d}{dR} f_A(R)\right)$ and $\left(\frac{d}{dR} p(R)\right)$ are not all equal to 0, it can be inferred from expression (2.15) that $\frac{dC_A}{d\mu_J}$ is strictly ***negative***, as long

as the derivative $\left(\frac{d}{dR}\frac{(f_J(R))}{(g_J(R))}\right)$ is ***non-negative:***

$$\left(\frac{d}{dR}\frac{(f_J(R))}{(g_J(R))}\right) \geq 0$$

since under this condition expression (2.15) for $D^{-1}\cdot\frac{dC_A}{d\mu_J}$ is positive and the determinant D of the Jacobian is negative.

Now determine the derivatives of the equilibrium densities with respect to the adult mortality μ_A by solving the equation that results from applying the implicit function theorem to the equilibirum condition:

$$J\cdot\left[\frac{dR}{d\mu_A}, \frac{dC_J}{d\mu_A}, \frac{dC_A}{d\mu_A}\right]^T - \left[0, 0, C_A\right]^T = 0$$

As before, because the solution of the linear system is derived by Maple using Cramer's rule, the determinant of the Jacobian matrix will occur in the denominator of each of the components of the solution. To simplify the resulting expressions for the derivatives the solution is hence multiplied with D, the determinant of the Jacobian. Remember that this determinant is ***negative*** (see equation (2.6)).

$dxdmuA := simplify(DetJ0\cdot LinearAlgebra[LinearSolve](J, vecA));$

$$\Big[\big[-C_A\,(f_A(R)\,\mu_J + f_J(R)\,g_A(R) + f_A(R)\,g_J(R))\big],$$
$$\Big[C_A\Big(C_A\left(\frac{d}{dR}f_A(R)\right)g_A(R) - C_A\left(\frac{d}{dR}g_A(R)\right)f_A(R) + C_J\left(\frac{d}{dR}f_J(R)\right)g_A(R)$$
$$+ C_J\left(\frac{d}{dR}g_J(R)\right)f_A(R) - \left(\frac{d}{dR}p(R)\right)g_A(R)\Big)\Big],$$
$$\Big[\Big(\mu_J\,C_A\left(\frac{d}{dR}f_A(R)\right) + \mu_J\,C_J\left(\frac{d}{dR}f_J(R)\right) + \left(\frac{d}{dR}f_A(R)\right)C_A\,g_J(R) + C_A\left(\frac{d}{dR}g_A(R)\right)f_J(R)$$
$$+ \left(\frac{d}{dR}f_J(R)\right)C_J\,g_J(R) - C_J\,f_J(R)\left(\frac{d}{dR}g_J(R)\right) - \mu_J\left(\frac{d}{dR}p(R)\right) - \left(\frac{d}{dR}p(R)\right)g_J(R)\Big)\,C_A\Big]\Big] \quad (2.17)$$

Consider the derivative of juvenile consumer density in equilibrium with respect to adult mortality rate, $\frac{dC_J}{d\mu_A}$:

$dCJdmuA := collect\left(dxdmuA[2], \left[\left(\frac{d}{dR}p(R)\right), \left(\frac{d}{dR}f_J(R)\right), \left(\frac{d}{dR}f_A(R)\right), \left(\frac{d}{dR}g_J(R)\right), \left(\frac{d}{dR}g_A(R)\right)\right]\right);$

$$-g_A(R)\,C_A\left(\frac{d}{dR}p(R)\right) + C_A\,C_J\,g_A(R)\left(\frac{d}{dR}f_J(R)\right) + C_A^2\,g_A(R)\left(\frac{d}{dR}f_A(R)\right)$$
$$+ C_A\,C_J\,f_A(R)\left(\frac{d}{dR}g_J(R)\right) - C_A^2\left(\frac{d}{dR}g_A(R)\right)f_A(R) \quad (2.18)$$

$$dCJdmuA := \left(g_A(R)\cdot\left(-\left(\frac{d}{dR}p(R)\right) + \left(\frac{d}{dR}f_J(R)\right)\cdot C_J\right) + f_A(R)\cdot\left(\frac{d}{dR}g_J(R)\right)\cdot C_J + (g_A(R))^2\cdot\left(\frac{d}{dR}\frac{(f_A(R))}{(g_A(R))}\right)C_A\right)\cdot C_A$$

$$\left(g_A(R)\left(-\left(\frac{d}{dR}p(R)\right) + \left(\frac{d}{dR}f_J(R)\right)C_J\right) + C_J\left(\frac{d}{dR}g_J(R)\right)f_A(R) + g_A(R)^2\left(\frac{\frac{d}{dR}f_A(R)}{g_A(R)} - \frac{f_A(R)\left(\frac{d}{dR}g_A(R)\right)}{g_A(R)^2}\right)C_A\right)C_A \quad (2.19)$$

$simplify(dCJdmuA - \%\%);$

$$0 \qquad (2.20)$$

As before, given that it is assumed that $\left(\frac{d}{dR} f_J(R)\right) \geq 0$, $\left(\frac{d}{dR} f_A(R)\right) \geq 0$, $\left(\frac{d}{dR} g_J(R)\right) \geq 0$ and $\left(\frac{d}{dR} p(R)\right) \leq 0$ and $\left(\frac{d}{dR} f_J(R)\right)$, $\left(\frac{d}{dR} f_A(R)\right)$ and $\left(\frac{d}{dR} p(R)\right)$ are not all equal to 0, it can be inferred from expression (2.19) that the derivative $\frac{dC_J}{d\mu_A}$ is strictly ***negative***, as long as the derivative $\left(\frac{d}{dR} \frac{(f_A(R))}{(g_A(R))}\right)$ is ***non-negative:***

$$\left(\frac{d}{dR} \frac{(f_A(R))}{(g_A(R))}\right) \geq 0$$

since under this condition expression (2.19) for $D^{-1} \cdot \frac{dC_J}{d\mu_A}$ is positive and the determinant D of the Jacobian is negative.

Consider the derivative of adult consumer density in equilibrium with respect to adult mortality rate, $\frac{dC_A}{d\mu_A}$:

$$dCAdmuA := collect\left(dxdmuA[3], \left[\left(\frac{d}{dR} p(R)\right), \left(\frac{d}{dR} f_J(R)\right), \left(\frac{d}{dR} f_A(R)\right), \left(\frac{d}{dR} g_J(R)\right), \left(\frac{d}{dR} g_A(R)\right)\right]\right);$$

$$\left(-g_J(R) - \mu_J\right) C_A \left(\frac{d}{dR} p(R)\right) + \left(\mu_J C_J + g_J(R) C_J\right) C_A \left(\frac{d}{dR} f_A(R)\right) + \left(\mu_J C_A + C_A g_J(R)\right) C_A \left(\frac{d}{dR} f_J(R)\right) - C_J f_J(R) C_A \left(\frac{d}{dR} g_J(R)\right) + C_A^2 \left(\frac{d}{dR} g_A(R)\right) f_J(R) \qquad (2.21)$$

$$dCAdmuA := \left(\left(g_J(R) + \mu_J\right) \cdot \left(-\left(\frac{d}{dR} p(R)\right) + \left(\frac{d}{dR} f_A(R)\right) \cdot C_A\right) + \mu_J \left(\frac{d}{dR} f_J(R)\right) \cdot C_J + f_J(R) \cdot \left(\frac{d}{dR} g_A(R)\right) \cdot C_A + \left(g_J(R)\right)^2 \cdot \left(\frac{d}{dR} \frac{(f_J(R))}{(g_J(R))}\right) \cdot C_J\right) \cdot C_A$$

$$\left(\left(g_J(R) + \mu_J\right) \left(-\left(\frac{d}{dR} p(R)\right) + \left(\frac{d}{dR} f_A(R)\right) C_A\right) + \mu_J C_J \left(\frac{d}{dR} f_J(R)\right) + C_A \left(\frac{d}{dR} g_A(R)\right) f_J(R) + g_J(R)^2 \left(\frac{\frac{d}{dR} f_J(R)}{g_J(R)} - \frac{f_J(R) \left(\frac{d}{dR} g_J(R)\right)}{g_J(R)^2}\right) C_J\right) C_A \qquad (2.22)$$

$$simplify(dCAdmuA - \%\%);$$

$$0 \qquad (2.23)$$

Given that it is assumed that $\left(\frac{d}{dR} f_J(R)\right) \geq 0$,$\left(\frac{d}{dR} f_A(R)\right) \geq 0$, $\left(\frac{d}{dR} g_A(R)\right) \geq 0$ and$\left(\frac{d}{dR} p(R)\right) \leq 0$ and $\left(\frac{d}{dR} f_J(R)\right)$, $\left(\frac{d}{dR} f_A(R)\right)$ and$\left(\frac{d}{dR} p(R)\right)$ are not all equal to 0, it can be inferred from expression (2.22) that the derivative $\frac{dC_A}{d\mu_A}$ is strictly ***negative***, as long as the derivative $\left(\frac{d}{dR} \frac{(f_J(R))}{(g_J(R))}\right)$ is ***non-negative***:

$$\left(\frac{d}{dR} \frac{(f_J(R))}{(g_J(R))}\right) \geq 0$$

since under this condition expressions (2.22) for $D^{-1} \cdot \frac{dC_A}{d\mu_A}$ is positive and the determinant D of the Jacobian is negative.

Altogether the evaluation of these derivatives implies that any increase in mortality, be it juvenile or adult mortality, will only lead to decreases in both juvenile and adult density in equilibrium as long as the derivtives:

and

$$\left(\frac{d}{dR}\frac{(f_J(R))}{(g_J(R))}\right)$$

$$\left(\frac{d}{dR}\frac{(f_A(R))}{(g_A(R))}\right)$$

are non-negative.

If the inequality $\left(\frac{d}{dR}\frac{(f_J(R))}{(g_J(R))}\right) < 0$ **would hold, the juvenile maturation rate** $g_J(R)$ **would increase faster with an increase in resource density than the juvenile foraging rate** $f_J(R)$**. In other words, the efficiency with which juveniles can use acquired resources for maturation increases with resource density. If this condition holds the analysis above shows that adult equilibrium density can potentially increase with either juvenile or adult mortality.**

If the inequality $\left(\frac{d}{dR}\frac{(f_A(R))}{(g_A(R))}\right) < 0$ **would hold, the adult reproduction rate** $g_A(R)$ **would increase faster with an increase in resource density than the adult foraging rate** $f_A(R)$**. In other words, the efficiency with which adults can use acquired resources for reproduction increases with resource density. If this condition holds the analysis above shows that juvenile equilibrium density can potentially increase with an increase in adult mortality.**

Irrespective of these 2 derivatives, an increase in juvenile mortality rate will always lead to a decrease in juvenile equilibrium density.

I.3: Stability

Now consider the stability of the internal equilibrium. Can the structure give rise to cycles?

Determine the determinant of the stability matrix.

$S := J - \langle\langle \lambda, 0, 0\rangle | \langle 0, \lambda, 0\rangle | \langle 0, 0, \lambda\rangle\rangle;$

$$\begin{bmatrix} \frac{d}{dR}p(R) - \left(\frac{d}{dR}f_J(R)\right)C_J - \left(\frac{d}{dR}f_A(R)\right)C_A - \lambda & -f_J(R) & -f_A(R) \\ \left(\frac{d}{dR}g_A(R)\right)C_A - \left(\frac{d}{dR}g_J(R)\right)C_J & -g_J(R) - \mu_J - \lambda & g_A(R) \\ \left(\frac{d}{dR}g_J(R)\right)C_J & g_J(R) & -\mu_A - \lambda \end{bmatrix} \quad (3.1)$$

$LinearAlgebra[Determinant](S);$

$$-\mu_A \mu_J C_A\left(\frac{d}{dR}f_A(R)\right) - \mu_A \mu_J C_J\left(\frac{d}{dR}f_J(R)\right) - \mu_A C_A\left(\frac{d}{dR}f_A(R)\right)g_J(R) - \mu_A C_A\left(\frac{d}{dR}f_A(R)\right)\lambda \quad (3.2)$$
$$- \mu_A C_A\left(\frac{d}{dR}g_A(R)\right)f_J(R) - \mu_A C_J\left(\frac{d}{dR}f_J(R)\right)g_J(R) - \mu_A C_J\left(\frac{d}{dR}f_J(R)\right)\lambda$$
$$+ \mu_A C_J\left(\frac{d}{dR}g_J(R)\right)f_J(R) - \mu_J C_A\left(\frac{d}{dR}f_A(R)\right)\lambda - \mu_J C_J\left(\frac{d}{dR}f_J(R)\right)\lambda$$
$$- \mu_J C_J\left(\frac{d}{dR}g_J(R)\right)f_A(R) + C_A\left(\frac{d}{dR}f_A(R)\right)g_A(R)\,g_J(R) - C_A\left(\frac{d}{dR}f_A(R)\right)g_J(R)\,\lambda$$
$$- C_A\left(\frac{d}{dR}g_A(R)\right)f_J(R)\,\lambda - C_A\left(\frac{d}{dR}g_A(R)\right)f_A(R)\,g_J(R) + C_J\left(\frac{d}{dR}f_J(R)\right)g_A(R)\,g_J(R)$$
$$- C_J\left(\frac{d}{dR}f_J(R)\right)g_J(R)\,\lambda - C_J\,g_A(R)\left(\frac{d}{dR}g_J(R)\right)f_J(R) + C_J\left(\frac{d}{dR}g_J(R)\right)f_J(R)\,\lambda$$
$$- C_J\left(\frac{d}{dR}g_J(R)\right)f_A(R)\,\lambda - \mu_A \mu_J \lambda - C_A\left(\frac{d}{dR}f_A(R)\right)\lambda^2 - C_J\left(\frac{d}{dR}f_J(R)\right)\lambda^2$$
$$+ \mu_A \mu_J\left(\frac{d}{dR}p(R)\right) + \mu_A\left(\frac{d}{dR}p(R)\right)g_J(R) + \mu_A\left(\frac{d}{dR}p(R)\right)\lambda - \mu_A\,g_J(R)\,\lambda$$

$$+\mu_J\left(\frac{d}{dR}p(R)\right)\lambda-\left(\frac{d}{dR}p(R)\right)g_A(R)\,g_J(R)+\left(\frac{d}{dR}p(R)\right)g_J(R)\,\lambda+g_A(R)\,g_J(R)\,\lambda-\lambda^3$$
$$-\mu_A\lambda^2-\mu_J\lambda^2+\left(\frac{d}{dR}p(R)\right)\lambda^2-g_J(R)\,\lambda^2$$

Let Maple simplify this expression for the determinant using the equilibrium conditions for the resource density (equation (D.1.2)):

$simplify(LinearAlgebra[Determinant](S), \{EquiCond\})$

$$\left(-C_A f_J(R)\left(\frac{d}{dR}g_A(R)\right)-C_A\left(\frac{d}{dR}f_A(R)\right)\lambda-C_J\left(\frac{d}{dR}f_J(R)\right)\lambda+C_J\left(\frac{d}{dR}g_J(R)\right)f_J(R)\right.$$
$$+\left(\frac{d}{dR}p(R)\right)\lambda-\lambda^2\Big)\mu_A+\left(-C_A\left(\frac{d}{dR}f_A(R)\right)\lambda-C_J\left(\frac{d}{dR}f_J(R)\right)\lambda-C_J\left(\frac{d}{dR}g_J(R)\right)f_A(R)\right.$$
$$+\left(\frac{d}{dR}p(R)\right)\lambda-\lambda^2\Big)\mu_J-C_J\,g_A(R)\left(\frac{d}{dR}g_J(R)\right)f_J(R)+\left(-C_A f_A(R)\left(\frac{d}{dR}g_A(R)\right)\right.$$
$$-C_A\left(\frac{d}{dR}f_A(R)\right)\lambda-C_J\left(\frac{d}{dR}f_J(R)\right)\lambda+\left(\frac{d}{dR}p(R)\right)\lambda-\lambda^2\Big)g_J(R)$$
$$-C_A\left(\frac{d}{dR}g_A(R)\right)f_J(R)\,\lambda-C_A\left(\frac{d}{dR}f_A(R)\right)\lambda^2-C_J\left(\frac{d}{dR}f_J(R)\right)\lambda^2+C_J\left(\frac{d}{dR}g_J(R)\right)f_J(R)\,\lambda$$
$$-C_J\left(\frac{d}{dR}g_J(R)\right)f_A(R)\,\lambda+\left(\frac{d}{dR}p(R)\right)\lambda^2-\lambda^3 \qquad (3.3)$$

$CE := collect\left(\%, \left[\lambda, \frac{d}{dR}f_J(R), \frac{d}{dR}f_A(R), \frac{d}{dR}g_J(R), \frac{d}{dR}g_A(R), \frac{d}{dR}p(R)\right]\right);$

$$-\lambda^3+\left(-\left(\frac{d}{dR}f_A(R)\right)C_A-\left(\frac{d}{dR}f_J(R)\right)C_J-\mu_A-\mu_J+\frac{d}{dR}p(R)-g_J(R)\right)\lambda^2+\Big(\big(-C_J\,\mu_A$$
$$-\mu_J\,C_J-g_J(R)\,C_J\big)\left(\frac{d}{dR}f_J(R)\right)+\left(-\mu_A\,C_A-C_A\,\mu_J-C_A\,g_J(R)\right)\left(\frac{d}{dR}f_A(R)\right)+\big(f_J(R)\,C_J$$
$$-C_J f_A(R)\big)\left(\frac{d}{dR}g_J(R)\right)-C_A f_J(R)\left(\frac{d}{dR}g_A(R)\right)+\left(\mu_A+\mu_J+g_J(R)\right)\left(\frac{d}{dR}p(R)\right)\Big)\lambda$$
$$+\left(f_J(R)\,C_J\,\mu_A-\mu_J\,C_J f_A(R)-C_J\,g_A(R)\,f_J(R)\right)\left(\frac{d}{dR}g_J(R)\right)+\big(-\mu_A\,C_A f_J(R)$$
$$-C_A f_A(R)\,g_J(R)\big)\left(\frac{d}{dR}g_A(R)\right) \qquad (3.4)$$

Write this characteristic equation in a form $\lambda^3+a_1\cdot\lambda^2+a_2\cdot\lambda+a_3$ to be able to apply the Routh-Hurwitz criteria.

Define the coefficients a_1, a_2 and a_3:

$a_1 := -\left(\frac{d}{dR}p(R)\right)+\left(\frac{d}{dR}f_J(R)\right)C_J+\left(\frac{d}{dR}f_A(R)\right)C_A+g_J(R)+\mu_J+\mu_A;$

$$-\left(\frac{d}{dR}p(R)\right)+\left(\frac{d}{dR}f_J(R)\right)C_J+\left(\frac{d}{dR}f_A(R)\right)C_A+g_J(R)+\mu_J+\mu_A \qquad (3.5)$$

$a_2 := \left(g_J(R)+\mu_J+\mu_A\right)\cdot\left(-\left(\frac{d}{dR}p(R)\right)+\left(\frac{d}{dR}f_A(R)\right)\cdot C_A\right)+\left(\mu_J+\mu_A\right)\cdot\left(\frac{d}{dR}f_J(R)\right)\cdot C_J+f_A(R)\cdot\left(\frac{d}{dR}g_J(R)\right)\cdot C_J+g_J(R)^2\cdot\left(\frac{d}{dR}\frac{f_J(R)}{g_J(R)}\right)\cdot C_J+f_J(R)\cdot\left(\frac{d}{dR}g_A(R)\right)\cdot C_A;$

$$\left(\mu_A+\mu_J+g_J(R)\right)\left(-\left(\frac{d}{dR}p(R)\right)+\left(\frac{d}{dR}f_A(R)\right)C_A\right)+\left(\mu_J+\mu_A\right)\left(\frac{d}{dR}f_J(R)\right)C_J$$
$$+C_J\left(\frac{d}{dR}g_J(R)\right)f_A(R)+g_J(R)^2\left(\frac{\frac{d}{dR}f_J(R)}{g_J(R)}-\frac{f_J(R)\left(\frac{d}{dR}g_J(R)\right)}{g_J(R)^2}\right)C_J$$
$$+C_A f_J(R)\left(\frac{d}{dR}g_A(R)\right) \qquad (3.6)$$

$a_3 := \left(\left(g_A(R)-\mu_A\right)\cdot f_J(R)+\mu_J\cdot f_A(R)\right)\left(\frac{d}{dR}g_J(R)\right)\cdot C_J+\left(f_A(R)\cdot g_J(R)+\mu_A\cdot f_J(R)\right)\left(\frac{d}{dR}g_A(R)\right)\cdot C_A;$

$$\left(\left(g_A(R)-\mu_A\right)f_J(R)+\mu_J f_A(R)\right)\left(\frac{d}{dR}g_J(R)\right)C_J+\left(f_J(R)\,\mu_A+f_A(R)\,g_J(R)\right)\left(\frac{d}{dR}g_A(R)\right)C_A \qquad (3.7)$$

$simplify\left(\lambda^3+a_1\cdot\lambda^2+a_2\cdot\lambda+a_3+CE\right);$

$$0 \qquad (3.8)$$

Given that it is assumed that $\left(\frac{d}{dR}f_J(R)\right)\geq 0$, $\left(\frac{d}{dR}f_A(R)\right)\geq 0$, $\left(\frac{d}{dR}g_J(R)\right)\geq 0$, $\left(\frac{d}{dR}g_A(R)\right)\geq 0$, $\left(\frac{d}{dR}p(R)\right)\leq 0$, the derivatives $\left(\frac{d}{dR}f_J(R)\right)$, $\left(\frac{d}{dR}f_A(R)\right)$ and $\left(\frac{d}{dR}p(R)\right)$ are not all equal to 0, and the equilibrium conditions requires that $g_A(R)>\mu_A$, the expressions for a_1, a_2 and a_3 only include strictly non-negative terms and are hence guaranteed to be positive, if in addition the derivative

$$\left(\frac{d}{dR}\frac{\left(f_J(R)\right)}{\left(g_J(R)\right)}\right)\geq 0$$

as this additional condition would guarantee the positivity of the coefficient a_2.

The Routh-Hurwitz criteria stipulate that the equilibrium of a 3-dimensional ODE system is stable if $a_1>0$, $a_3>0$ and $a_1\cdot a_2>a_3$. Clearly, the first two conditions are satisfied, irrespective of the value of $\left(\frac{d}{dR}\frac{\left(f_J(R)\right)}{\left(g_J(R)\right)}\right)$.

Check the last condition $a_1\cdot a_2>a_3$:

$RH3 := collect\left(simplify\left(a_1\cdot a_2-a_3,\{EquiCond\}\right),\left[\frac{d}{dR}p(R),\ \frac{d}{dR}f_J(R),\ \frac{d}{dR}g_J(R),\ \frac{d}{dR}f_A(R),\ \frac{d}{dR}g_A(R),\ C_J,\ C_A\right]\right)$

$$\begin{aligned}&\left(\mu_A+\mu_J+g_J(R)\right)\left(\frac{d}{dR}p(R)\right)^2+\Bigg(\left(\left(-2\mu_A-2\mu_J-2g_J(R)\right)C_J\left(\frac{d}{dR}f_J(R)\right)+\left(f_J(R)\right.\right.\\&\left.-f_A(R)\right)C_J\left(\frac{d}{dR}g_J(R)\right)+\left(-2\mu_A-2\mu_J-2g_J(R)\right)C_A\left(\frac{d}{dR}f_A(R)\right)-C_A f_J(R)\left(\frac{d}{dR}g_A(R)\right)\\&\left.-\mu_A^2-2\mu_J\mu_A-2g_J(R)\mu_A-\mu_J^2-2\mu_J g_J(R)-g_J(R)^2\right)\left(\frac{d}{dR}p(R)\right)+\left(\mu_A+\mu_J\right.\\&\left.+g_J(R)\right)C_J^2\left(\frac{d}{dR}f_J(R)\right)^2+\left(\left(-f_J(R)+f_A(R)\right)C_J^2\left(\frac{d}{dR}g_J(R)\right)+\left(2\mu_A+2\mu_J\right.\right.\\&\left.+2g_J(R)\right)C_A C_J\left(\frac{d}{dR}f_A(R)\right)+C_A C_J f_J(R)\left(\frac{d}{dR}g_A(R)\right)+\left(\mu_A^2+2\mu_J\mu_A+2g_J(R)\mu_A\right.\\&\left.\left.+\mu_J^2+2\mu_J g_J(R)+g_J(R)^2\right)C_J\right)\left(\frac{d}{dR}f_J(R)\right)+\left(\left(-f_J(R)+f_A(R)\right)C_A C_J\left(\frac{d}{dR}f_A(R)\right)\right.\\&\left.+\left(f_A(R)\mu_A-f_J(R)\mu_J-f_J(R)g_A(R)+\left(-f_J(R)+f_A(R)\right)g_J(R)\right)C_J\right)\left(\frac{d}{dR}g_J(R)\right)+\left(\mu_A+\mu_J\right.\\&\left.+g_J(R)\right)C_A^2\left(\frac{d}{dR}f_A(R)\right)^2+\left(C_A^2 f_J(R)\left(\frac{d}{dR}g_A(R)\right)+\left(\mu_A^2+2\mu_J\mu_A+2g_J(R)\mu_A+\mu_J^2\right.\right.\\&\left.\left.+2\mu_J g_J(R)+g_J(R)^2\right)C_A\right)\left(\frac{d}{dR}f_A(R)\right)+\left(f_J(R)\mu_J+\left(f_J(R)\right.\right.\\&\left.\left.-f_A(R)\right)g_J(R)\right)C_A\left(\frac{d}{dR}g_A(R)\right)\end{aligned} \qquad (3.9)$$

This complicated expression for $a_1\cdot a_2-a_3$ consists of 8 terms, but is rewritten below with the help of a lot of manual manipulations as a sum of 9 terms:

$$x_1+x_2+x_3+x_4+x_5+x_6+x_7+x_8+x_9$$

Notice that these manipulations that result in the terms x_1, x_2, x_3, x_4, x_5, x_6, x_7, x_8 and x_9 make use of the identity

(1.3):

$$g_J(R)\cdot g_A(R) = \left(g_J(R) + \mu_J\right)\cdot \mu_A$$

that can be derived from the equilibrium conditions of the model.

The additional term x_9 includes only terms that depend on the derivatives $\left(\frac{d}{dR}\frac{(f_J(R))}{(g_J(R))}\right)$ and $\left(\frac{d}{dR}\frac{(f_A(R))}{(g_A(R))}\right)$ and is composed from parts of the terms numbered 2, 3, 4, 5, 7 and 8 of the original expression for $a_1\cdot a_2 - a_3$.

$$x_1 := \left(g_J(R) + \mu_J + \mu_A\right)\left(\frac{d}{dR}p(R)\right)^2;$$

$$\left(\mu_A + \mu_J + g_J(R)\right)\left(\frac{d}{dR}p(R)\right)^2 \tag{3.10}$$

$$x_2 := \left(\left(g_J(R) + 2\cdot\mu_J + 2\cdot\mu_A\right)\left(\frac{d}{dR}f_J(R)\right)\cdot C_J + f_A(R)\cdot\left(\frac{d}{dR}g_J(R)\right)\cdot C_J + 2\cdot\left(g_J(R) + \mu_J + \mu_A\right)\cdot\left(\frac{d}{dR}f_A(R)\right)\cdot C_A + \left(\frac{d}{dR}g_A(R)\right)f_J(R)\cdot C_A + \left(g_J(R) + \mu_J + \mu_A\right)^2\right)\left(-\frac{d}{dR}p(R)\right);$$

$$-\left(\left(g_J(R) + 2\,\mu_J + 2\,\mu_A\right)\left(\frac{d}{dR}f_J(R)\right)C_J + C_J\left(\frac{d}{dR}g_J(R)\right)f_A(R) + 2\left(\mu_A + \mu_J + g_J(R)\right)\left(\frac{d}{dR}f_A(R)\right)C_A + C_A f_J(R)\left(\frac{d}{dR}g_A(R)\right) + \left(\mu_A + \mu_J + g_J(R)\right)^2\right)\left(\frac{d}{dR}p(R)\right) \tag{3.11}$$

$$x_3 := \left(\mu_J + \mu_A\right)\cdot\left(\frac{d}{dR}f_J(R)\right)^2\cdot C_J^2;$$

$$\left(\mu_J + \mu_A\right)\left(\frac{d}{dR}f_J(R)\right)^2 C_J^2 \tag{3.12}$$

$$x_4 := \left(f_A(R)\cdot\left(\frac{d}{dR}g_J(R)\right)\cdot C_J + \left(g_J(R) + 2\cdot\mu_J + 2\cdot\mu_A\right)\cdot\left(\frac{d}{dR}f_A(R)\right)\cdot C_A + f_J(R)\cdot\left(\frac{d}{dR}g_A(R)\right)\cdot C_A + \left(\mu_A\,g_J(R) + \mu_J\,g_J(R) + \mu_A^2 + \mu_J\,\mu_A + \mu_J^2\right)\right)\left(\frac{d}{dR}f_J(R)\right)\cdot C_J;$$

$$\left(C_J\left(\frac{d}{dR}g_J(R)\right)f_A(R) + \left(g_J(R) + 2\,\mu_J + 2\,\mu_A\right)\left(\frac{d}{dR}f_A(R)\right)C_A + C_A f_J(R)\left(\frac{d}{dR}g_A(R)\right) + g_J(R)\,\mu_A + \mu_J\,g_J(R) + \mu_A^2 + \mu_J\,\mu_A + \mu_J^2\right)\left(\frac{d}{dR}f_J(R)\right)C_J \tag{3.13}$$

$$x_5 := \left(f_A(R)\cdot C_A\cdot\left(\frac{d}{dR}f_A(R)\right) + f_A(R)\cdot\left(g_J(R) + \mu_A\right)\right)\left(\frac{d}{dR}g_J(R)\right)\cdot C_J;$$

$$\left(f_A(R)\,C_A\left(\frac{d}{dR}f_A(R)\right) + f_A(R)\left(g_J(R) + \mu_A\right)\right)\left(\frac{d}{dR}g_J(R)\right)C_J \tag{3.14}$$

$$x_6 := \left(g_J(R) + \mu_A + \mu_J\right)C_A^2\left(\frac{d}{dR}f_A(R)\right)^2;$$

$$\left(\mu_A + \mu_J + g_J(R)\right)C_A^2\left(\frac{d}{dR}f_A(R)\right)^2 \tag{3.15}$$

$$x_7 := \left(\left(\frac{d}{dR}g_A(R)\right)f_J(R)\cdot C_A + \left(g_J(R)^2 + \mu_A\,g_J(R) + 2\,\mu_J\,g_J(R) + \mu_A^2 + \mu_J\,\mu_A + \mu_J^2\right)\right)\left(\frac{d}{dR}f_A(R)\right)\cdot C_A$$

$$\left(C_A f_J(R)\left(\frac{d}{dR}g_A(R)\right) + g_J(R)^2 + g_J(R)\,\mu_A + 2\,\mu_J\,g_J(R) + \mu_A^2 + \mu_J\,\mu_A + \mu_J^2\right)\left(\frac{d}{dR}f_A(R)\right)C_A \tag{3.16}$$

$$x_8 := \left(\left(g_J(R) + \mu_J\right)\cdot f_J(R)\right)\cdot\left(\frac{d}{dR}g_A(R)\right)\cdot C_A;$$

$$\left(g_J(R) + \mu_J\right)f_J(R)\left(\frac{d}{dR}g_A(R)\right)C_A \tag{3.17}$$

$$x_9 := \left(-\left(\frac{d}{dR}p(R)\right) + \left(\frac{d}{dR}f_J(R)\right)\cdot C_J + \left(\frac{d}{dR}f_A(R)\right)\cdot C_A + \left(g_J(R) + g_A(R) + \mu_J\right)\right)\cdot g_J(R)^2\cdot\left(\frac{d}{dR}\frac{(f_J(R))}{(g_J(R))}\right)\cdot C_J + g_J(R)\cdot g_A(R)^2\cdot\left(\frac{d}{dR}\frac{(f_A(R))}{(g_A(R))}\right)\cdot C_A$$

$$\left(-\left(\frac{d}{dR}p(R)\right)+\left(\frac{d}{dR}f_J(R)\right)C_J+\left(\frac{d}{dR}f_A(R)\right)C_A+g_J(R)+g_A(R)+\mu_J\right)g_J(R)^2\left(\frac{\frac{d}{dR}f_J(R)}{g_J(R)}-\frac{f_J(R)\left(\frac{d}{dR}g_J(R)\right)}{g_J(R)^2}\right)C_J+g_J(R)\,g_A(R)^2\left(\frac{\frac{d}{dR}f_A(R)}{g_A(R)}-\frac{f_A(R)\left(\frac{d}{dR}g_A(R)\right)}{g_A(R)^2}\right)C_A \tag{3.18}$$

Finally check that the sum $x_1+x_2+x_3+x_4+x_5+x_6+x_7+x_8+x_9$ is indeed equivalent to the original expression for $a_1\cdot a_2-a_3$, while taking into account that in equilibrium $g_J(R)\cdot g_A(R)=\left(g_J(R)+\mu_J\right)\cdot\mu_A$:

$simplify\left(x_1+x_2+x_3+x_4+x_5+x_6+x_7+x_8+x_9-RH3,\ \{EquiCond2\}\right);$

$$0 \tag{3.19}$$

Given that it is assumed that $\left(\frac{d}{dR}f_J(R)\right)\geq 0$, $\left(\frac{d}{dR}f_A(R)\right)\geq 0$, $\left(\frac{d}{dR}g_J(R)\right)\geq 0$, $\left(\frac{d}{dR}g_A(R)\right)\geq 0$ and $\left(\frac{d}{dR}p(R)\right)\leq 0$, inspection of the terms x_1, x_2, x_3, x_4, x_5, x_6, x_7 and x_8 reveals that all these 8 terms are ***non-negative*** while the assumption that the derivatives $\left(\frac{d}{dR}f_J(R)\right)$, $\left(\frac{d}{dR}f_A(R)\right)$ and $\left(\frac{d}{dR}p(R)\right)$ are not all equal to 0 guarantees that the sum

$$x_1+x_2+x_3+x_4+x_5+x_6+x_7+x_8$$

is strictly positive.

In addition, with $\left(\frac{d}{dR}f_J(R)\right)\geq 0$, $\left(\frac{d}{dR}f_A(R)\right)\geq 0$, $\left(\frac{d}{dR}g_J(R)\right)\geq 0$, $\left(\frac{d}{dR}g_A(R)\right)\geq 0$ and $\left(\frac{d}{dR}p(R)\right)\leq 0$ and $\left(\frac{d}{dR}f_J(R)\right)$, $\left(\frac{d}{dR}f_A(R)\right)$ and $\left(\frac{d}{dR}p(R)\right)$ not all equal to 0, the last term x_9 is non-negative as long as the derivatives

$$\left(\frac{d}{dR}\frac{(f_J(R))}{(g_J(R))}\right)$$

and

$$\left(\frac{d}{dR}\frac{(f_A(R))}{(g_A(R))}\right)$$

are non-negative, which in turn would imply that the sum

$$x_1+x_2+x_3+x_4+x_5+x_6+x_7+x_8+x_9$$

is strictly positive and hence that the third of the Routh-Hurwitz conditions is satisfied.

In summary, assuming that $\left(\frac{d}{dR}f_J(R)\right)\geq 0$, $\left(\frac{d}{dR}f_A(R)\right)\geq 0$, $\left(\frac{d}{dR}g_J(R)\right)\geq 0$, $\left(\frac{d}{dR}g_A(R)\right)\geq 0$ and $\left(\frac{d}{dR}p(R)\right)\leq 0$ with the derivatives $\left(\frac{d}{dR}f_J(R)\right)$, $\left(\frac{d}{dR}f_A(R)\right)$ and $\left(\frac{d}{dR}p(R)\right)$ not simultaneously equal to 0, the non-trivial equilibrium of the stage-structured model is always stable as long as the derivatives:

$$\left(\frac{d}{dR}\frac{(f_J(R))}{(g_J(R))}\right)$$

and

$$\left(\frac{d}{dR}\frac{(f_A(R))}{(g_A(R))}\right)$$

are non-negative.

Therefore, population cycles can only occur if the efficiency with which juveniles can use acquired resources for maturation increases with resource density (i.e. the juvenile maturation rate $g_J(R)$ **increases faster with an increase in resource density than the juvenile foraging rate** $f_J(R)$ **implying that** $\left(\frac{\mathrm{d}}{\mathrm{d}R} \frac{\left(f_J(R)\right)}{\left(g_J(R)\right)} \right) < 0$ **) or the efficiency with which adults can use acquired resources for reproduction increases with resource density (equivalent to the adult reproduction rate** $g_A(R)$ **would increase faster with an increase in resource density than the adult foraging rate** $f_A(R)$ **and** $\left(\frac{\mathrm{d}}{\mathrm{d}R} \frac{\left(f_A(R)\right)}{\left(g_A(R)\right)} \right) < 0$).
