# Supplementary material for: When individual life history matters: conditions for juvenile-adult stage structure effects on population dynamics
Source: Theor Ecol. 2018 May 4;11(4):397–416. doi: 10.1007/s12080-018-0374-3 (PMC6405019; doi:10.1007/s12080-018-0374-3)
Supplement: Supplementary file 4 — (PDF 1.82 MB) [file 12080_2018_374_MOESM4_ESM.pdf]

Online Resource 2: Consumer maturation and reproduction reduced by somatic maintenance costs

The following set of equations represents the model formulation, in which juveniles and adults are assumed to have equal foraging and mortality rates and equal maintenance costs, while the resource productivity is assumed to be constant, independent of resource density itself:

$restart:$

$dxdt := \left[P - f(R)\cdot C_J - f(R)\cdot C_A, (\beta\cdot f(R) - T)\cdot C_A - (\gamma\cdot f(R) - T)\cdot C_J - \mu\cdot C_J, (\gamma\cdot f(R) - T)\cdot C_J - \mu\cdot C_A\right] : \left\langle\left\langle \frac{dR}{dt}, \frac{dC_J}{dt}, \frac{dC_A}{dt}\right\rangle\right\rangle = \langle\langle dxdt\rangle\rangle;$

$$\begin{bmatrix}\frac{dR}{dt}\\ \frac{dC_J}{dt}\\ \frac{dC_A}{dt}\end{bmatrix} = \begin{bmatrix} P - f(R)\, C_J - f(R)\, C_A \\ (\beta f(R) - T)\, C_A - (\gamma f(R) - T)\, C_J - \mu\, C_J \\ (\gamma f(R) - T)\, C_J - \mu\, C_A\end{bmatrix} \tag{1}$$

For the function $f(R)$ it is again assumed that it is increasing with R: $\left(\frac{d}{dR} f(R)\right) > 0$.

II.1: Equilibrium

The equilibrium condition for the consumer, signifying R0 = 1 looks like:

$\frac{(\beta f(R) - T)\cdot(\gamma f(R) - T)}{\mu} - ((\gamma f(R) - T) + \mu)$

$$\frac{(\beta f(R) - T)\,(\gamma f(R) - T)}{\mu} - \gamma f(R) + T - \mu \tag{1.1}$$

$EquiCond := (\beta f(R) - T - \mu)\cdot(\gamma f(R) - T) - \mu^2$

$$(\beta f(R) - T - \mu)\,(\gamma f(R) - T) - \mu^2 \tag{1.2}$$

$simplify(EquiCond - \mu\cdot\%\%);$

$$0 \tag{1.3}$$

Notice that the equilibrium condition above implies that both $(\beta f(R) - T - \mu)$ as well as $(\gamma f(R) - T)$ are positive as their product should equal the positive quantity μ^2. Because maturation should at least be positive, $\gamma f(R) > T$, $(\beta f(R) - T - \mu)$ and $(\gamma f(R) - T)$ can not both be negative. In fact, the first term of the equilibrium condition starts for $f(R) = 0$ at the positive value equal to $T\cdot(T + \mu)$. For increasing values of $f(R)$ this first term will become negative for $\min\left(\frac{(T+\mu)}{\beta}, \frac{T}{\gamma}\right) < f(R) < \max\left(\frac{(T+\mu)}{\beta}, \frac{T}{\gamma}\right)$ and will become once again positive for $f(R) > \max\left(\frac{(T+\mu)}{\beta}, \frac{T}{\gamma}\right)$. The value of $f(R)$ at equilibrium is hence given by

the root of the quadratic equation $(\beta \cdot x - T - \mu) \cdot (\gamma \cdot x - T) = \mu^2$ for which $x > \max\left(\frac{(T+\mu)}{\beta}, \frac{T}{\gamma}\right)$.

Solve for the value of the function response $f(R)$ at equilibrium:

$solve(subs(f(R) = x, EquiCond), x);$

$$\frac{1}{2} \frac{1}{\gamma \beta}\left(T \beta + T \gamma + \gamma \mu + \sqrt{T^2 \beta^2 - 2 T^2 \beta \gamma + T^2 \gamma^2 - 2 T \beta \gamma \mu + 2 T \gamma^2 \mu + 4 \beta \gamma \mu^2 + \gamma^2 \mu^2}\right), -\frac{1}{2} \frac{1}{\gamma \beta}\left(-T \gamma - \gamma \mu - T \beta + \sqrt{T^2 \beta^2 - 2 T^2 \beta \gamma + T^2 \gamma^2 - 2 T \beta \gamma \mu + 2 T \gamma^2 \mu + 4 \beta \gamma \mu^2 + \gamma^2 \mu^2}\right) \quad \textbf{(1.4)}$$

The equilibrium value is given by the larger of these two solutions

$$Fequi := \frac{\left((\beta+\gamma) T + \gamma \cdot \mu + \mathrm{sqrt}\left(((\beta+\gamma) T + \gamma \cdot \mu)^2 - 4 \cdot \beta \cdot \gamma \cdot (T^2 + T \cdot \mu - \mu^2)\right)\right)}{2 \cdot \beta \cdot \gamma}$$

$$\frac{1}{2} \frac{(\beta+\gamma) T + \gamma \mu + \sqrt{((\beta+\gamma) T + \gamma \mu)^2 - 4 \gamma \beta (T^2 + T \mu - \mu^2)}}{\gamma \beta} \quad \textbf{(1.5)}$$

$simplify(Fequi - \%\%[1]);$

$$0 \quad \textbf{(1.6)}$$

Solve for the juvenile and adult density in equilibrium.

$Jeq := solve\left(subs\left(C_A = solve\left(dxdt[3], C_A\right), dxdt[1]\right), C_J\right);$

$$\frac{P \mu}{f(R) (\gamma f(R) - T + \mu)} \quad \textbf{(1.7)}$$

$subs\left(C_J = Jeq, solve\left(dxdt[3], C_A\right)\right)$

$$\frac{(\gamma f(R) - T) P}{f(R) (\gamma f(R) - T + \mu)} \quad \textbf{(1.8)}$$

$$Aeq := \frac{P \cdot (\gamma f(R) - T)}{f(R) (\gamma f(R) - T + \mu)}$$

$$\frac{(\gamma f(R) - T) P}{f(R) (\gamma f(R) - T + \mu)} \quad \textbf{(1.9)}$$

Now define $m = \frac{\mu}{T}$, $q = \frac{\beta}{\gamma}$, and $h(R) = \frac{\gamma \cdot f(R)}{T}$. Then, the equilibrium condition can be written as:

$$EquiCond1 := \frac{(q \cdot h(R) - 1)(h(R) - 1)}{m} = (h(R) - 1 + m)$$

$$\frac{(q\, h(R) - 1)(h(R) - 1)}{m} = h(R) - 1 + m \quad \textbf{(1.10)}$$

This shows that the dependence is completely determined by the 2 dimensionless parameters q and

m.

Solve the equilibrium condition in terms of $h(R) = \frac{\gamma \cdot f(R)}{T}$ for $h(R)$:

solve(EquiCond1, h(R));

$$\frac{1}{2}\,\frac{m+q+1+\sqrt{4\,m^2\,q+m^2-2\,m\,q+q^2+2\,m-2\,q+1}}{q},\;-\frac{1}{2}\,\frac{-m-q-1+\sqrt{4\,m^2\,q+m^2-2\,m\,q+q^2+2\,m-2\,q+1}}{q} \quad \textbf{(1.11)}$$

$hequi := \frac{\left(m+q+1+\sqrt{(m+q+1)^2-4\cdot q\cdot(1+m-m^2)}\right)}{2\cdot q}$; $simplify(hequi - \%\%[1])$

$$\frac{1}{2}\,\frac{m+q+1+\sqrt{(m+q+1)^2-4\,q\,(-m^2+m+1)}}{q}$$

$$0 \quad \textbf{(1.12)}$$

Check the obtained root for equality to the original expression in terms of β, γ and μ.

$simplify\left(subs\left(m = \frac{\mu}{T}, q = \frac{\beta}{\gamma}, hequi\right) - \frac{\gamma}{T}\cdot Fequi\right)$ assuming *positive*;

$$0 \quad \textbf{(1.13)}$$

II.2: Overcompensation

To compute overcompensation the implicit function theorem is applied to the equilibrium conditions, as specified by the right-hand side of the 3 ODEs. Considering R, C_J, and C_A a function of stage-independent mortality μ, the change in equilibrium values of R, C_J, and C_A with an increasing in μ can be solved determined with the equation:

$$J\cdot\left[\frac{dR}{d\mu}, \frac{dC_J}{d\mu}, \frac{dC_A}{d\mu}\right]^T - \left[0, C_J, C_A\right]^T = 0$$

in which J is the Jacobian matrix of the system of ODEs.

$vecJA := \langle 0, C_J, C_A\rangle$;

$$\begin{bmatrix} 0 \\ C_J \\ C_A \end{bmatrix} \quad \textbf{(2.1)}$$

The vector above is needed to use the implicit function theorem to determine an increase in mortality of either juvenile or adult density

Compute the Jacobian

$J := VectorCalculus[Jacobian](dxdt, [R, C_J, C_A]);$

$$\begin{bmatrix} -\left(\frac{d}{dR} f(R)\right) C_J - \left(\frac{d}{dR} f(R)\right) C_A & -f(R) & -f(R) \\ \beta \left(\frac{d}{dR} f(R)\right) C_A - \gamma \left(\frac{d}{dR} f(R)\right) C_J & -\gamma f(R) + T - \mu & \beta f(R) - T \\ \gamma \left(\frac{d}{dR} f(R)\right) C_J & \gamma f(R) - T & -\mu \end{bmatrix} \quad (2.2)$$

Compute its determinant

$DetJ0 := collect\left(LinearAlgebra[Determinant](J), \left[\frac{d}{dR} f(R), C_J, C_A\right]\right);$

$$\left(\left(-f(R)\,\gamma\,\mu - f(R)\,T\,\beta + T^2 + T\,\mu - \mu^2\right) C_J + \left(-f(R)\,\gamma\,T - f(R)\,\gamma\,\mu - f(R)\,\beta\,\mu + T^2 + T\,\mu - \mu^2\right) C_A\right) \left(\frac{d}{dR} f(R)\right) \quad (2.3)$$

$DetJ1 := -\left(\left(\mu\cdot(\gamma\cdot f(R) - T + \mu) + T\cdot(\beta\cdot f(R) - T)\right)\cdot C_J + \left(T\cdot(\gamma\cdot f(R) - T) + \mu\cdot(\gamma\cdot f(R) + \beta\cdot f(R) - T + \mu)\right)\cdot C_A\right)\cdot\left(\frac{d}{dR} f(R)\right)$

$$-\left(\left(\mu\,(\gamma f(R) - T + \mu) + T\,(\beta f(R) - T)\right) C_J + \left(T\,(\gamma f(R) - T) + \mu\,(\gamma f(R) + \beta f(R) - T + \mu)\right) C_A\right) \left(\frac{d}{dR} f(R)\right) \quad (2.4)$$

$simplify(DetJ0 - DetJ1);$

$$0 \quad (2.5)$$

Clearly, the determinant is always ***negative*** irrespective of positive or zero maintenance costs T, as the equilibrium condition ensures that both $(\beta f(R) - T)$ as well as $(\gamma f(R) - T)$ are positive, while $\left(\frac{d}{dR} f(R)\right) > 0$ by assumption.

Now determine the derivatives of the equilibrium densities with respect to the stage-independent mortality rate μ by solving the equation that results from applying the implicit function theorem to the equilibirum condition:

$$J\cdot\left[\frac{dR}{d\mu}, \frac{dC_J}{d\mu}, \frac{dC_A}{d\mu}\right]^T - [0, C_J, C_A]^T = 0$$

Since the solution of the linear system is derived by Maple using Cramer's rule, the determinant of the Jacobian matrix will occur in the denominator of each of the components of the solution. To simplify the expression the solution is multiplied with -D (minus the determinant). Because the determinant of the Jacobian matrix is negative (see equation (2.4)) this preserves the sign of the derivatives.

$dxdmu := simplify(-DetJ0\cdot LinearAlgebra[LinearSolve](J, vecJA));$

$$\left[\left[f(R)\,\left(C_A f(R)\,\gamma + C_A f(R)\,\beta + C_J f(R)\,\gamma - 2\,C_A\,T + \mu\,C_A - C_J\,T + \mu\,C_J\right)\right],\right. \quad (2.6)$$

$$\left[\left(\frac{d}{dR}f(R)\right)\left(-C_A C_J f(R)\gamma - C_A C_J f(R)\beta - C_J^2 f(R)\gamma + C_A^2 T + C_A C_J T - C_A C_J \mu - C_J^2 \mu\right)\right],$$

$$\left[-\left(\frac{d}{dR}f(R)\right)\left(C_A^2 f(R)\gamma + C_A^2 f(R)\beta + C_A C_J f(R)\gamma - C_A^2 T + C_A^2 \mu - 2 C_A C_J T + C_A C_J \mu - C_J^2 T\right)\right]\Bigg]$$

$$dCJdmu := collect\left(dxdmu[2], \left[\frac{d}{dR}\, f(R), C_J, C_A\right]\right);$$

$$\left(\left(-\gamma f(R) - \mu\right) C_J^2 + \left(-\gamma f(R) - \beta f(R) + T - \mu\right) C_A C_J + C_A^2 T\right)\left(\frac{d}{dR}f(R)\right) \quad \textbf{(2.7)}$$

$$dCJdmu := -\left(\left(\gamma \cdot f(R) + \mu\right)\cdot C_J^2 + \left(\left(\gamma \cdot f(R) + \mu\right) + \left(\beta \cdot f(R) - T\right)\right)\cdot C_J \cdot C_A - T \cdot C_A^2\right) \cdot \left(\frac{d}{dR}f(R)\right)$$

$$-\left(\left(\gamma f(R) + \mu\right) C_J^2 + \left(\gamma f(R) + \beta f(R) - T + \mu\right) C_J C_A - C_A^2 T\right)\left(\frac{d}{dR}f(R)\right) \quad \textbf{(2.8)}$$

$$simplify(dCJdmu - \%\%);$$

$$0 \quad \textbf{(2.9)}$$

The derivative of C_J in equilibrium with respect to the mortality rate μ hence equals:

$$-\left(D^{-1}\right)\cdot dCJdmu$$

$$\frac{\left(\left(\gamma f(R) + \mu\right) C_J^2 + \left(\gamma f(R) + \beta f(R) - T + \mu\right) C_J C_A - C_A^2 T\right)\left(\frac{d}{dR}f(R)\right)}{D} \quad \textbf{(2.10)}$$

This derivative is positive as long as the first term in parenthesis is ***negative*** (remember that $D < 0$) :

$$\left(\gamma f(R) + \mu\right) C_J^2 + \left(\gamma f(R) + \beta f(R) - T + \mu\right) C_J C_A - C_A^2 T < 0$$

$$\left(\gamma f(R) + \mu\right) C_J^2 + \left(\gamma f(R) + \beta f(R) - T + \mu\right) C_J C_A - C_A^2 T < 0 \quad \textbf{(2.11)}$$

Use the relation between the equilibrium values of C_J and C_A, $C_A = \frac{\left(\gamma \cdot f(R) - T\right)}{\mu} \cdot C_J$, to eliminate C_A from the left-0hand side of this inequality. This leads to:

$$dCJdmu1 := \left(\left(\gamma \cdot f(R) + \mu\right) + \left(\left(\gamma \cdot f(R) + \mu\right) + \left(\beta \cdot f(R) - T\right)\right) \cdot \frac{\left(\gamma \cdot f(R) - T\right)}{\mu} - T \cdot \frac{\left(\gamma \cdot f(R) - T\right)^2}{\mu^2}\right)$$

$$\gamma f(R) + \mu + \frac{\left(\gamma f(R) + \beta f(R) - T + \mu\right)\left(\gamma f(R) - T\right)}{\mu} - \frac{T\left(\gamma f(R) - T\right)^2}{\mu^2} \quad \textbf{(2.12)}$$

$$simplify\left(dCJdmu + dCJdmu1 \cdot C_J^2 \cdot \left(\frac{d}{dR} f(R)\right), \left\{C_A = \frac{(\gamma \cdot f(R) - T)}{\mu} \cdot C_J\right\}\right);$$

$$0 \tag{2.13}$$

Therefore, juvenile density is increasing with mortality as long as the following inequality holds:

$$dCJdmu1 < 0;$$

$$\gamma f(R) + \mu + \frac{(\gamma f(R) + \beta f(R) - T + \mu)\,(\gamma f(R) - T)}{\mu} - \frac{T\,(\gamma f(R) - T)^2}{\mu^2} < 0 \tag{2.14}$$

From the equilibrium condition it follows that $\frac{(\beta f(R) - T)\,(\gamma f(R) - T)}{\mu} = \gamma f(R) - T + \mu$, which allows simplification of the expression above to:

$$(\gamma f(R) + 2 \cdot \mu) - \frac{T}{\mu} \cdot \left(\frac{(\gamma f(R) - T)}{(\gamma f(R) - T + \mu)}\right) \cdot (\gamma f(R) - T) < 0$$

$$\gamma f(R) + 2\,\mu - \frac{T\,(\gamma f(R) - T)^2}{\mu\,(\gamma f(R) - T + \mu)} < 0 \tag{2.15}$$

$$simplify\left(lhs(\%) - \frac{\mu}{(\gamma f(R) - T + \mu)} \cdot dCJdmu1, \left\{\frac{(\beta f(R) - T)\,(\gamma f(R) - T)}{\mu} = \gamma f(R) - T + \mu\right\}\right)$$

$$0 \tag{2.16}$$

Because the equilibrium condition can also be expressed as $\frac{(\gamma f(R) - T)}{(\gamma f(R) - T + \mu)} \cdot \frac{(\beta \cdot f(R) - T)}{\mu} = 1$, the term $\frac{(\gamma f(R) - T)}{(\gamma f(R) - T + \mu)}$ can be replaced by $\frac{\mu}{(\beta \cdot f(R) - T)}$.

$$(\gamma f(R) + 2 \cdot \mu) - T \cdot \left(\frac{1}{(\beta \cdot f(R) - T)}\right) \cdot (\gamma f(R) - T) < 0$$

$$\gamma f(R) + 2\,\mu - \frac{T\,(\gamma f(R) - T)}{\beta f(R) - T} < 0 \tag{2.17}$$

This shows that the juvenile consumer density in equilibrium will increase with an increase in mortality as long as:

$$\frac{(\beta \cdot f(R) - T)}{(\gamma f(R) - T)} < \frac{T}{\gamma f(R) + 2 \cdot \mu}$$

$$\frac{\beta f(R) - T}{\gamma f(R) - T} < \frac{T}{\gamma f(R) + 2\,\mu} \tag{2.18}$$

Because in equilibrium necessarily $\gamma \cdot f(R) > T$, it can be inferred that $\frac{T}{\gamma \cdot f(R) + 2 \cdot \mu} < \frac{T}{T + 2 \cdot \mu}$. Therefore, for overcompensation in juvenile density to occur the ratio

$\dfrac{(\beta \cdot f(R) - T)}{(\gamma f(R) - T)}$ should necessarily be smaller than $\dfrac{T}{T + 2 \cdot \mu} = \dfrac{1}{1 + \dfrac{2 \cdot \mu}{T}}$. Since the latter quantity is smaller than 1, it follows that for overcompensation in juvenile density to occur also necessarily $\beta < \gamma$.

Hence, overcompensation in juvenile density in response to an increase in mortality occurs as long as:

$$\frac{(\beta \cdot f(R) - T)}{(\gamma f(R) - T)} < \frac{T}{\gamma f(R) + 2\,\mu} < \frac{T}{T + 2 \cdot \mu} < 1$$

Using the scaled quantities $m = \dfrac{\mu}{T}$, $q = \dfrac{\beta}{\gamma}$, and $h(R) = \dfrac{\gamma \cdot f(R)}{T}$, the condition for the occurrence of overcompensation in juvenile density can be written as:

$$\frac{q \cdot h(R) - 1}{h(R) - 1} < \frac{1}{h(R) + 2 \cdot m}$$

$$\frac{q\,h(R) - 1}{h(R) - 1} < \frac{1}{h(R) + 2\,m} \qquad \textbf{(2.19)}$$

As for the equilibrium condition, the condition is completely determined by the 2 dimensionless parameters q and m.

Consider the limit of the condition for the occurrence of juvenile overcompensation:

$$\frac{q \cdot h(R) - 1}{h(R) - 1} = \frac{1}{h(R) + 2 \cdot m}$$

$$\frac{q\,h(R) - 1}{h(R) - 1} = \frac{1}{h(R) + 2\,m} \qquad \textbf{(2.20)}$$

Which can be rewritten as:

$$(q \cdot h(R) - 1) \cdot (h(R) + 2 \cdot m) - (h(R) - 1) = 0$$

$$(q\,h(R) - 1)\,(h(R) + 2\,m) - h(R) + 1 = 0 \qquad \textbf{(2.21)}$$

$simplify(\text{expand}(\text{lhs}(\%)), \{EquiCond1\});$

$$2\,q\,h(R)\,m + m^2 + h(R)\,m + q\,h(R) - 3\,m - h(R) \qquad \textbf{(2.22)}$$

$Jincrease := m^2 - (3 - (1 + 2 \cdot q) \cdot h(R)) \cdot m - (1 - q) \cdot h(R);\ simplify(\%\% - Jincrease);$

$$m^2 - (3 - (1 + 2\,q)\,h(R))\,m - (1 - q)\,h(R)$$

$$0 \qquad \textbf{(2.23)}$$

Solve the condition for juvenile overcompensaiton to occur for q.

$sols := solve(subs(h(R) = hequi, Jincrease), q)$ assuming $m :: positive,\ q :: positive;$

$$\frac{1}{2}\,\frac{-m^4 - 2\,m^3 + m^2 - 4\,m - 2 + \sqrt{m^8 + 4\,m^7 - 46\,m^6 + 60\,m^5 + 45\,m^4}}{4\,m^4 - 2\,m^3 - 2\,m^2 - 2\,m - 1}, \qquad \textbf{(2.24)}$$

$$-\frac{1}{2}\,\frac{m^4 + 2\,m^3 - m^2 + \sqrt{m^8 + 4\,m^7 - 46\,m^6 + 60\,m^5 + 45\,m^4} + 4\,m + 2}{4\,m^4 - 2\,m^3 - 2\,m^2 - 2\,m - 1}$$

Two roots are obtained that are both functions in terms of m. Plot the two functions:

$plot([sols[1], sols[2]], m = 0..1, view = [0..1, 0..2], color = ["Navy", "Burgundy"], labels = [m, q])$

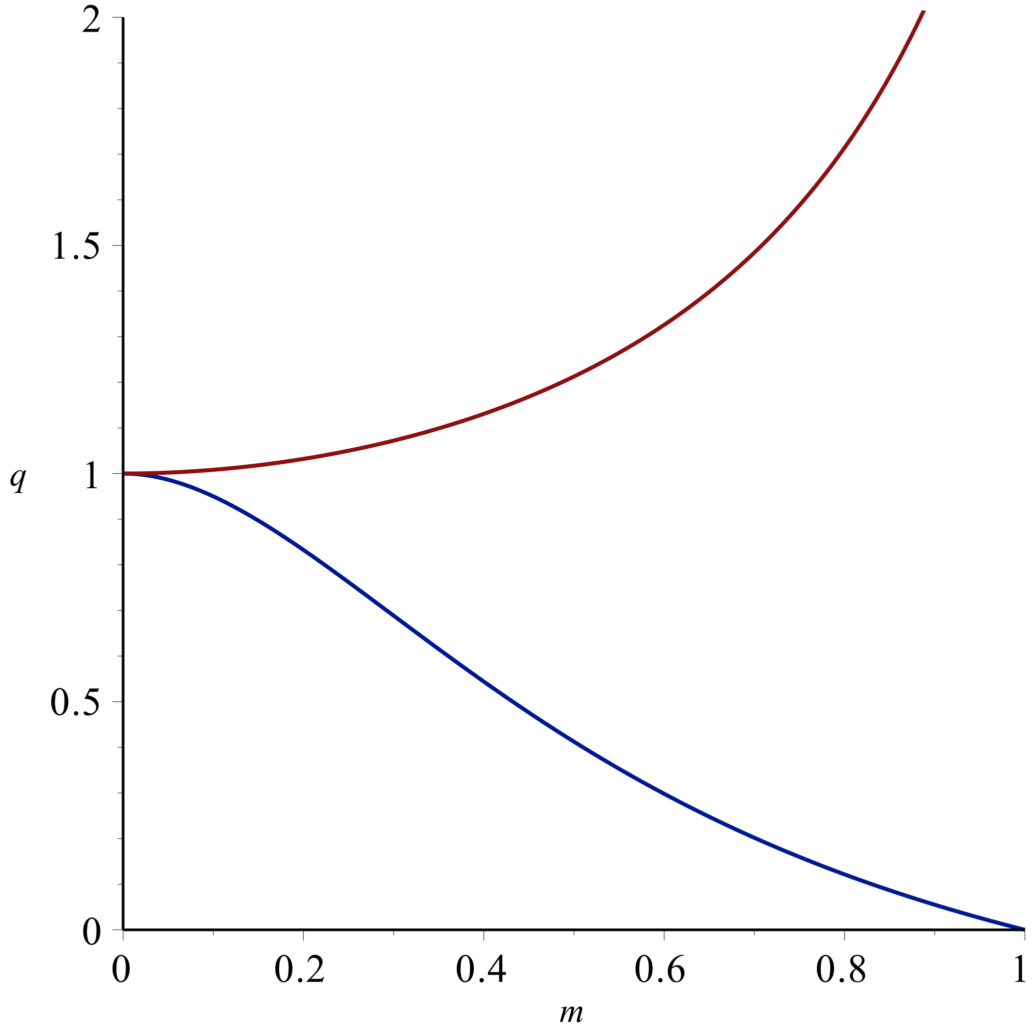

From these graphs it can be inferred that the second solution for q is a spurious one, as it provides values that are larger than 1, whereas it was derived above that for juvenile overcompensation to occur necessarily $\beta < \gamma$, which implies $0 < q < 1$.

This means that the limits to juvenile overcompensation are given by:

$Jbnd := sols[1] : q < Jbnd;$

$$q < \frac{1}{2} \frac{-m^4 - 2m^3 + m^2 - 4m - 2 + \sqrt{m^8 + 4m^7 - 46m^6 + 60m^5 + 45m^4}}{4m^4 - 2m^3 - 2m^2 - 2m - 1} \quad \textbf{(2.25)}$$

$plot(Jbnd, m = 0..5, view = [0..1, 0..1], labels = [m, q])$

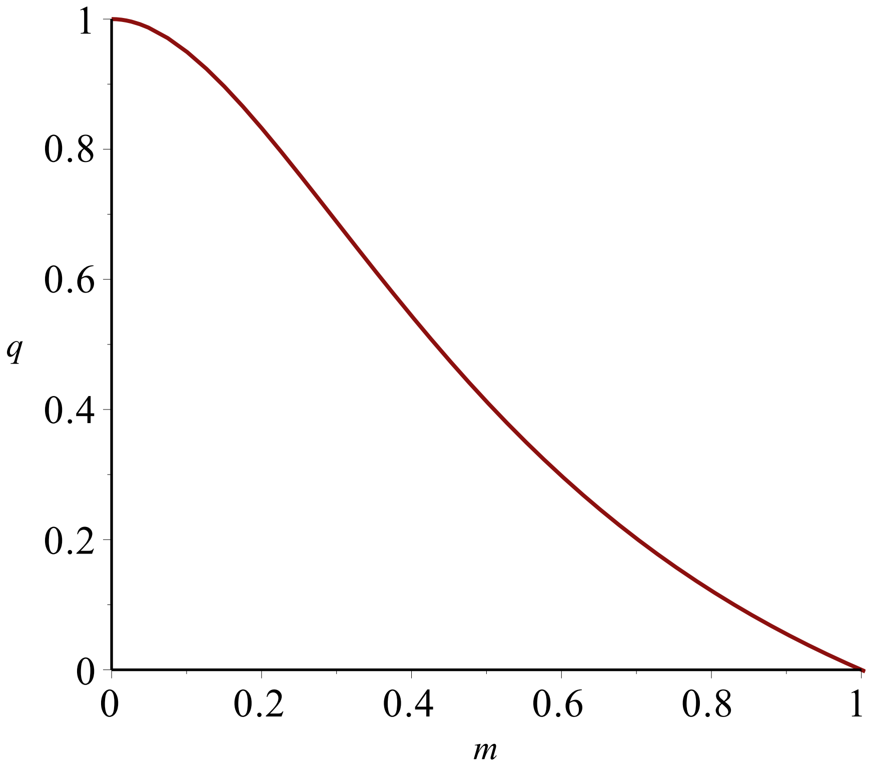

Now turn to a potential increase in adult density with increasing mortality

$$dCAdmu := collect\left(dxdmu[3], \left[\frac{d}{dR} f(R), C_J, C_A\right]\right);$$

$$\left(C_J^2\, T + (-\gamma f(R) + 2\,T - \mu)\, C_A\, C_J + (-\gamma f(R) - \beta f(R) + T - \mu)\, C_A^2\right) \left(\frac{d}{dR} f(R)\right) \quad \textbf{(2.26)}$$

The derivative of C_A in equilibrium with respect to the mortality rate μ hence equals:

$$-(\mathrm{D}^{-1}) \cdot dCAdmu$$

$$-\frac{1}{\mathrm{D}}\left(\left(C_J^2\, T + (-\gamma f(R) + 2\,T - \mu)\, C_A\, C_J + (-\gamma f(R) - \beta f(R) + T - \mu)\, C_A^2\right) \left(\frac{d}{dR} f(R)\right)\right) \quad \textbf{(2.27)}$$

which is positive as long as the following condition holds (notice that $\mathrm{D} < 0$):

$$(\gamma f(R) + \beta f(R) - T + \mu)\, C_A^2 + (\gamma f(R) - 2\,T + \mu)\, C_A\, C_J - T \cdot C_J^2 < 0$$

$$(\gamma f(R) + \beta f(R) - T + \mu)\, C_A^2 + (\gamma f(R) - 2\,T + \mu)\, C_A\, C_J - C_J^2\, T < 0 \quad \textbf{(2.28)}$$

Use the relation between the equilibrium values of C_J and C_A, $C_A = \frac{(\gamma \cdot f(R) - T)}{\mu} \cdot C_J$, to eliminate C_J from the left-hand side this inequality. This leads to:

$$dCAdmu1 := \left((\gamma f(R) + \beta f(R) - T + \mu) + \frac{\mu \cdot (\gamma f(R) - 2\,T + \mu)}{(\gamma \cdot f(R) - T)} - \frac{T \cdot \mu^2}{(\gamma \cdot f(R) - T)^2} \right)$$

$$\gamma f(R) + \beta f(R) - T + \mu + \frac{\mu\,(\gamma f(R) - 2\,T + \mu)}{\gamma f(R) - T} - \frac{T\,\mu^2}{(\gamma f(R) - T)^2} \tag{2.29}$$

$$simplify\left(dCAdmu + dCAdmu1 \cdot \left(\frac{d}{dR} f(R) \right) \cdot C_A^{\,2}, \left\{ C_A = \frac{(\gamma \cdot f(R) - T)}{\mu} \cdot C_J \right\} \right)$$

$$0 \tag{2.30}$$

Therefore, adult density is increasing with mortality as long as the following inequality holds:

$$dCAdmu1 < 0$$

$$\gamma f(R) + \beta f(R) - T + \mu + \frac{\mu\,(\gamma f(R) - 2\,T + \mu)}{\gamma f(R) - T} - \frac{T\,\mu^2}{(\gamma f(R) - T)^2} < 0 \tag{2.31}$$

Rearranging the terms in the left-hand side lead to:

$$\gamma f(R) + 2 \cdot \mu + \beta \cdot f(R) - T + \frac{\mu^2 \cdot (\gamma f(R) - T) - \mu \cdot T \cdot (\gamma f(R) - T + \mu)}{(\gamma f(R) - T)^2} < 0$$

$$\gamma f(R) + 2\,\mu + \beta f(R) - T + \frac{\mu^2\,(\gamma f(R) - T) - \mu\,T\,(\gamma f(R) - T + \mu)}{(\gamma f(R) - T)^2} < 0 \tag{2.32}$$

$$simplify\left(lhs(\%) - dCAdmu1, \left\{ \frac{(\beta f(R) - T)\,(\gamma f(R) - T)}{\mu} = \gamma f(R) - T + \mu \right\} \right)$$

$$0 \tag{2.33}$$

The last term of the inequality can furthermore be rewritten using the equilibrium condition that

$$\frac{(\beta f(R) - T)\,(\gamma f(R) - T)}{\mu} = \gamma f(R) - T + \mu.$$

This then leads to the following form of the inequality:

$$\gamma f(R) + 2 \cdot \mu + \beta \cdot f(R) - T + \frac{\mu^2 - T \cdot (\beta f(R) - T)}{(\gamma f(R) - T)} < 0$$

$$\gamma f(R) + 2\,\mu + \beta f(R) - T + \frac{\mu^2 - T\,(\beta f(R) - T)}{\gamma f(R) - T} < 0 \tag{2.34}$$

$$\gamma f(R) + \mu + \frac{(2 \cdot \gamma f(R) - 3 \cdot T) \cdot (\beta f(R) - T)}{(\gamma f(R) - T)} < 0$$

$$\gamma f(R) + \mu + \frac{(2\,\gamma f(R) - 3\,T)\,(\beta f(R) - T)}{\gamma f(R) - T} < 0 \tag{2.35}$$

Verify the result

$$simplify\left(lhs(\%) - dCAdmu1, \left\{ \frac{(\beta f(R) - T)\,(\gamma f(R) - T)}{\mu} = \gamma f(R) - T + \mu \right\} \right)$$

$$0 \tag{2.36}$$

Given that in equilibrium $\beta f(R) - T > 0$ and $\gamma f(R) - T > 0$ the inequality

$$\gamma f(R) + \mu + \frac{(2\cdot\gamma f(R) - 3\cdot T)\cdot(\beta f(R) - T)}{(\gamma f(R) - T)} < 0$$

can only hold if $2\cdot\gamma f(R) - 3\cdot T < 0$. Therefore, necessarily adult density can only increase with increasing mortality if: $T < \gamma\cdot f(R) < \frac{3}{2}\cdot T$.

Furthermore, in addition to this condition, the following inequality should hold:

$$\frac{\beta f(R) - T}{\gamma f(R) - T} > \frac{\gamma f(R) + \mu}{3\cdot T - 2\cdot \gamma f(R)}$$

$$\frac{\gamma f(R) + \mu}{3\, T - 2\, \gamma f(R)} < \frac{\beta f(R) - T}{\gamma f(R) - T} \tag{2.37}$$

$$simplify\left(rhs(\%) - lhs(\%) - \frac{dCAdmu1}{2\cdot \gamma f(R) - 3\cdot T}, \left\{ \frac{(\beta f(R) - T)\,(\gamma f(R) - T)}{\mu} = \gamma f(R) - T + \mu \right\}\right)$$

$$0 \tag{2.38}$$

Hence, overcompensation in adult density in response to an increase in mortality occurs as long as:

$$\frac{(\beta\cdot f(R) - T)}{(\gamma f(R) - T)} > \frac{\gamma f(R) + \mu}{3\cdot T - 2\cdot \gamma f(R)} \qquad \text{and} \qquad T < \gamma\cdot f(R) < \frac{3}{2}\cdot T$$

Now substitute $m := \frac{\mu}{T}$, $q := \frac{\beta}{\gamma}$ and $h(R) = \frac{\gamma}{T}\cdot f(R)$. Then, the inequality above can be written as:

$$\frac{(q\cdot h(R) - 1)}{(h(R) - 1)} > \frac{(h(R) + m)}{3 - 2\cdot h(R)}$$

$$\frac{h(R) + m}{3 - 2\, h(R)} < \frac{q\, h(R) - 1}{h(R) - 1} \tag{2.39}$$

The boundary in parameter space delineating the parameter regions with and without overcompensation in adult density in response to increasing mortality is hence given by:

$$Aincrease := lhs(\%) - rhs(\%)$$

$$\frac{h(R) + m}{3 - 2\, h(R)} - \frac{q\, h(R) - 1}{h(R) - 1} \tag{2.40}$$

Solve the above equation together with the equilibrium condition:

EquiCond1

$$\frac{(q\, h(R) - 1)\,(h(R) - 1)}{m} = h(R) - 1 + m \tag{2.41}$$

for the values of $h(R)$ and q:

$sols := solve(\{Aincrease, EquiCond1\}, \{h(R), q\})$ assuming $m :: positive$;

$$\left\{q = \frac{1}{m\,(3\,m-4)}\Big(4\,m^4 + 6\,m^3\,RootOf\big(\_Z^3 + (3\,m-2)\,\_Z^2 + (2\,m^2 - 7\,m + 1)\,\_Z - 3\,m^2 + 4\,m\big) + 2\,m^2\,RootOf\big(\_Z^3 + (3\,m-2)\,\_Z^2 + (2\,m^2 - 7\,m + 1)\,\_Z - 3\,m^2 + 4\,m\big)^2 - 11\,m^3 - 10\,m^2\,RootOf\big(\_Z^3 + (3\,m-2)\,\_Z^2 + (2\,m^2 - 7\,m + 1)\,\_Z - 3\,m^2 + 4\,m\big) - 3\,RootOf\big(\_Z^3 + (3\,m-2)\,\_Z^2 + (2\,m^2 - 7\,m + 1)\,\_Z - 3\,m^2 + 4\,m\big)^2\,m + 10\,m^2 + 5\,m\,RootOf\big(\_Z^3 + (3\,m-2)\,\_Z^2 + (2\,m^2 - 7\,m + 1)\,\_Z - 3\,m^2 + 4\,m\big) + RootOf\big(\_Z^3 + (3\,m-2)\,\_Z^2 + (2\,m^2 - 7\,m + 1)\,\_Z - 3\,m^2 + 4\,m\big)^2 - 6\,m - 2\,RootOf\big(\_Z^3 + (3\,m-2)\,\_Z^2 + (2\,m^2 - 7\,m + 1)\,\_Z - 3\,m^2 + 4\,m\big) + 1\Big), h(R) = RootOf\big(\_Z^3 + (3\,m-2)\,\_Z^2 + (2\,m^2 - 7\,m + 1)\,\_Z - 3\,m^2 + 4\,m\big)\right\} \quad \textbf{(2.42)}$$

This solution shows that there are potentially 3 different solutions for $h(R)$ given by the roots of the polynomial

$$\_Z^3 + (3\,m-2)\,\_Z^2 + (2\,m^2 - 7\,m + 1)\,\_Z - 3\,m^2 + 4\,m = 0$$

This polynomial only depends on m and is not a function of q at all. Given a value of m and a corresponding solution of this polynomial for $h(R)$ that is appropriate, the corresponding value of q is uniquely determined by the equation:

$subs(rhs(op(2, sols)) = h(R), op(1, sols))$

$$q = \frac{1}{m\,(3\,m-4)}\Big(4\,m^4 + 6\,m^3\,h(R) + 2\,m^2\,h(R)^2 - 11\,m^3 - 10\,m^2\,h(R) - 3\,h(R)^2\,m + 10\,m^2 + 5\,m\,h(R) + h(R)^2 - 6\,m - 2\,h(R) + 1\Big) \quad \textbf{(2.43)}$$

Plot the 3 roots of the pylonomial $\_Z^3 + (3\,m-2)\,\_Z^2 + (2\,m^2 - 7\,m + 1)\,\_Z - 3\,m^2 + 4\,m = 0$ as a function of m:

$hvals := [allvalues(rhs(op(2, sols)))]:$
$plot(hvals, m = 0..2, color = [$"Navy", "Burgundy", "Olive"$], labels = [m, h(R)], view = [0..2, -1..2]);$

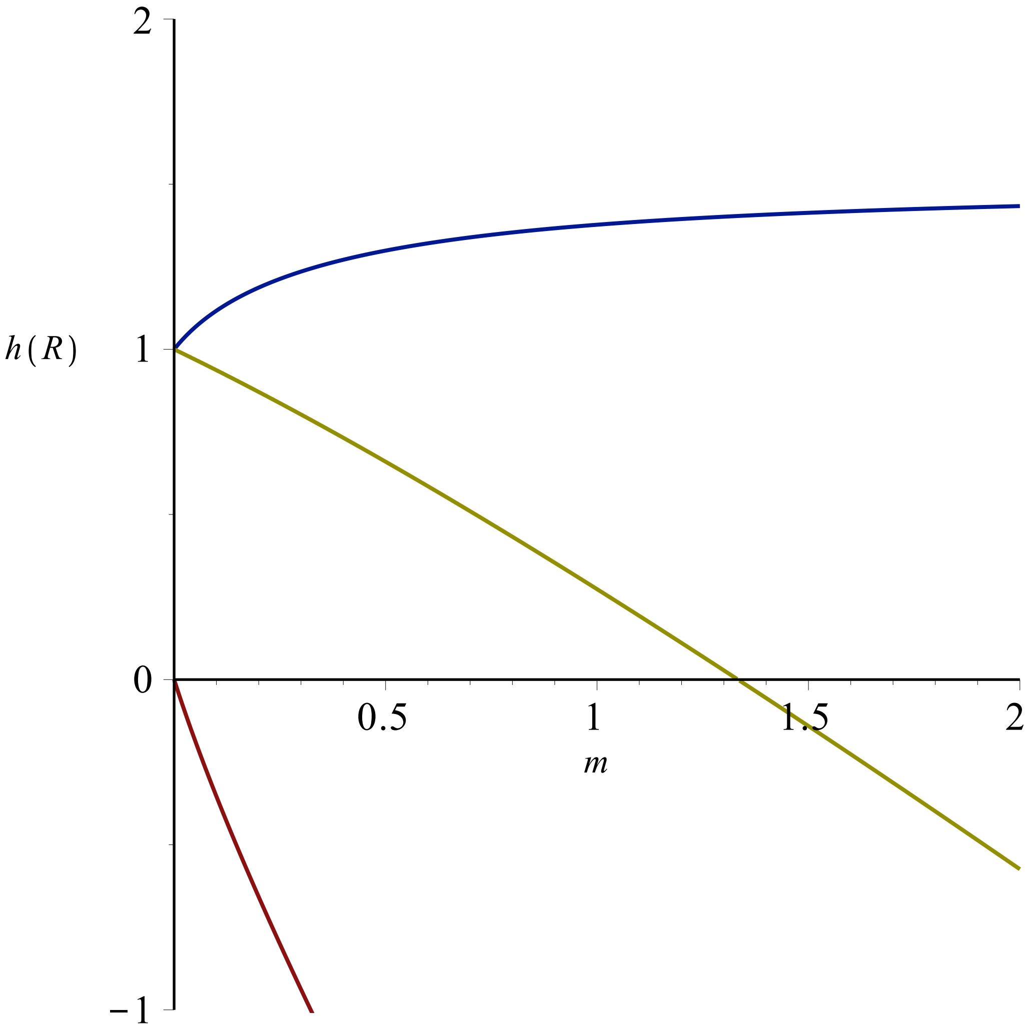

Because in equilibrium necessarily $h(R) > 1$ should hold, the above graph shows that the first solution for $h(R)$ is the only relevant one.

hvals[1]

$$\frac{1}{6}\left(-144\,m^2 - 108\,m - 8 + 12\sqrt{-12\,m^6 - 108\,m^5 - 192\,m^4 - 180\,m^3 - 15\,m^2}\right)^{1/3}$$

$$-\left(6\left(-\frac{1}{3}\,m^2 - m - \frac{1}{9}\right)\right)\Big/\left(-144\,m^2 - 108\,m - 8 + 12\sqrt{-12\,m^6 - 108\,m^5 - 192\,m^4 - 180}\right.$$

$$/3 \quad - m + \frac{2}{3}$$

simplify(*subs*(*rhs*(*op*(2, *sols*)) = $h(R)$, *op*(1, *sols*)))

$$q = \frac{1}{m\,(3\,m - 4)}\left(4\,m^4 + 6\,m^3\,h(R) + 2\,m^2\,h(R)^2 - 11\,m^3 - 10\,m^2\,h(R) - 3\,h(R)^2\,m + 10\,m^2 + 5\,m\,h(R) + h(R)^2 - 6\,m - 2\,h(R) + 1\right) \qquad \textbf{(2.45)}$$

The expression for the boundary of adult overcompensation can be derived by substituting the explicit expressions (2.44) for $h(R)$ into expression (2.45) for q. The resulting expression is ridiculously involved, but it is an explicit formula in terms of m only.

$Abnd := rhs(subs(rhs(op(2, sols)) = hvals[1], op(1, sols)));$

$$\frac{1}{m\,(3\,m-4)}\left(4\,m^4+6\,m^3\left(\frac{1}{6}\left(-144\,m^2-108\,m-8\right.\right.\right.$$

$$\left.+\,12\sqrt{-12\,m^6-108\,m^5-192\,m^4-180\,m^3-15\,m^2}\right)^{1/3}$$

$$-\left(6\left(-\frac{1}{3}\,m^2-m-\frac{1}{9}\right)\right)\Big/\left(-144\,m^2-108\,m-8+12\sqrt{-12\,m^6-108\,m^5-192\,m^4-180}\right.$$

$$^{/3}\left.-\,m+\frac{2}{3}\right)$$

$$+\,2\,m^2\left(\frac{1}{6}\left(-144\,m^2-108\,m-8\right.\right.$$

$$\left.+\,12\sqrt{-12\,m^6-108\,m^5-192\,m^4-180\,m^3-15\,m^2}\right)^{1/3}$$

$$-\left(6\left(-\frac{1}{3}\,m^2-m-\frac{1}{9}\right)\right)\Big/\left(-144\,m^2-108\,m-8+12\sqrt{-12\,m^6-108\,m^5-192\,m^4-180}\right.$$

$$^{/3}\left.-\,m+\frac{2}{3}\right)^2-11\,m^3-10\,m^2\left(\frac{1}{6}\left(-144\,m^2-108\,m-8\right.\right.$$

$$\left.+\,12\sqrt{-12\,m^6-108\,m^5-192\,m^4-180\,m^3-15\,m^2}\right)^{1/3}$$

$$-\left(6\left(-\frac{1}{3}\,m^2-m-\frac{1}{9}\right)\right)\Big/\left(-144\,m^2-108\,m-8+12\sqrt{-12\,m^6-108\,m^5-192\,m^4-180}\right.$$

$$^{/3}\left.-\,m+\frac{2}{3}\right)$$

$$-3\left(\frac{1}{6}\left(-144\,m^2-108\,m-8\right.\right.$$

$$\left.+12\sqrt{-12\,m^6-108\,m^5-192\,m^4-180\,m^3-15\,m^2}\right)^{1/3}$$

$$-\left(6\left(-\frac{1}{3}\,m^2-m-\frac{1}{9}\right)\right)\Big/\left(-144\,m^2-108\,m-8+12\sqrt{-12\,m^6-108\,m^5-192\,m^4-180}\right.$$

$$\left./3-m+\frac{2}{3}\right)^2 m+10\,m^2+5\,m\left(\frac{1}{6}\left(-144\,m^2-108\,m-8\right.\right.$$

$$\left.+12\sqrt{-12\,m^6-108\,m^5-192\,m^4-180\,m^3-15\,m^2}\right)^{1/3}$$

$$-\left(6\left(-\frac{1}{3}\,m^2-m-\frac{1}{9}\right)\right)\Big/\left(-144\,m^2-108\,m-8+12\sqrt{-12\,m^6-108\,m^5-192\,m^4-180}\right.$$

$$\left./3-m+\frac{2}{3}\right)$$

$$+\left(\frac{1}{6}\left(-144\,m^2-108\,m-8\right.\right.$$

$$\left.+12\sqrt{-12\,m^6-108\,m^5-192\,m^4-180\,m^3-15\,m^2}\right)^{1/3}$$

$$-\left(6\left(-\frac{1}{3}\,m^2-m-\frac{1}{9}\right)\right)\Big/\left(-144\,m^2-108\,m-8+12\sqrt{-12\,m^6-108\,m^5-192\,m^4-180}\right.$$

$$\left./3-m+\frac{2}{3}\right)^2-4\,m-\frac{1}{3}\left(-144\,m^2-108\,m-8\right.$$

$$+ 12\sqrt{-12\,m^6 - 108\,m^5 - 192\,m^4 - 180\,m^3 - 15\,m^2}\Big)^{1/3}$$

$$+ \left(12\left(-\frac{1}{3}\,m^2 - m - \frac{1}{9}\right)\right) \Big/ \Big(-144\,m^2 - 108\,m - 8 + 12\sqrt{-12\,m^6 - 108\,m^5 - 192\,m^4 - 18[illegible]}$$

$${}^{/3} - \frac{1}{3}\Big)$$

Below is the summarizing graph. Overcompensation only depends on 2 dimensionless ratios: $m = \frac{\mu}{T}$ and $q = \frac{\beta}{\gamma}$. Juvenile density increases with increasing mortality for parameter combinations below the blue curve, adult density increases with increasing mortality for parameter combinations above the red curve

$$plot\left([Jbnd, Abnd], m = 0..5, view = [0..1, 0..3], labels = \left[\frac{\mu}{T}, \frac{\beta}{\gamma}\right], color = [\text{"Navy"}, \text{"Burgundy"}]\right)$$

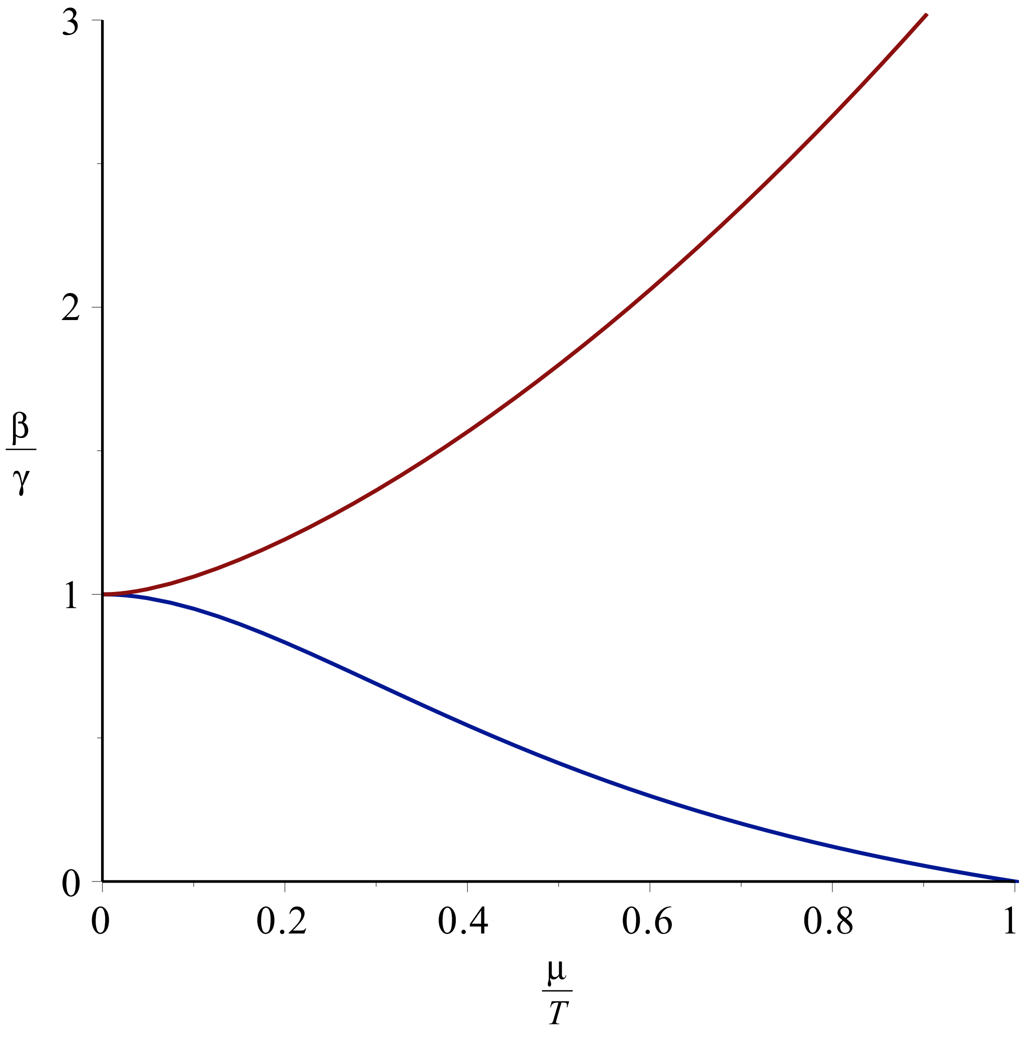

II.3: Stability

Now let's consider the stability of the non-trival equilibrim

$S := J - \langle\langle \lambda, 0, 0\rangle | \langle 0, \lambda, 0\rangle | \langle 0, 0, \lambda\rangle\rangle;$

$$\begin{bmatrix} -\left(\frac{d}{dR} f(R)\right) C_J - \left(\frac{d}{dR} f(R)\right) C_A - \lambda & -f(R) & -f(R) \\ \beta \left(\frac{d}{dR} f(R)\right) C_A - \gamma \left(\frac{d}{dR} f(R)\right) C_J & -\gamma f(R) + T - \mu - \lambda & \beta f(R) - T \\ \gamma \left(\frac{d}{dR} f(R)\right) C_J & \gamma f(R) - T & -\mu - \lambda \end{bmatrix} \quad \textbf{(3.1)}$$

$simplify(LinearAlgebra[Determinant](S), \{EquiCond0\});$

$$-\lambda^3 - C_A \left(\frac{d}{dR} f(R)\right) f(R)\, \gamma T - C_A \left(\frac{d}{dR} f(R)\right) f(R)\, \gamma \lambda - C_A \left(\frac{d}{dR} f(R)\right) f(R)\, \gamma \mu - C_A \left(\frac{d}{dR} f(R)\right) f(R)\, \beta \lambda - C_A \left(\frac{d}{dR} f(R)\right) f(R)\, \beta \mu - C_J \left(\frac{d}{dR} f(R)\right) f(R)\, \gamma \lambda - C_J \left(\frac{d}{dR} f(R)\right) f(R)\, \gamma \mu - C_J \left(\frac{d}{dR} f(R)\right) f(R)\, T \beta - C_J \left(\frac{d}{dR} f(R)\right) \lambda^2 - f(R)\, \gamma \lambda^2 + C_A \left(\frac{d}{dR} f(R)\right) T^2 - C_A \left(\frac{d}{dR} f(R)\right) \lambda^2 - C_A \left(\frac{d}{dR} f(R)\right) \mu^2 - C_J \left(\frac{d}{dR} f(R)\right) \mu^2 + C_J \left(\frac{d}{dR} f(R)\right) T^2 + T \lambda \mu + f(R)^2 \gamma \beta \lambda + C_A \left(\frac{d}{dR} f(R)\right) T \lambda + C_A \left(\frac{d}{dR} f(R)\right) T \mu - 2 C_A \left(\frac{d}{dR} f(R)\right) \lambda \mu + C_J \left(\frac{d}{dR} f(R)\right) T \lambda + C_J \left(\frac{d}{dR} f(R)\right) T \mu - 2 C_J \left(\frac{d}{dR} f(R)\right) \lambda \mu - f(R)\, \gamma T \lambda - f(R)\, \gamma \lambda \mu - f(R)\, T \beta \lambda + T^2 \lambda + T \lambda^2 - 2 \lambda^2 \mu - \lambda \mu^2 \quad \textbf{(3.2)}$$

$CE := collect\left(-\%, \left[\lambda, \frac{d}{dR} f(R), C_J, C_A\right]\right)$

$$\lambda^3 + \left((C_A + C_J) \left(\frac{d}{dR} f(R)\right) + \gamma f(R) - T + 2\mu\right) \lambda^2 + \left(\left((\gamma f(R) - T + 2\mu)\, C_J + (\gamma f(R) + \beta f(R) - T + 2\mu)\, C_A\right) \left(\frac{d}{dR} f(R)\right) - f(R)^2 \gamma \beta + f(R)\, \gamma T + f(R)\, \gamma \mu + f(R)\, T \beta - T^2 - T\mu + \mu^2\right) \lambda + \left(\left(f(R)\, \gamma \mu + f(R)\, T \beta - T^2 - T \mu + \mu^2\right) C_J + \left(f(R)\, \gamma T + f(R)\, \gamma \mu + f(R)\, \beta \mu - T^2 - T \mu + \mu^2\right) C_A\right) \left(\frac{d}{dR} f(R)\right) \quad \textbf{(3.3)}$$

$a_1 := (C_J + C_A) \left(\frac{d}{dR} f(R)\right) + (\gamma f(R) - T + 2 \cdot \mu)$

$$(C_A + C_J) \left(\frac{d}{dR} f(R)\right) + \gamma f(R) - T + 2\mu \quad \textbf{(3.4)}$$

$$a_2 := \left(\left(\gamma f(R) - T + 2\cdot\mu\right)\cdot\left(C_J + C_A\right) + \beta\cdot f(R)\cdot C_A\right)\left(\frac{d}{dR} f(R)\right)$$

$$\left(\left(\gamma f(R) - T + 2\,\mu\right)\left(C_A + C_J\right) + C_A f(R)\,\beta\right)\left(\frac{d}{dR} f(R)\right) \tag{3.5}$$

$$a_3 := \left(\left(T\cdot\left(\beta\cdot f(R) - T - \mu\right) + \mu\cdot\left(\gamma\cdot f(R) + \mu\right)\right)\cdot C_J + \left(T\cdot\left(\gamma\cdot f(R) - T\right) + \mu\cdot\left(\gamma\cdot f(R) - T + \mu + \beta\cdot f(R)\right)\right)\cdot C_A\right)\left(\frac{d}{dR} f(R)\right)$$

$$\left(\left(T\left(\beta f(R) - T - \mu\right) + \mu\left(\gamma f(R) + \mu\right)\right) C_J + \left(T\left(\gamma f(R) - T\right) + \mu\left(\gamma f(R) + \beta f(R) - T + \mu\right)\right) C_A\right)\left(\frac{d}{dR} f(R)\right) \tag{3.6}$$

$$simplify\left(\lambda^3 + a_1\cdot\lambda^2 + a_2\cdot\lambda + a_3 - CE, \{EquiCond\}\right);$$

$$0 \tag{3.7}$$

The Routh-Hurwitz criteria stipulate that the equilibrium is stable if $a_1 > 0$, $a_3 > 0$ and $a_1\cdot a_2 > a_3$. Clearly, the first two conditions are satisfied, given that we assume $\left(\frac{d}{dR} f(R)\right) > 0$ and it was deduced above that $\beta f(R) - T - \mu > 0$ and $\gamma\cdot f(R) - T > 0$.

Check the last condition

$$RH3a := collect\left(simplify\left(a_1\cdot a_2 - a_3, \{EquiCond\}\right), \left[\frac{d}{dR} f(R), C_J, C_A\right]\right);$$

$$\left(\left(\gamma f(R) - T + 2\,\mu\right) C_J^2 + \left(2\,\gamma f(R) + \beta f(R) - 2\,T + 4\,\mu\right) C_A C_J + \left(\gamma f(R) + \beta f(R) - T + 2\,\mu\right) C_A^2\right)\left(\frac{d}{dR} f(R)\right)^2 + \left(\left(\gamma^2 f(R)^2 - 2 f(R)\,\gamma\,T + 3 f(R)\,\gamma\,\mu - f(R)\,T\,\beta + 2\,T^2 - 3\,T\,\mu + 3\,\mu^2\right) C_J + \left(\gamma^2 f(R)^2 - 2 f(R)\,\gamma\,T + 4 f(R)\,\gamma\,\mu + f(R)\,\beta\,\mu + T^2 - 4\,T\,\mu + 4\,\mu^2\right) C_A\right)\left(\frac{d}{dR} f(R)\right) \tag{3.8}$$

The two terms in this expression share a common factor $\left(\frac{d}{dR} f(R)\right)$ which we can drop as it is a positive term. Also rearrange the expression.

$$\left(\left(\gamma\cdot f(R) - T + 2\cdot\mu\right)\cdot C_J^2 + \left(\left(\gamma\cdot f(R) - T + 2\cdot\mu\right) + \left(\gamma\cdot f(R) + \beta\cdot f(R) - T + 2\cdot\mu\right)\right)\cdot C_J\cdot C_A + \left(\gamma\cdot f(R) + \beta\cdot f(R) - T + 2\cdot\mu\right)\cdot C_A^2\right)\cdot\left(\frac{d}{dR} f(R)\right) + \left(\left(\left(\gamma\cdot f(R) - T + \mu\right)^2 - T\cdot\left(\beta\cdot f(R) - T\right) + \mu\cdot\left(\gamma\cdot f(R) - T + 2\cdot\mu\right)\right)\cdot C_J + \left(\left(\gamma\cdot f(R) - T + 2\cdot\mu\right)^2 + \mu\cdot\beta\cdot f(R)\right)\cdot C_A\right)$$

$$\left(\left(\gamma f(R) - T + 2\,\mu\right) C_J^2 + \left(2\,\gamma f(R) + \beta f(R) - 2\,T + 4\,\mu\right) C_A C_J + \left(\gamma f(R) + \beta f(R) - T + 2\,\mu\right) C_A^2\right)\left(\frac{d}{dR} f(R)\right) + \left(\left(\gamma f(R) - T + \mu\right)^2 - T\left(\beta f(R) - T\right) + \mu\left(\gamma f(R) - T + 2\,\mu\right)\right) C_J + \left(\left(\gamma f(R) - T + 2\,\mu\right)^2 + f(R)\,\beta\,\mu\right) C_A \tag{3.9}$$

$$simplify\left(\left(\frac{d}{dR} f(R)\right)\cdot\% - RH3a\right);$$

$$0 \tag{3.10}$$

Now substitute the relation between adult and juvenile density at equilibrium $C_A = \frac{(\gamma f(R) - T)}{\mu}\cdot C_J$ to eliminate C_A from the expression.

$$collect\left(\left(subs\left(C_A = \frac{(\gamma f(R) - T)}{\mu}\cdot C_J, \%\%\right)\right), \left[\frac{d}{dR} f(R), C_J\right]\right);$$

$$\left(\gamma f(R) - T + 2\mu + \frac{(2\gamma f(R) + \beta f(R) - 2T + 4\mu)(\gamma f(R) - T)}{\mu} + \frac{(\gamma f(R) + \beta f(R) - T + 2\mu)(\gamma f(R) - T)^2}{\mu^2}\right) C_J^2 \left(\frac{d}{dR} f(R)\right) + \left((\gamma f(R) - T + \mu)^2 - T(\beta f(R) - T) + \mu(\gamma f(R) - T + 2\mu) + \frac{((\gamma f(R) - T + 2\mu)^2 + f(R)\beta\mu)(\gamma f(R) - T)}{\mu}\right) C_J \tag{3.11}$$

This allows for dropping another a factor, C_J, from the expression.

$$\left(\gamma f(R) - T + 2\mu + \frac{(2\gamma f(R) + \beta f(R) - 2T + 4\mu)(\gamma f(R) - T)}{\mu} + \frac{(\gamma f(R) + \beta f(R) - T + 2\mu)(\gamma f(R) - T)^2}{\mu^2}\right) C_J \left(\frac{d}{dR} f(R)\right) + \left((\gamma f(R) - T + \mu)^2 - T(\beta f(R) - T) + \mu(\gamma f(R) - T + 2\mu) + \frac{((\gamma f(R) - T + 2\mu)^2 + \mu\beta f(R))(\gamma f(R) - T)}{\mu}\right)$$

$$\left(\gamma f(R) - T + 2\mu + \frac{(2\gamma f(R) + \beta f(R) - 2T + 4\mu)(\gamma f(R) - T)}{\mu} + \frac{(\gamma f(R) + \beta f(R) - T + 2\mu)(\gamma f(R) - T)^2}{\mu^2}\right) C_J \left(\frac{d}{dR} f(R)\right) + (\gamma f(R) - T + \mu)^2 - T(\beta f(R) - T) + \mu(\gamma f(R) - T + 2\mu) + \frac{((\gamma f(R) - T + 2\mu)^2 + f(R)\beta\mu)(\gamma f(R) - T)}{\mu} \tag{3.12}$$

$$simplify\left(\left(\frac{d}{dR} f(R)\right)\cdot C_J\cdot\% - RH3a, \left\{C_A = \frac{(\gamma f(R) - T)}{\mu}\cdot C_J\right\}\right);$$

$$0 \tag{3.13}$$

$$subs(C_J = Jeq, \%\%);$$

$$\frac{1}{f(R)\,(\gamma f(R)-T+\mu)}\Bigg(\Bigg(\gamma f(R)-T+2\mu + \frac{(2\gamma f(R)+\beta f(R)-2T+4\mu)\,(\gamma f(R)-T)}{\mu} + \frac{(\gamma f(R)+\beta f(R)-T+2\mu)\,(\gamma f(R)-T)^2}{\mu^2}\Bigg)P\mu\left(\frac{d}{dR}f(R)\right)\Bigg) + (\gamma f(R)-T+\mu)^2 - T(\beta f(R)-T) + \mu(\gamma f(R)-T+2\mu) + \frac{((\gamma f(R)-T+2\mu)^2+f(R)\beta\mu)\,(\gamma f(R)-T)}{\mu} \quad \textbf{(3.14)}$$

$$b_1 := \frac{\mu\cdot(\gamma f(R)-T+2\mu)}{f(R)\cdot(\gamma f(R)-T+\mu)} + \frac{(2\gamma f(R)+\beta f(R)-2T+4\mu)\,(\gamma f(R)-T)}{f(R)\cdot(\gamma f(R)-T+\mu)} + \frac{(\gamma f(R)+\beta f(R)-T+2\mu)\,(\gamma f(R)-T)^2}{\mu\cdot f(R)\cdot(\gamma f(R)-T+\mu)}$$

$$\frac{\mu\,(\gamma f(R)-T+2\mu)}{f(R)\,(\gamma f(R)-T+\mu)} + \frac{(2\gamma f(R)+\beta f(R)-2T+4\mu)\,(\gamma f(R)-T)}{f(R)\,(\gamma f(R)-T+\mu)} + \frac{(\gamma f(R)+\beta f(R)-T+2\mu)\,(\gamma f(R)-T)^2}{\mu f(R)\,(\gamma f(R)-T+\mu)} \quad \textbf{(3.15)}$$

$$b_2 := (\gamma f(R)-T+\mu)^2 - T(\beta f(R)-T) + \mu(\gamma f(R)-T+2\mu) + \frac{((\gamma f(R)-T+2\mu)^2+\mu\beta f(R))\,(\gamma f(R)-T)}{\mu}$$

$$(\gamma f(R)-T+\mu)^2 - T(\beta f(R)-T) + \mu(\gamma f(R)-T+2\mu) + \frac{((\gamma f(R)-T+2\mu)^2+f(R)\beta\mu)\,(\gamma f(R)-T)}{\mu} \quad \textbf{(3.16)}$$

$$simplify\left(\left(\frac{d}{dR}f(R)\right)\cdot C_J\cdot\left(b_1\cdot P\cdot\left(\frac{d}{dR}f(R)\right)+b_2\right)-RH3a, \left\{C_A=\frac{(\gamma f(R)-T)}{\mu}\cdot C_J, C_J = Jeq\right\}\right);$$

$$0 \quad \textbf{(3.17)}$$

Therefore, the equilibrium is unstable if $b_1\cdot P\cdot\left(\frac{d}{dR}f(R)\right)+b_2 < 0$ and stable if $b_1\cdot P\cdot\left(\frac{d}{dR}f(R)\right)+b_2 > 0$. Because $\beta f(R)-T > 0$ and $\gamma f(R)-T > 0$ it can easily be seen that all terms in the coefficient b_1 are positive. Therefore, the equilibrium can only be unstable if $b_2 < 0$. Inspection of the expression for b_2 reveals that b_2 can only become negative if the term $-T(\beta f(R)-T)$ is substantially negative, because all other terms in the expression for b_2 are positive. Furthermore, if $b_2 < 0$ the equilibrium will be unstable for values of P that are positive but close to 0. Increasing the resource productivity P will then result in the equilibrium turning stable, since

$$b_1 > 0 \text{ and } \left(\frac{d}{dR} f(R)\right) > 0.$$

Rearrange the expressions for b_1 and b_2:

$$b_1 := \frac{3\cdot(\beta f(R) - T)\cdot\mu + 4\cdot(\beta f(R) - T)\cdot(\gamma f(R) - T) + \gamma\cdot f(R)\cdot(\gamma\cdot f(R) - T + \mu)}{\gamma\cdot f(R)\cdot(\beta\cdot f(R) - T)}$$

$$\frac{3\,(\beta f(R) - T)\,\mu + 4\,(\beta f(R) - T)\,(\gamma f(R) - T) + \gamma f(R)\,(\gamma f(R) - T + \mu)}{\gamma f(R)\,(\beta f(R) - T)} \tag{3.18}$$

$$b_2 := (\gamma f(R) - T + \mu)^2 - T\,(\beta f(R) - T) + \mu\,(\gamma f(R) - T + 2\mu) + \frac{\left((\gamma f(R) - T + 2\mu)^2 + \mu\,\beta f(R)\right)(\gamma f(R) - T)}{\mu}$$

$$(\gamma f(R) - T + \mu)^2 - T\,(\beta f(R) - T) + \mu\,(\gamma f(R) - T + 2\mu) + \frac{\left((\gamma f(R) - T + 2\mu)^2 + f(R)\,\beta\,\mu\right)(\gamma f(R) - T)}{\mu} \tag{3.19}$$

$$simplify\left(\left(\frac{d}{dR} f(R)\right)\cdot C_J\cdot\left(b_1\cdot\gamma\cdot P\cdot\left(\frac{d}{dR} f(R)\right) + b_2\right) - RH3a, \left\{C_A = \frac{(\gamma f(R) - T)}{\mu}\cdot C_J, C_J = Jeq, EquiCond\right\}\right);$$

$$0 \tag{3.20}$$

Now express the expressions for the coefficients b_1 and b_2 in terms of the scaled parameters $m = \frac{\mu}{T}$ and $q = \frac{\beta}{\gamma}$ and in terms of the scaled function $h(R) = \frac{\gamma}{T}\cdot f(R)$:

$$collect\left(simplify\left(subs\left(\beta\cdot f(R) = q\cdot h(R)\cdot T, \mu = m\cdot T, \gamma\cdot f(R) = h(R)\cdot T, b_1\right)\right), [T]\right)$$

$$\frac{(3\,(q\,h(R) - 1)\,m + 4\,(q\,h(R) - 1)\,(h(R) - 1))\,T}{\gamma f(R)\,(q\,h(R) - 1)} + \frac{h(R) - 1 + m}{q\,h(R) - 1} \tag{3.21}$$

$$c_1 := \frac{(3\,(q\,h(R)-1)\,m + 4\,(q\,h(R)-1)\,(h(R)-1)) + h(R)\cdot(m + h(R)-1)}{h(R)\,(q\,h(R)-1)}$$

$$\frac{3\,(q\,h(R) - 1)\,m + 4\,(q\,h(R) - 1)\,(h(R) - 1) + h(R)\,(h(R) - 1 + m)}{h(R)\,(q\,h(R) - 1)} \tag{3.22}$$

$$collect\left(simplify\left(subs\left(\beta\cdot f(R) = q\cdot h(R)\cdot T, \mu = m\cdot T, \gamma\cdot f(R) = h(R)\cdot T, b_2\right)\right), [T]\right)$$

$$\left((h(R) - 1 + m)^2 - q\,h(R) + 1 + m\,(h(R) + 2m - 1) + \frac{(h(R) + 2m - 1)^2\,(h(R) - 1)}{m}\right)T^2 + f(R)\,\beta\,(h(R) - 1)\,T \tag{3.23}$$

$$c_2 := (m + h(R)-1)^2 - q\,h(R) + 1 + m\,(2m + h(R)-1) + \frac{(2m + h(R)-1)^2\,(h(R)-1)}{m} + q\cdot h(R)\cdot(h(R)-1)$$

$$(h(R) - 1 + m)^2 - q\,h(R) + 1 + m\,(h(R) + 2m - 1) \tag{3.24}$$

$$+\frac{(h(R)+2m-1)^2\,(h(R)-1)}{m}+q\,h(R)\,(h(R)-1)$$

$$simplify\left(\left(\frac{d}{dR}f(R)\right)\cdot C_J\cdot\left(subs\left(q=\frac{\beta}{\gamma}, m=\frac{\mu}{T}, h(R)=\frac{\gamma}{T}\cdot f(R), c_1\cdot\left(\gamma\cdot P\cdot\left(\frac{d}{dR}f(R)\right)\right)\right.\right.\right.$$
$$\left.\left.\left.+c_2\cdot T^2\right)\right)\text{-}RH3a, \left\{C_A=\frac{(\gamma f(R)-T)}{\mu}\cdot C_J, C_J=Jeq, EquiCond\right\}\right)$$

$$0 \qquad \textbf{(3.25)}$$

The equilibrium is therefore stable if:

$$\frac{\gamma\cdot P}{T^2}\cdot\left(\frac{d}{dR}f(R)\right)>\text{-}\left(\frac{c_2}{c_1}\right)$$

with the coefficients c_1 and c_2 given by:

$c_1; c_2$

$$\frac{3\,(q\,h(R)-1)\,m+4\,(q\,h(R)-1)\,(h(R)-1)+h(R)\,(h(R)-1+m)}{h(R)\,(q\,h(R)-1)}$$

$$(h(R)-1+m)^2-q\,h(R)+1+m\,(h(R)+2m-1) \qquad \textbf{(3.26)}$$
$$+\frac{(h(R)+2m-1)^2\,(h(R)-1)}{m}+q\,h(R)\,(h(R)-1)$$

As was argued before, the quantity $\frac{\gamma\cdot P}{T^2}\cdot\left(\frac{d}{dR}f(R)\right)$ scales with resource productivity P and can therefore adopt any value, by choosing an appropriate P. Furthermore, $c_1 > 0$ so the only thing to resolve is to see for which values of m and q the coefficient c_2 is negative.

First plot the value of c_2 as a function of m and q:

$$plot3d\left(subs\left(h(R)=hequi, c_2\right), m=0..1, q=0..3, view=[0..1, 0..3, 0..5], labels=\left[\frac{\mu}{T}, \frac{\beta}{\gamma},\right.\right.$$
$$\left.\left.\text{'}c_2\text{'}\right], orientation=[40, 80, \text{-}8]\right)$$

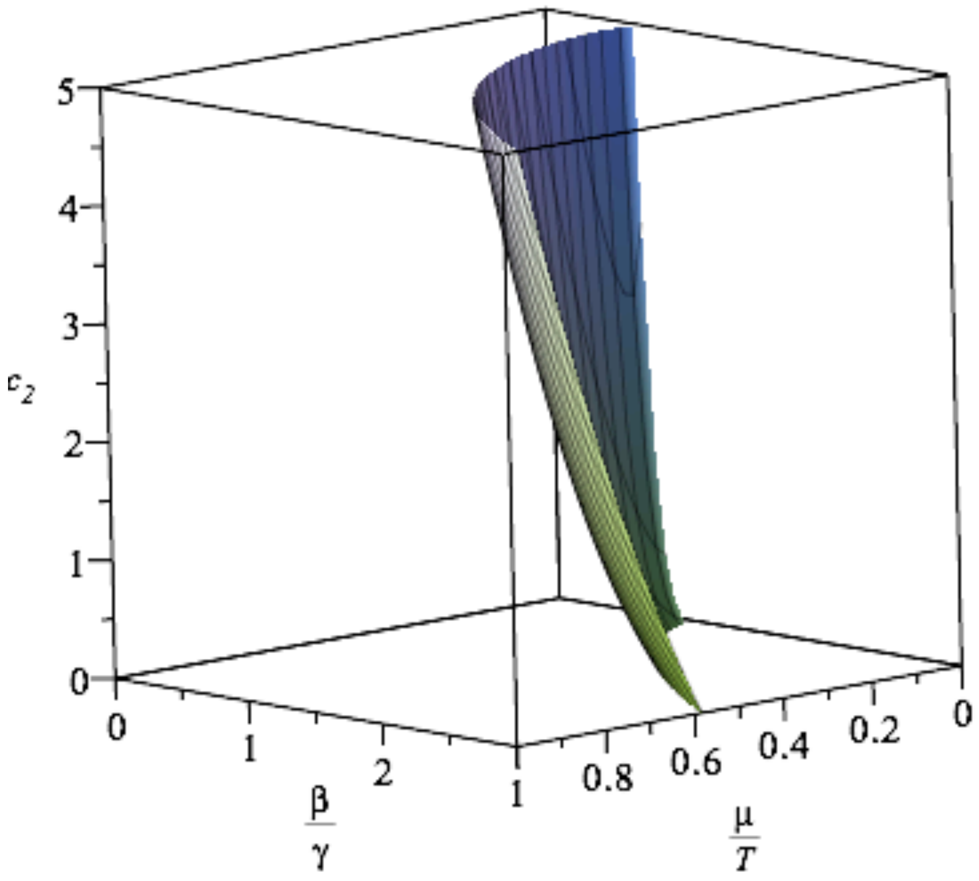

This shows that the equilibrium can only be unstable for low values of $m = \frac{\mu}{T}$ and high values of $q = \frac{\beta}{\gamma}$.

Now solve for the values of m and q where the coefficient c_2 is exactly 0.

$Stbbnd := solve\left(subs\left(h(R) = hequi, c_2\right), q\right)$ assuming m :: *positive*, q :: *positive*;

$$RootOf\left(\left(5\,m^3 - 9\,m^2 + 4\,m - 1\right)\_Z^4 + \left(-16\,m^5 + 20\,m^4 + m^3 + 8\,m^2 - 10\,m + 4\right)\_Z^3 + \left(-8\,m^5 - 15\,m^4 + 15\,m^3 + 5\,m^2 + 5\,m - 6\right)\_Z^2 + \left(-m^5 - 5\,m^4 - 18\,m^3 + 2\,m^2 + 4\,m + 4\right)\_Z - 3\,m^3 - 6\,m^2 - 3\,m - 1\right) \quad \textbf{(3.27)}$$

This does not give an explicit expression, but can nonetheless be plotted easily by Maple.

Below the boundaries of overcompensation in juvenile (blue) and adult density (red) with an increase in mortality are shown, together with the boundary (black, dashed) above which population cycles can occur at low resource productivity.

$plot\left(\left[Jbnd, Abnd, Stbbnd\right], m = 0..5, view = \left[0..1, 0..3\right], labels = \left[\frac{\mu}{T}, \frac{\beta}{\gamma}\right], linestyle = \left[solid, solid, dash\right], color = \left["Navy", "Burgundy", "Black"\right]\right)$

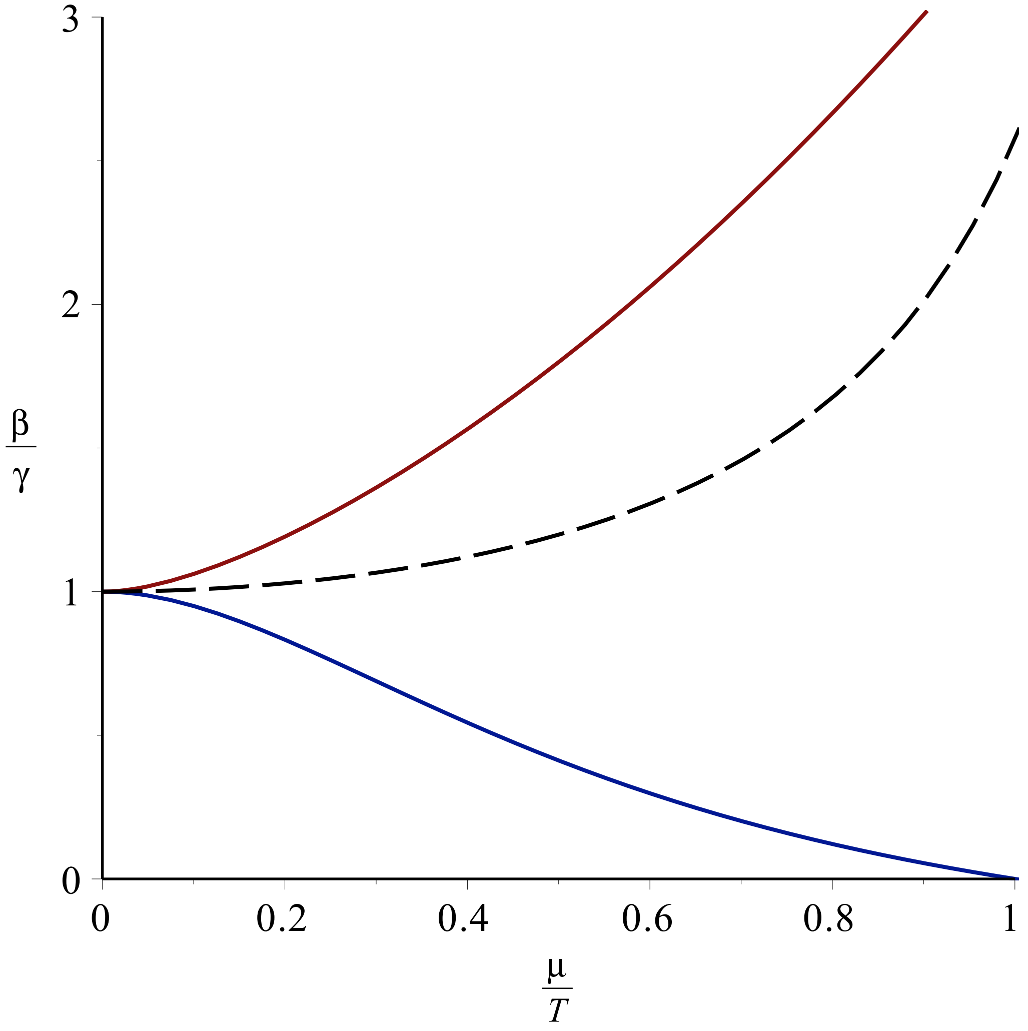

$$with(plots) : plot3d\left(subs\left(h(R) = hequi, -\frac{c_2}{c_1}\right), m = 0..0.6, q = 1..3, labels = \left[\frac{\mu}{T}, \frac{\beta}{\gamma}, \text{""}\right],\right.$$

$$view = [0..0.6, 1..3, 0..3], orientation = [40, 80, -3], labeldirections = [horizontal,$$

$$horizontal, vertical], title = typeset\left(\text{"Minimum value of ", } \frac{\gamma \cdot P}{T^2} \cdot f'(R),\right.$$

$$\left.\left.\text{" for equilibrium stability"}\right)\right)$$

Minimum value of $\frac{\gamma P \, \mathrm{D}(f)(R)}{T^2}$ for equilibrium stability

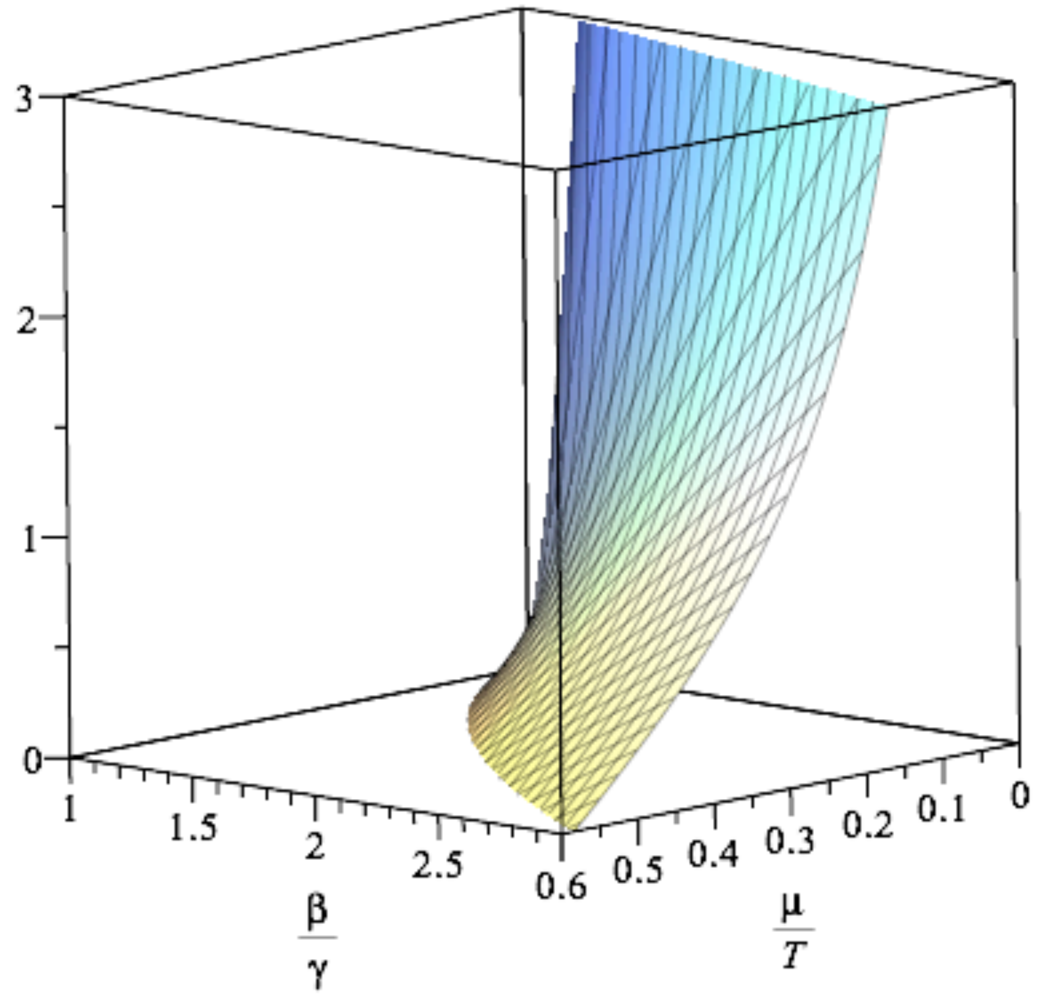

$$contourplot\left(subs\left(h(R) = hequi, -\frac{c_2}{c_1}\right), m = 0\,..0.6, q = 0\,..3, contours = [0, 1, 2, 3, 4, 5], grid = [100, 100], filledregions = true, coloring = [white, \text{"DarkGrey"}], labels = \left[\frac{\mu}{T}, \frac{\beta}{\gamma}\right]\right)$$

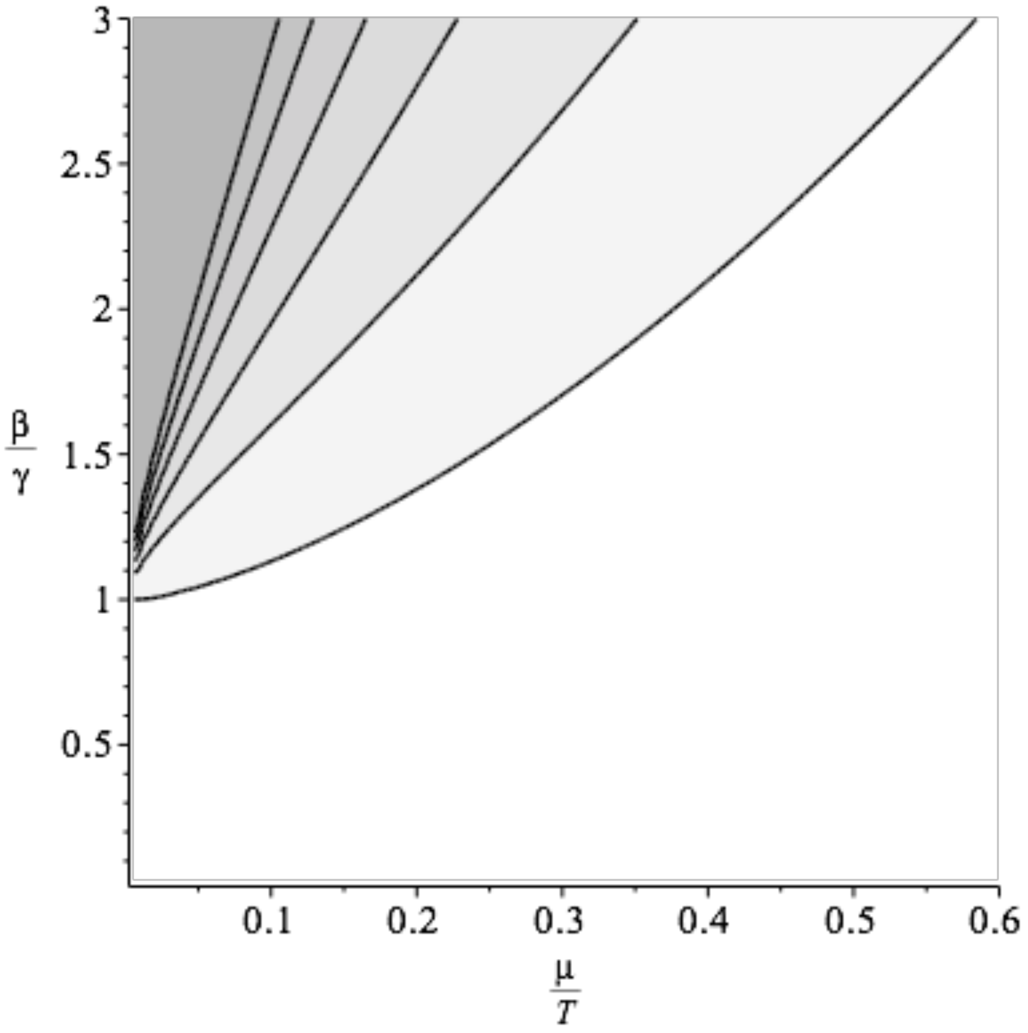

II.4: Increases in juvenile mortality only

To consider the effect of increases in juvenile and adult mortality separately, the mortality rates of juveniles and adults are now distinguished as μ_J and μ_A, respectively. The model is then described by the following set of ODEs:

$$dydt := \left[P - f(R)\cdot C_J - f(R)\cdot C_A,\ (\beta\cdot f(R) - T)\cdot C_A - (\gamma\cdot f(R) - T)\cdot C_J - \mu_J\cdot C_J,\ (\gamma\cdot f(R) - T)\cdot C_J - \mu_A\cdot C_A\right] : \left\langle\left\langle \frac{dR}{dt}, \frac{dC_J}{dt}, \frac{dC_A}{dt}\right\rangle\right\rangle = \langle\langle dydt\rangle\rangle;$$

$$\begin{bmatrix} \frac{dR}{dt} \\ \frac{dC_J}{dt} \\ \frac{dC_A}{dt} \end{bmatrix} = \begin{bmatrix} P - f(R)\, C_J - f(R)\, C_A \\ (\beta f(R) - T)\, C_A - (\gamma f(R) - T)\, C_J - \mu_J C_J \\ (\gamma f(R) - T)\, C_J - \mu_A C_A \end{bmatrix} \tag{4.1}$$

The equilibrium condition for the consumer, signifying R0 = 1 is now given by:

$$\frac{(\beta f(R) - T)\cdot(\gamma f(R) - T)}{\mu_A} - \left((\gamma f(R) - T) + \mu_J\right)$$

$$\frac{(\beta f(R) - T)\ (\gamma f(R) - T)}{\mu_A} - \gamma f(R) + T - \mu_J \qquad \textbf{(4.2)}$$

$$EquiCond2 := \left(\beta f(R) - T - \mu_A\right)\cdot(\gamma f(R) - T) - \mu_J\cdot\mu_A$$

$$\left(\beta f(R) - T - \mu_A\right)\ (\gamma f(R) - T) - \mu_J\,\mu_A \qquad \textbf{(4.3)}$$

$simplify\left(EquiCond2 \text{ - } \mu_A\cdot\%\%\right);$

$$0 \qquad \textbf{(4.4)}$$

Solve for the value of $f(R)$ at equilibrium:

$solve(EquiCond2, f(R))$

$$\frac{1}{2}\,\frac{1}{\gamma\beta}\Big(\mu_A\gamma + T\gamma + T\beta - \sqrt{\mu_A^{2}\gamma^{2} + 4\,\mu_A\,\mu_J\,\beta\,\gamma - 2\,\mu_A\,T\,\beta\,\gamma + 2\,\mu_A\,T\gamma^{2} + T^{2}\,\beta^{2} - 2\,T^{2}\,\beta\,\gamma + T^{2}\,\gamma^{2}}\Big), \frac{1}{2}\,\frac{1}{\gamma\beta}\Big(\mu_A\gamma + T\gamma + T\beta + \sqrt{\mu_A^{2}\gamma^{2} + 4\,\mu_A\,\mu_J\,\beta\,\gamma - 2\,\mu_A\,T\,\beta\,\gamma + 2\,\mu_A\,T\gamma^{2} + T^{2}\,\beta^{2} - 2\,T^{2}\,\beta\,\gamma + T^{2}\,\gamma^{2}}\Big) \qquad \textbf{(4.5)}$$

The second of these roots corresponds to the unique equilibrium value, for the same reasons as used above for the model with stage-independent mortality

$$Fequi2 := \frac{\left((\beta + \gamma)T + \gamma\cdot\mu_A + \mathrm{sqrt}\left(\left((\beta + \gamma)T + \gamma\cdot\mu_A\right)^2 - 4\cdot\beta\cdot\gamma\cdot\left(T^2 + T\cdot\mu_A - \mu_J\cdot\mu_A\right)\right)\right)}{2\cdot\beta\cdot\gamma}$$

$$\frac{1}{2}\,\frac{(\beta + \gamma)\ T + \mu_A\gamma + \sqrt{\left((\beta + \gamma)\ T + \mu_A\gamma\right)^2 - 4\,\beta\,\gamma\left(-\mu_A\,\mu_J + \mu_A\,T + T^2\right)}}{\gamma\beta} \qquad \textbf{(4.6)}$$

$simplify(Fequi2 - \%\%[2]);$

$$0 \qquad \textbf{(4.7)}$$

Now define $m_J = \frac{\mu_J}{T}$, $m_A = \frac{\mu_A}{T}$, $q = \frac{\beta}{\gamma}$, and $h(R) = \frac{\gamma\cdot f(R)}{T}$. Then, the equilibrium condition can be written as:

$simplify\left(subs\left(\beta = q\cdot\gamma\cdot T, \mu_J = m_J\cdot T, \mu_A = m_A\cdot T, Fequi2\right)\right)$

$$\frac{1}{2}\,\frac{1}{\gamma\,q\,T}\Big(T^2\,q + m_A\,T + \sqrt{T^2\left(T^2\,q^2 + 4\,T\,m_A\,m_J\,q - 2\,T\,m_A\,q - 2\,T\,q + m_A^{2} + 2\,m_A + 1\right)} + T\Big) \qquad \textbf{(4.8)}$$

And the value of $h(R) = \frac{\gamma\cdot f(R)}{T}$ at equilibrium is given by:

$$hequi2 := \frac{\left(m_A + q + 1 + \sqrt{(m_A + q + 1)^2 - 4 \cdot q \cdot (1 + m_A - m_J \cdot m_A)}\right)}{2 \cdot q}$$

$$\frac{1}{2} \frac{m_A + q + 1 + \sqrt{(m_A + q + 1)^2 - 4\, q\, (-m_A m_J + m_A + 1)}}{q} \quad \textbf{(4.9)}$$

To determine overcompensation, compute the Jacobian for use in the implicit function theorem equation.

$$Jy := VectorCalculus[Jacobian](dydt, [R, C_J, C_A]);$$

$$\begin{bmatrix} -\left(\frac{d}{dR} f(R)\right) C_J - \left(\frac{d}{dR} f(R)\right) C_A & -f(R) & -f(R) \\ \beta \left(\frac{d}{dR} f(R)\right) C_A - \gamma \left(\frac{d}{dR} f(R)\right) C_J & -\gamma f(R) + T - \mu_J & \beta f(R) - T \\ \gamma \left(\frac{d}{dR} f(R)\right) C_J & \gamma f(R) - T & -\mu_A \end{bmatrix} \quad \textbf{(4.10)}$$

Determine its determinant

$$DetJy0 := collect\left(LinearAlgebra[Determinant](Jy), \left[\frac{d}{dR} f(R), C_J, C_A\right]\right);$$

$$\left(\left(-\mu_J f(R)\, \gamma - f(R)\, T\, \beta - \mu_J \mu_A + T \mu_A + T^2\right) C_J + \left(-\gamma f(R)\, \mu_A - \mu_A f(R)\, \beta - f(R)\, \gamma\, T - \mu_J \mu_A + T \mu_A + T^2\right) C_A\right) \left(\frac{d}{dR} f(R)\right) \quad \textbf{(4.11)}$$

$$DetJy1 := -\left(\left(\mu_J \cdot (\gamma \cdot f(R) + \mu_A) + T \cdot (\beta \cdot f(R) - T - \mu_A)\right) \cdot C_J + \left(T \cdot (\gamma \cdot f(R) - T) + \mu_A \cdot (\gamma \cdot f(R) + \beta \cdot f(R) - T + \mu_J)\right) \cdot C_A\right) \cdot \left(\frac{d}{dR} f(R)\right)$$

$$-\left(\left(\mu_J (\gamma f(R) + \mu_A) + T (\beta f(R) - T - \mu_A)\right) C_J + \left(T (\gamma f(R) - T) + \mu_A (\gamma f(R) + \beta f(R) - T + \mu_J)\right) C_A\right) \left(\frac{d}{dR} f(R)\right) \quad \textbf{(4.12)}$$

$$simplify(DetJy1 - DetJy0);$$

$$0 \quad \textbf{(4.13)}$$

The determinant is negative (as before). To determine overcompensation with respect to an increase in juvenile mortality the following system of equations is solved:

$$J \cdot \left[\frac{dR}{d\mu_J}, \frac{dC_J}{d\mu_J}, \frac{dC_A}{d\mu_J}\right]^T - [0, C_J, 0]^T = 0$$

in which J is the Jacobian matrix of the system of ODEs. The solution $\left[\frac{dR}{d\mu_J}, \frac{dC_J}{d\mu_J}, \frac{dC_A}{d\mu_J}\right]$ is multiplied with -D, where D is the determinant of J, to simplify the expressions of the derivatives, but nonetheless keep the sign of the derivatives.

$$dydmuJ := simplify(-DetJy0 \cdot LinearAlgebra[LinearSolve](Jy, \langle 0, C_J, 0 \rangle));$$

$$\left[\begin{array}{c} f(R)\left(\gamma f(R)+\mu_A-T\right)C_J \\ -\left(\frac{d}{dR}f(R)\right)\left(C_J f(R)\gamma+\mu_A C_A+\mu_A C_J\right)C_J \\ -\left(\frac{d}{dR}f(R)\right)C_J\left(C_A f(R)\gamma-C_A T-C_J T\right) \end{array}\right] \quad \textbf{(4.14)}$$

$dCJdmuJ := collect\left(dydmuJ[2], \left[\frac{d}{dR}\,f(R), C_J, C_A\right]\right);$

$$\left(\left(-\gamma f(R)-\mu_A\right)C_J^2-\mu_A C_A C_J\right)\left(\frac{d}{dR}f(R)\right) \quad \textbf{(4.15)}$$

$dCJdmuJ := -\left(\left(\gamma f(R)+\mu_A\right)C_J+\mu_A\cdot C_A\right)\cdot C_J\cdot\left(\frac{d}{dR}\,f(R)\right); simplify(dCJdmuJ-\%\%);$

$$-\left(\left(\gamma f(R)+\mu_A\right)C_J+\mu_A C_A\right)C_J\left(\frac{d}{dR}f(R)\right)$$

$$0 \quad \textbf{(4.16)}$$

Clearly the derivative $\frac{dC_J}{d\mu_J}$ is always negative.

Thus, an increase in juvenile density can not occur with an increase in juvenile mortality alone. As a consequence, an emergent Allee effect can not occur for a predator that specialises on juvenile consumers.

Elsewhere, I have shown that this conclusion holds more generally, when juveniles and adults have different feeding rates, $\alpha_J\cdot f_J(R)$ and $\alpha_A\cdot f_A(R)$, different mortality rates, $\mu+\mu_J$ and $\mu+\mu_A$, maturation and reproduction rates equal to $\gamma\cdot f_J(R)-T_J$ and $\beta\cdot f_A(R)-T_A$, respectively, and a resource-dependent growth function $p(R)$ as long as $p'(R)\leq 0$.

Now consider a potential increase in adult density with increasing juvenile mortality:

$dCAdmuJ := collect\left(dydmuJ[3], \left[\frac{d}{dR}\,f(R), C_J, C_A\right]\right);$

$$\left(C_J^2 T+\left(-\gamma f(R)+T\right)C_A C_J\right)\left(\frac{d}{dR}f(R)\right) \quad \textbf{(4.17)}$$

$subs\left(C_A=\frac{(\gamma f(R)-T)}{\mu_A}\cdot C_J, dCAdmuJ\right)$

$$\left(C_J^2 T+\frac{\left(-\gamma f(R)+T\right)\left(\gamma f(R)-T\right)C_J^2}{\mu_A}\right)\left(\frac{d}{dR}f(R)\right) \quad \textbf{(4.18)}$$

Adult density hence increases with an increase in juvenile mortality if the following inequality holds:

$\mu_A\cdot T > (\gamma f(R)-T)^2$

$$\left(\gamma f(R)-T\right)^2 < T\mu_A \quad \textbf{(4.19)}$$

Rewrite the condition for adult overcompensation in terms of the scaled parameters $m_J = \frac{\mu_J}{T}$, $m_A = \frac{\mu_A}{T}$, $q = \frac{\beta}{\gamma}$, and $h(R) = \frac{\gamma \cdot f(R)}{T}$ and solve the condition in conjuction with the expression for the value of $h(R)$ in equilibrium.

$AincreaseJ := (h(R)-1)^2 - m_A$

$$(h(R)-1)^2 - m_A \tag{4.20}$$

$solsmuJ := solve(subs(h(R) = hequi2, AincreaseJ), q)$

$$\frac{m_J m_A + \sqrt{m_A^3 - 2\,m_A^2\,m_J + m_A m_J^2 + 2\,m_A^2 - 2\,m_A m_J + m_A} - m_A - 1}{m_A - 1}, \tag{4.21}$$
$$-\frac{-m_J m_A + \sqrt{m_A^3 - 2\,m_A^2\,m_J + m_A m_J^2 + 2\,m_A^2 - 2\,m_A m_J + m_A} + m_A + 1}{m_A - 1}$$

There are 2 possible roots, but the second one is strictly negative as can be shown by graphical inspection.

Plot the first root for $q = \frac{\beta}{\gamma}$ as a function of the scaled parameters $m_J = \frac{\mu_J}{T}$ and $m_A = \frac{\mu_A}{T}$. Adult density increases with an increase in juvenile mortality if the parameters are in the parameter space above the colored surface in the 3-dimensional graph below. Notice that the surface does extend below the value $q = \frac{\beta}{\gamma} = 1$, indicating that adult density can increase even if adults are less efficient than juveniles.

$plot3d\left(solsmuJ[1], m_J = 0..0.9999, m_A = 0..0.9999, labels = \left[\frac{\mu_J}{T}, \frac{\mu_A}{T}, \frac{\beta}{\gamma}\right], orientation = [147, 80, -3]\right);$

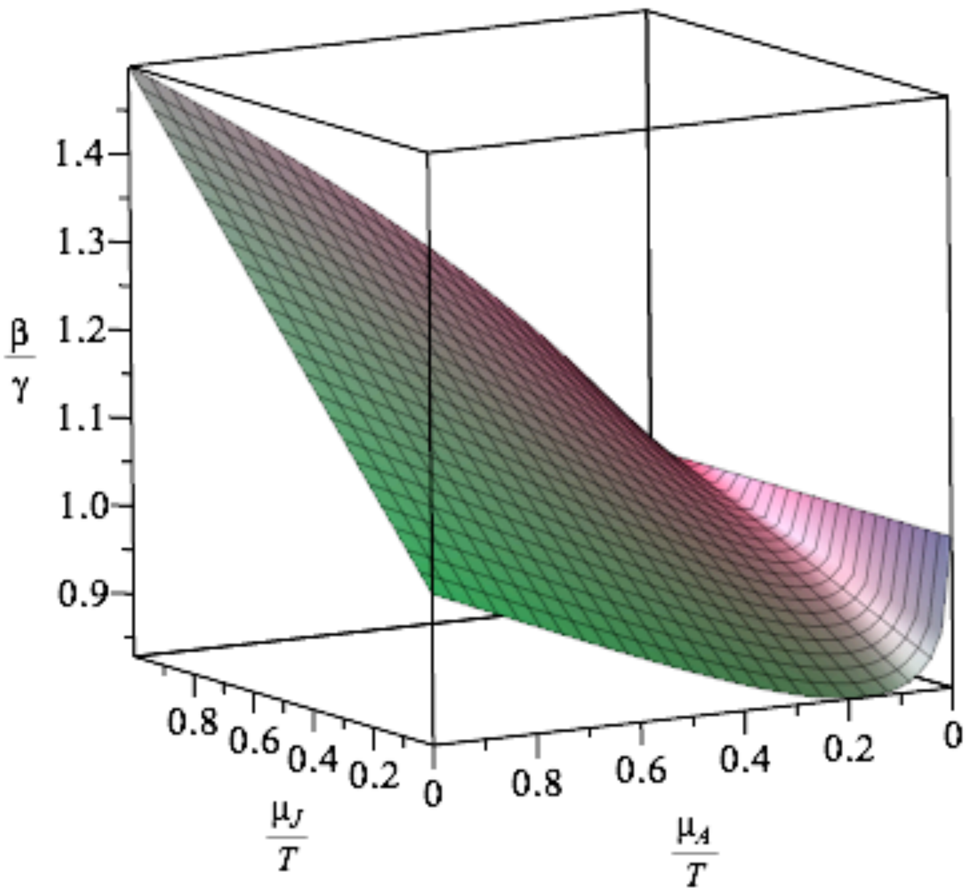

II.5: Increases in adult mortality only

To determine overcompensation with respect to an increase in adult mortality the following system of equations is solved:

$$J\cdot\left[\frac{dR}{d\mu_A}, \frac{dC_J}{d\mu_A}, \frac{dC_A}{d\mu_A}\right]^T - \left[0, 0, C_A\right]^T = 0$$

in which J is the Jacobian matrix of the system of ODEs. The solution $\left[\frac{dR}{d\mu_A}, \frac{dC_J}{d\mu_A}, \frac{dC_A}{d\mu_A}\right]$ is as before multiplied with -D, where D is the determinant of J, to simplify the expressions of the derivatives, but nonetheless keep the sign of the derivatives.

$$dydmuA := simplify\left(-DetJy0\cdot LinearAlgebra[LinearSolve]\left(Jy, \langle 0, 0, C_A\rangle\right)\right);$$

$$\left[\begin{array}{c} f(R)\, C_A\left(\gamma f(R)+\beta f(R)+\mu_J-2\,T\right) \\ \left(\frac{d}{dR} f(R)\right)\left(-C_J f(R)\,\gamma - C_J f(R)\,\beta + C_A T + C_J T\right) C_A \\ -\left(\frac{d}{dR} f(R)\right) C_A\left(C_A f(R)\,\gamma + C_A f(R)\,\beta + \mu_J C_A + \mu_J C_J - C_A T - C_J T\right) \end{array}\right] \quad (5.1)$$

Determine the (scaled) derivative $\frac{dC_J}{d\mu_A}$:

$$dCJdmuA := collect\left(dydmuA[2], \left[\frac{d}{dR}\, f(R), C_J, C_A\right]\right);$$

$$\left(\left(-\gamma f(R) - \beta f(R) + T\right) C_A C_J + C_A^2\, T\right)\left(\frac{d}{dR} f(R)\right) \quad (5.2)$$

Use the relation between the equilibrium values of C_J and C_A, $C_A = \frac{(\gamma \cdot f(R) - T)}{\mu_A} \cdot C_J$, to eliminate C_J from this equation. This leads to:

$$\left(T - \frac{\mu_A \cdot (\gamma \cdot f(R) + \beta \cdot f(R) - T)}{(\gamma \cdot f(R) - T)}\right) \cdot C_A^2 \cdot \left(\frac{d}{dR} f(R)\right)$$

$$\left(T - \frac{\mu_A\left(\gamma f(R) + \beta f(R) - T\right)}{\gamma f(R) - T}\right) C_A^2 \left(\frac{d}{dR} f(R)\right) \quad (5.3)$$

$$simplify\left(\% - dCJdmuA, \left\{C_J = \frac{\mu_A}{(\gamma \cdot f(R) - T)} \cdot C_A\right\}\right)$$

$$0 \quad (5.4)$$

Clearly juvenile density can increase with an increase in adult mortality if the expression within parentheses is positive. Rewrite this expression in terms of the parameters $m_J = \frac{\mu_J}{T}$, $m_A = \frac{\mu_A}{T}$, $q = \frac{\beta}{\gamma}$, and $h(R) = \frac{\gamma \cdot f(R)}{T}$ and solve the condition in conjuction with the expression for the value of y in equilibrium.

$$JincreaseA := (h(R) - 1) - m_A \cdot ((1 + q) \cdot h(R) - 1)$$

$$h(R) - 1 - m_A\left((1 + q)\, h(R) - 1\right) \quad (5.5)$$

$$solsJmuA := solve(subs(h(R) = hequi2, JincreaseA), q)$$

$$\frac{1}{2}\,\frac{\left(-2\, m_J m_A + m_A + 1 + \sqrt{m_A^2 - 4\, m_A m_J + 2\, m_A + 4\, m_J + 1}\right)\left(m_A - 1\right)}{m_A^2\, m_J - m_A^2 - 1},$$
$$-\frac{1}{2}\,\frac{\left(2\, m_J m_A + \sqrt{m_A^2 - 4\, m_A m_J + 2\, m_A + 4\, m_J + 1} - m_A - 1\right)\left(m_A - 1\right)}{m_A^2\, m_J - m_A^2 - 1} \quad (5.6)$$

Again, there are 2 possible roots, but the second one is strictly negative as can be shown by graphical inspection.

Plot the first root for $q = \frac{\beta}{\gamma}$ as a function of the scaled parameters $m_J = \frac{\mu_J}{T}$ and $m_A = \frac{\mu_A}{T}$. Juvenile density increases with an increase in adult mortality if the parameters are in the parameter space below the colored surface in the 3-dimensional graph below. Notice that the surface does extend above the value $q = \frac{\beta}{\gamma} = 1$, indicating that juvenile density can increase if adults are more efficient than juveniles.

$$plot3d\left(solsJmuA[1], m_J = 0..1, m_A = 0..1, labels = \left[\frac{\mu_J}{T}, \frac{\mu_A}{T}, \frac{\beta}{\gamma} \right], view = [0..1, 0..1, 0..2], orientation = [140, 80, -8] \right);$$

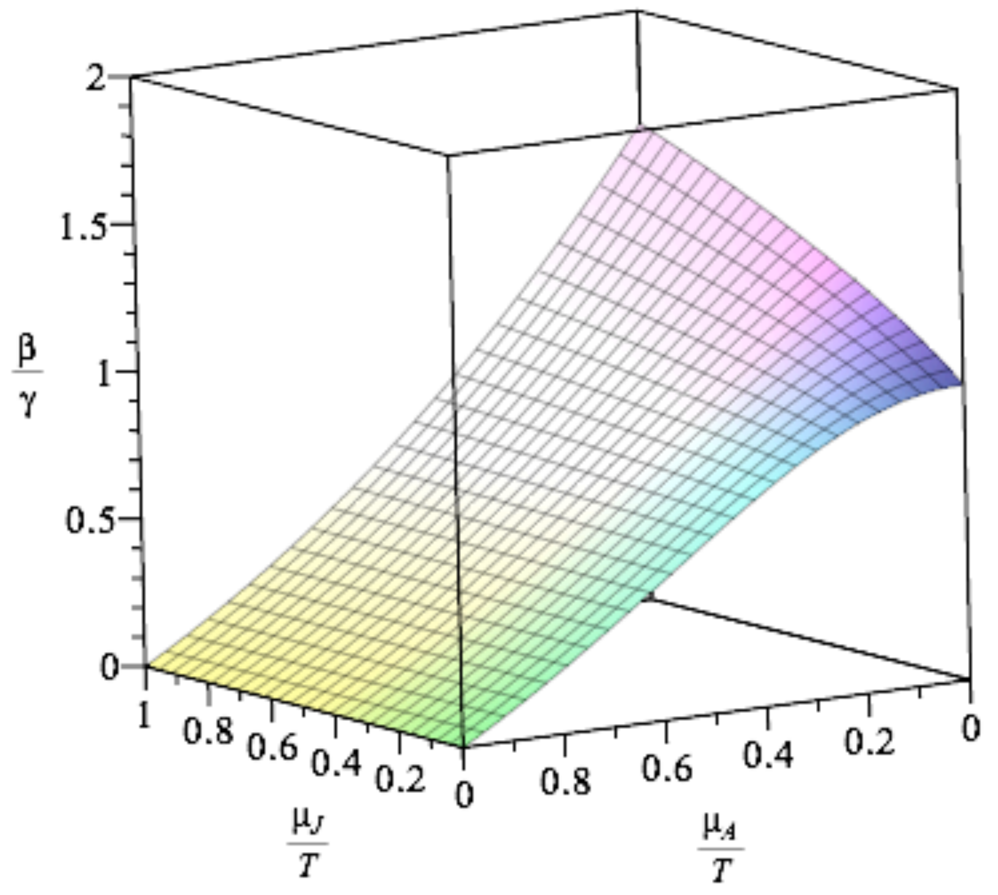

Determine the (scaled) derivative $\frac{dC_A}{d\mu_A}$:

$$dCAdmuA := collect\left(dydmuA[3], \left[\frac{d}{dR} f(R), C_J, C_A \right] \right);$$

$$\left(\left(-\mu_J + T \right) C_A C_J + \left(-\gamma f(R) - \beta f(R) - \mu_J + T \right) C_A^2 \right) \left(\frac{d}{dR} f(R) \right) \qquad \textbf{(5.7)}$$

Use the relation between the equilibrium values of C_J and C_A, $C_A = \frac{(\gamma \cdot f(R) - T)}{\mu_A} \cdot C_J$, to eliminate C_J from this equation. This leads to:

$$\left(\frac{(T-\mu_J)\cdot\mu_A}{\gamma\cdot f(R)-T} - (\gamma f(R) - T + \beta f(R) + \mu_J)\right)\cdot C_A^{\,2}\cdot\left(\frac{\mathrm{d}}{\mathrm{d}R} f(R)\right)$$

$$\left(\frac{(-\mu_J + T)\,\mu_A}{\gamma f(R) - T} - \gamma f(R) - \beta f(R) - \mu_J + T\right) C_A^{\,2} \left(\frac{\mathrm{d}}{\mathrm{d}R} f(R)\right) \quad \textbf{(5.8)}$$

$$simplify\left(\% - dCAdmuA, \left\{C_J = \frac{\mu_A}{(\gamma\cdot f(R) - T)}\cdot C_A\right\}\right)$$

$$0 \quad \textbf{(5.9)}$$

Clearly adult density can increase with an increase in adult mortality if the expression within parentheses is positive. Rewrite this expression in terms of the parameters $m_J = \frac{\mu_J}{T}$, $m_A = \frac{\mu_A}{T}$, $q = \frac{\beta}{\gamma}$, and $h(R) = \frac{\gamma\cdot f(R)}{T}$ and solve the condition in conjuction with the expression for the value of y in equilibrium.

$$AincreaseA := (1 - m_J)\cdot m_A - ((1+q)\cdot h(R) - 1 + m_J)\cdot(h(R) - 1)$$

$$(-m_J + 1)\, m_A - ((1+q)\, h(R) - 1 + m_J)\,(h(R) - 1) \quad \textbf{(5.10)}$$

$$solsAmuA := solve(subs(h(R) = hequi2, AincreaseA), q)$$

$$\frac{1}{2}\,\frac{1}{4 m_A m_J^2 - 6 m_A m_J - 2 m_J^2 + 2 m_A + m_J}\Big(-m_J m_A^2 - 4 m_A m_J^2 + m_J^3 + m_A^2 + 4 m_J m_A - m_J^2 + 3 m_J - 1 + \big(m_A^4 m_J^2 - 8 m_A^3 m_J^3 + 14 m_A^2 m_J^4 - 8 m_A m_J^5 + m_J^6 - 2 m_A^4 m_J + 24 m_A^3 m_J^2 - 52 m_A^2 m_J^3 + 32 m_A m_J^4 - 2 m_J^5 + m_A^4 - 24 m_A^3 m_J + 76 m_A^2 m_J^2 - 56 m_A m_J^3 - m_J^4 + 8 m_A^3 - 52 m_A^2 m_J + 56 m_A m_J^2 + 4 m_J^3 + 14 m_A^2 - 32 m_A m_J - m_J^2 + 8 m_A - 2 m_J + 1\big)^{1/2}\Big), \quad \textbf{(5.11)}$$

$$-\frac{1}{2}\,\frac{1}{4 m_A m_J^2 - 6 m_A m_J - 2 m_J^2 + 2 m_A + m_J}\Big(m_J m_A^2 + 4 m_A m_J^2 - m_J^3 - m_A^2 - 4 m_J m_A + m_J^2 + \big(m_A^4 m_J^2 - 8 m_A^3 m_J^3 + 14 m_A^2 m_J^4 - 8 m_A m_J^5 + m_J^6 - 2 m_A^4 m_J + 24 m_A^3 m_J^2 - 52 m_A^2 m_J^3 + 32 m_A m_J^4 - 2 m_J^5 + m_A^4 - 24 m_A^3 m_J$$

$$+ 76\, m_A^2\, m_J^2 - 56\, m_A\, m_J^3 - m_J^4 + 8\, m_A^3 - 52\, m_A^2\, m_J + 56\, m_A\, m_J^2 + 4\, m_J^3$$

$$+ 14\, m_A^2 - 32\, m_A\, m_J - m_J^2 + 8\, m_A - 2\, m_J + 1\Big)^{1/2} - 3\, m_J + 1\Big)$$

Again, there are 2 possible roots, but the second one is strictly negative as can be shown by graphical inspection.

Plot the first root for $q = \frac{\beta}{\gamma}$ as a function of the scaled parameters $m_J = \frac{\mu_J}{T}$ and $m_A = \frac{\mu_A}{T}$. Adult density increases with an increase in adult mortality if the parameters are in the parameter space above the colored surface in the 3-dimensional graph below. Notice that the surface does not extend below the value $q = \frac{\beta}{\gamma} = 1$, indicating that for adult density to increase with adult mortality adults have to be more efficient than juveniles. As plot makes clear, an emergent Allee effect can occur for a predator that specialises on adult consumers.

$$plot3d\left(solsAmuA[1], m_J = 0\,..0.4, m_A = 0\,..1, labels = \left[\frac{\mu_J}{T}, \frac{\mu_A}{T}, \frac{\beta}{\gamma} \right], view = [0\,..0.4, 0\,..1, 1\,..3], orientation = [140, 80, -8] \right);$$

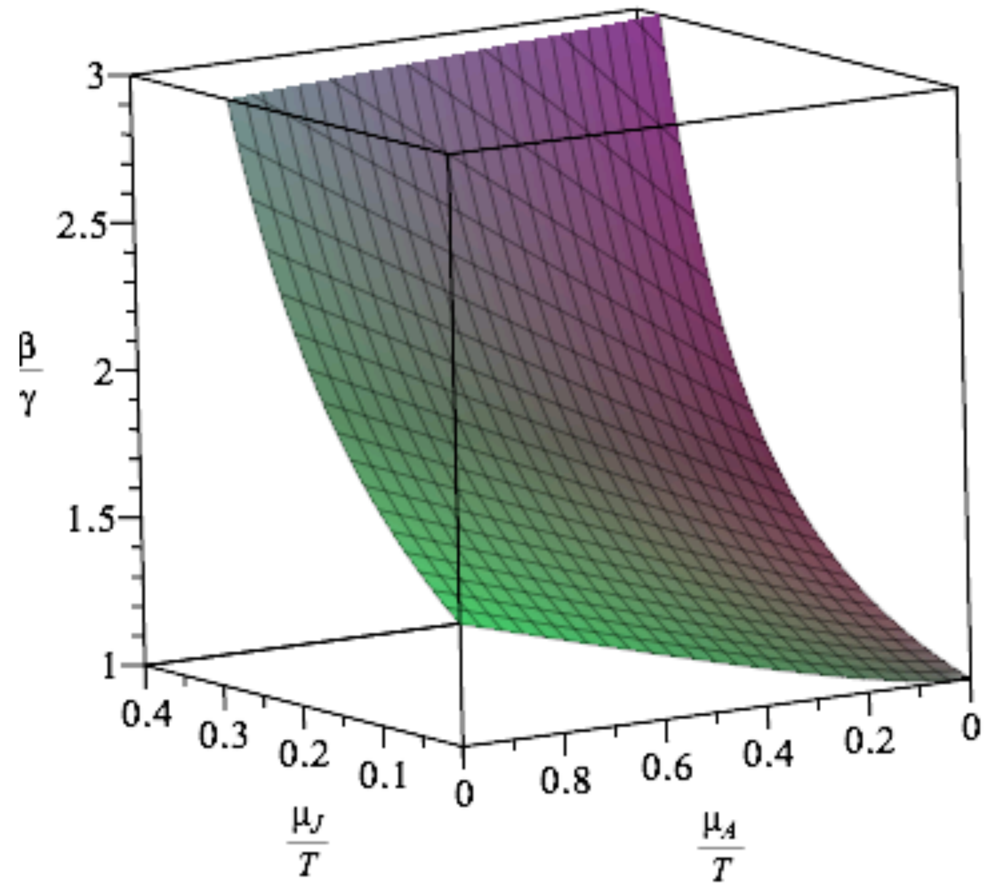

Now plot both the condition for an increase in juvenile density with increasing adult mortality and for an increase in adult density with an increase in adult mortality in the same plot as a function of the scaled parameters $m_J = \frac{\mu_J}{T}$ and $m_A = \frac{\mu_A}{T}$.

$$plot3d\left([solsJmuA[1], solsAmuA[1]], m_J = 0..0.4, m_A = 0..1, labels = \left[\frac{\mu_J}{T}, \frac{\mu_A}{T}, \frac{\beta}{\gamma} \right], view = [0..0.4, 0..1, 0..3], orientation = [140, 80, -8] \right)$$

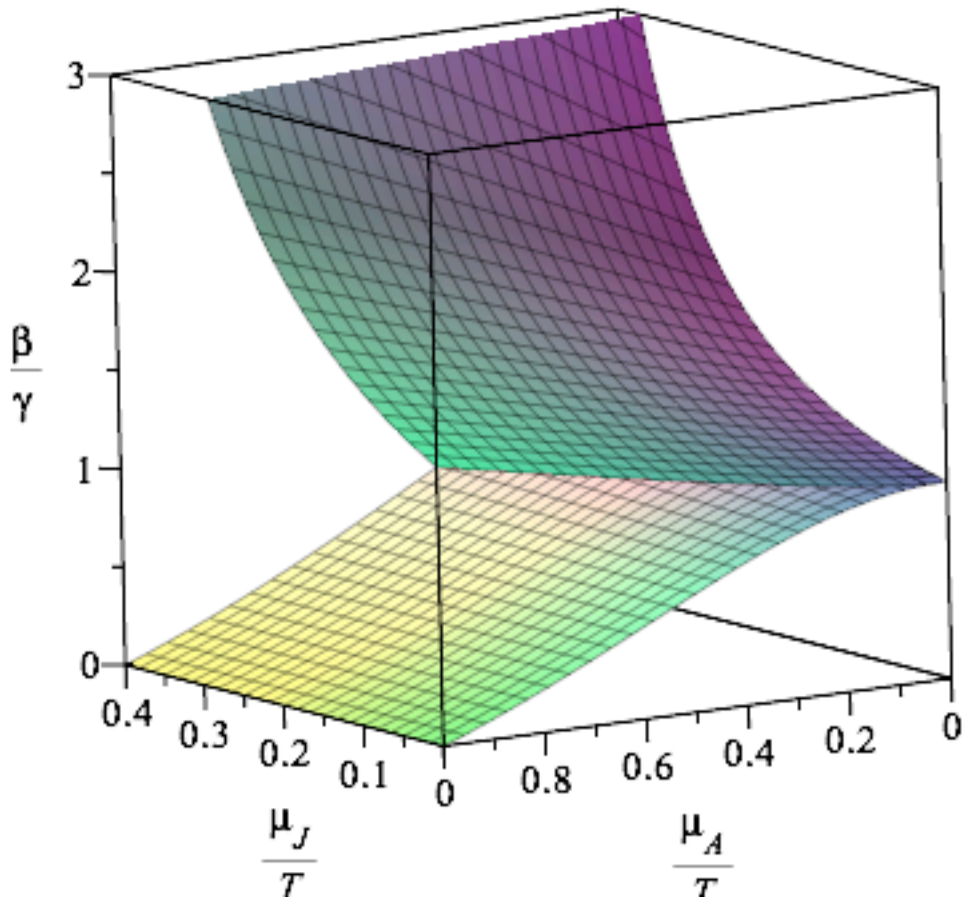

The two resulting surfaces meet at the scaled parameter values $m_J = \frac{\mu_J}{T} = 0$, $m_A = \frac{\mu_A}{T} = 0$ and $q = 1$ but do not intersect each other. Therefore, either juvenile or adult density increases with an increase in adult mortality but not both. Juvenile density mostly increases for values of $q = \frac{\beta}{\gamma} < 1$ whereas adult density increases for values of $q = \frac{\beta}{\gamma} > 1$.
